# Deficit of homozygosity among 1.52 million individuals and genetic causes of recessive lethality

**DOI:** 10.1038/s41467-023-38951-2

**Published:** 2023-06-10

**Authors:** Asmundur Oddsson, Patrick Sulem, Gardar Sveinbjornsson, Gudny A. Arnadottir, Valgerdur Steinthorsdottir, Gisli H. Halldorsson, Bjarni A. Atlason, Gudjon R. Oskarsson, Hannes Helgason, Henriette Svarre Nielsen, David Westergaard, Juha M. Karjalainen, Hildigunnur Katrinardottir, Run Fridriksdottir, Brynjar O. Jensson, Vinicius Tragante, Egil Ferkingstad, Hakon Jonsson, Sigurjon A. Gudjonsson, Doruk Beyter, Kristjan H. S. Moore, Helga B. Thordardottir, Snaedis Kristmundsdottir, Olafur A. Stefansson, Solbritt Rantapää-Dahlqvist, Ida Elken Sonderby, Maria Didriksen, Pernilla Stridh, Jan Haavik, Laufey Tryggvadottir, Oleksandr Frei, G. Bragi Walters, Ingrid Kockum, Henrik Hjalgrim, Thorunn A. Olafsdottir, Geir Selbaek, Mette Nyegaard, Christian Erikstrup, Thorsten Brodersen, Saedis Saevarsdottir, Tomas Olsson, Kaspar Rene Nielsen, Asgeir Haraldsson, Mie Topholm Bruun, Thomas Folkmann Hansen, DBDS Genomic Consortium, Søren Brunak, Kasper Rene Nielsen, Mie Topholm Brun, Hreinn Stefánsson, Unnur Þorsteinsdóttir, Thora Steingrimsdottir, Rikke Louise Jacobsen, Rolv T. Lie, Srdjan Djurovic, Lars Alfredsson, Aitzkoa Lopez de Lapuente Portilla, Soren Brunak, Pall Melsted, Bjarni V. Halldorsson, Jona Saemundsdottir, Olafur Th. Magnusson, Leonid Padyukov, Karina Banasik, Thorunn Rafnar, Johan Askling, Lars Klareskog, Ole Birger Pedersen, Gisli Masson, Alexandra Havdahl, Bjorn Nilsson, Ole A. Andreassen, Mark Daly, Sisse Rye Ostrowski, Ingileif Jonsdottir, Hreinn Stefansson, Hilma Holm, Agnar Helgason, Unnur Thorsteinsdottir, Kari Stefansson, Daniel F. Gudbjartsson

**Affiliations:** 1 deCODE genetics/Amgen, Inc., Reykjavik, Iceland; 2 Faculty of Medicine, School of Health Sciences, University of Iceland, Reykjavik, Iceland; 3 Deptartment of Obstetrics and Gynecology, Copenhagen University Hospital, Hvidovre, Denmark; 4 Department of Clinical Medicine, Faculty of Health, University of Copenhagen, Copenhagen, Denmark; 5 Novo Nordisk Foundation Center for Protein Research, Faculty of Health and Medical Sciences, University of Copenhagen, Copenhagen, Denmark; 6 Methods and Analysis, Statistics Denmark, Copenhagen, Denmark; 7 Institute for Molecular Medicine, Finland, University of Helsinki, Helsinki, Finland; 8 Department of Anthropology, University of Iceland, Reykjavik, Iceland; 9 Department of Public Health and Clinical Medicine, Rheumatology, Umea University, Umea, Sweden; 10 Department of Medical Genetics, Oslo University Hospital and University of Oslo, Oslo, Norway; 11 NORMENT Centre, University of Oslo, Oslo, Norway; 12 KG Jebsen Centre for Neurodevelopmental disorders, University of Oslo, Oslo, Norway; 13 Department of Clinical Immunology, Copenhagen University Hospital, Rigshospitalet, Copenhagen, Denmark; 14 Neuroimmunology Unit, Department of Clinical Neuroscience, Center of Molecular Medicine, Karolinska University Hospital, Karolinska Institutet, Stockholm, Sweden; 15 Department of Biomedicine, University of Bergen, Bergen, Norway; 16 Bergen Center of Brain Plasticity, Division of Psychiatry, Haukeland University Hospital, Bergen, Norway; 17 Icelandic Cancer Registry, Icelandic Cancer Society, Reykjavik, Iceland; 18 Faculty of Medicine, BMC, Laeknagardur, School of Health Sciences, University of Iceland, Reykjavik, Iceland; 19 Division of Mental Health and Addiction, Oslo University Hospital, Oslo, Norway; 20 Centre for Bioinformatics, Department of Informatics, University of Oslo, Oslo, Norway; 21 Danish Cancer Society Research Center, Copenhagen, Denmark; 22 Department of Epidemiology Research, Statens Serum Institut, Copenhagen, Denmark; 23 Norwegian National Centre of Ageing and Health, Vestfold Hospital Trust, Tonsberg, Norway; 24 Department of Geriatric Medicine, Oslo University Hospital, Oslo, Norway; 25 Faculty of Medicine, University of Oslo, Oslo, Norway; 26 Deptartment of Health Science and Technology, Aalborg University, Aalborg, Denmark; 27 Department of Clinical Immunology, Aarhus University Hospital, Aarhus, Denmark; 28 Department of Clinical Medicine, Aarhus University, Aarhus, Denmark; 29 Department of Clinical Immunology, Zealand University Hospital, Koge, Denmark; 30 Department of Clinical Immunology, Aalborg University Hospital, Aalborg, Denmark; 31 Children’s Hospital Iceland, Landspitali University Hospital, Reykjavik, Iceland; 32 Department of Clinical Immunology, Odense University Hospital, Odense, Denmark; 33 Department of Neurology, Copenhagen University Hospital, Rigshospitalet, Glostrup, Denmark; 34 Department of Global Public Health and Primary Care, University of Bergen, Bergen, Norway; 35 Centre for Fertility and Health, Norwegian Institute of Public Health, Oslo, Norway; 36 Institute of Environmental Medicine, Karolinska Institutet, Stockholm, Sweden; 37 Hematology and Transfusion Medicine, Department of Laboratory Medicine, Lund, Sweden; 38 School of Engineering and Natural Sciences, University of Iceland, Reykjavik, Iceland; 39 School of Science and Engineering, Reykjavik University, Reykjavik, Iceland; 40 Department of Medicine, Solna, Karolinska Institutet, Stockholm, Sweden; 41 Department of Mental Disorders, Norwegian Institute of Public Health, Oslo, Norway; 42 Nic Waals Institute, Lovisenberg Diaconal Hospital, Oslo, Norway; 43 PROMENTA Research Center, Department of Psychology, University of Oslo, Oslo, Norway; 44 Analytic and Translational Genetics Unit, Massachusetts General Hospital, Boston, MA USA; 45 Broad Institute of MIT and Harvard, Cambridge, MA USA; 46 Deptartment of Clinical Medicine, Faculty of Health and Medical Sciences, University of Copenhagen, Copenhagen, Denmark

**Keywords:** Genetic predisposition to disease, Disease genetics, Genetics research, Development, Disease genetics

## Abstract

Genotypes causing pregnancy loss and perinatal mortality are depleted among living individuals and are therefore difficult to find. To explore genetic causes of recessive lethality, we searched for sequence variants with deficit of homozygosity among 1.52 million individuals from six European populations. In this study, we identified 25 genes harboring protein-altering sequence variants with a strong deficit of homozygosity (10% or less of predicted homozygotes). Sequence variants in 12 of the genes cause Mendelian disease under a recessive mode of inheritance, two under a dominant mode, but variants in the remaining 11 have not been reported to cause disease. Sequence variants with a strong deficit of homozygosity are over-represented among genes essential for growth of human cell lines and genes orthologous to mouse genes known to affect viability. The function of these genes gives insight into the genetics of intrauterine lethality. We also identified 1077 genes with homozygous predicted loss-of-function genotypes not previously described, bringing the total set of genes completely knocked out in humans to 4785.

## Abstract (web summary)

Genotypes causing pregnancy loss and perinatal mortality are depleted among living individuals and are therefore difficult to find. Here, using genetic data for 1.52 million individuals, the authors identify 25 genes with protein-altering variants exhibiting a strong deficit of homozygosity, suggesting they are essential for successful early development.

## Introduction

The development of whole-genome sequencing technologies has led to a surge in the discovery of sequence variants causing Mendelian diseases^1^. However, the genetic causes of intrauterine lethality remain poorly understood^2^ as our current understanding of sequence variation that causes death of humans is limited to variants where some carriers survive past the early stages of development^3^. There are limited data available on causes of intrauterine lethality^4^, and these often go unnoticed^5^. Genetic causes of loss of blastocyst development, pregnancy loss, and perinatal mortality remain to be thoroughly investigated. A proportion of these pregnancy losses are revealed clinically as miscarriages, while others are unrecognized implantation failures or early pregnancy losses^5^.

Embryonic lethality has been studied in model organisms, and mouse studies suggest that a quarter of homozygous gene knockouts result in embryonic lethality^6,7^. Half of the lethal homozygous mouse knockouts die during early gestation^6,8^ and the majority are estimated to succumb between implantation and gastrulation^9^.

To date, four studies have reported 3527 autosomal genes with rare biallelic predicted loss-of-function (pLOF) sequence variants (i.e. genes knocked out in humans) that are valuable for assessing physiological and pathological consequences of gene loss-of-function^10–13^. Two of these involved populations of Pakistani origin with a high rate of parental relatedness^10,11^, which reduces the number of individuals that need to be sequenced to detect homozygous genotypes of rare variants. In combined data from these two studies, a total of 13,725 exome sequenced individuals had 1829 genes completely knocked out, of which the majority (>68%) were knocked out in just one individual and where the mean frequency of the pLOF variants was ~0.2%. The remaining two studies involved more outbred populations^12,13^ where the minority (<34%) of knocked-out genes were observed in just one individual and the mean frequency of the pLOF variants was ~0.5%. In the GnomAD database, 1825 genes are knocked out among 15,708 whole-genome and 125,748 exome sequenced individuals, primarily of European origin^13^. Finally, in a previous study of 104,220 Icelanders, we observed 6795 pLOF sequence variants in 4924 autosomal genes, detected through whole-genome sequencing of 2636 individuals, and identified 1151 genes with homozygous pLOF genotypes^12^. There, we also reported a deficit of both double transmissions of pLOFs^12,14^ from pairs of heterozygous parents and a deficit of homozygosity of pLOF variants relative to their allele frequency in the population, where the greatest deficit was observed for a splice acceptor variant in *DHCR7* in the Icelandic population^12,14^. Cataloging genes with a strong deficit of homozygosity for protein-altering variants in human populations provides insights into potential causes of embryonic and fetal death, stillbirth, death in infancy, or under-sampling because of morbidity^15^. In a randomly-mating population, a rare variant present in one per five hundred individuals is expected to be present in one per million in a homozygous state. Consequently, detection of rare homozygous genotypes requires large sample sizes. To date, studies have been limited by sample sizes on the order of 100 thousand individuals, which are not well powered to detect rare homozygous genotypes through testing for deviation from Hardy-Weinberg equilibrium (HWE) expectations.

In this work, we identified sequence variants with a strong deficit of homozygosity when taking into account the number of heterozygotes and assuming HWE in a set of 1.52 million North-Western Europeans, an order of magnitude more than in our previous study^12^. We examine and report genotype counts for both moderate impact (missense, in-frame indels, splice region sequence variants) and pLOF variants (stop-gained, frameshift, essential splice donor, and acceptor sequence variants), and we also combined the pLOF variants in a gene test. To determine whether sequence variants with a strong deficit of homozygosity resulted from losses early or late in pregnancy, we examined the reproductive history of heterozygous carrier couples, looking for an excess of miscarriages or early death among their offspring. Also, we assessed the effects of such variants on RNA and protein levels in heterozygous carriers to provide experimental validation of their functional effect. Finally, we compared the set of genes with a deficit of pLOF homozygosity to experimental data on the viability of mouse knockouts and the critical role of these genes in cell growth.

## Results

### Deficit of homozygosity

**Fig. 1:**
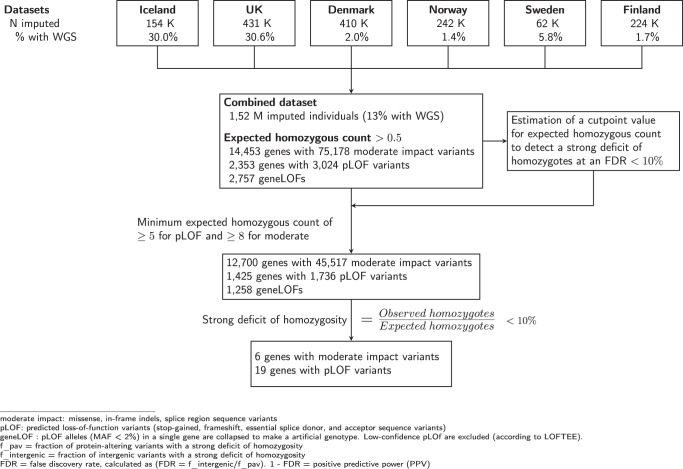
Flowchart depicting the study design to detect homozygosity deficit in 1.52 million North-Western Europeans. We looked for a strong deficit of homozygosity (10% or less of predicted homozygotes based on observed heterozygote frequency and the assumption of Hardy-Weinberg equilibrium within populations) among protein-altering sequence variants in a meta-analysis of 1.52 million individuals from six populations. We tested 75,178 moderate-impact and 3024 pLOF single variants for the deficit of homozygosity based on the imputation of variants detected by whole-genome sequencing of individuals from all of the populations. Additionally, a gene-based test (geneLOF) for the deficit of homozygosity was performed, where we were able to test 2757 genes for deficit of homozygosity. To estimate a false discovery rate, we divided the fraction of intergenic sequence variants with strong deficits of homozygosity by that of protein-altering sequence variants to determine a cutpoint value for expected homozygous count to detect a strong deficit of homozygosity at an FDR < 10%.

We looked for a strong deficit of homozygosity among protein-altering sequence variants in a meta-analysis of 1.52 million individuals from six populations (Denmark, Iceland, Norway, Sweden, Finland, and the UK). This was based on the imputation of variants detected by whole-genome sequencing of individuals from all of the populations (Fig. 1 and Supplementary Data 1). Of the study participants, 197,146 were whole-genome sequenced (Supplementary Data 1).

We tested 75,178 moderate-impact variants in 14,453 genes and 3024 pLOF variants in 2353 genes (Fig. 1, Supplementary Data 2, and Supplementary Data 3). Of the 3024 pLOF variants, 730 were rated as low-confidence pLOFs by the LOFTEE algorithm (Loss-Of-Function Transcript Effect Estimator)^13^, leaving 2294 pLOF variants in 1837 genes. A summary of the 75,178 moderate-impact and 3024 pLOF single variants tested is provided as supplementary data (Supplementary Data 4). Additionally, we performed a gene-based test for the deficit of homozygosity, where we created a single biallelic genotype for each gene, indicating whether 0, 1 or both haplotypes in an individual are affected by at least one pLOF variant with a MAF under 2%, excluding the variants flagged as low-confidence by LOFTEE. We refer to such genotypes as geneLOF, and in this way, we were able to test 2757 genes for deficit of homozygosity (Supplementary Data 5 and Supplementary Data 6).

It is well established that deviations from random mating within a population (such as inbreeding or stratification) tend to increase the number of homozygotes^16^. For sequence variants that increase the risk of deleterious phenotypes among homozygotes, these factors will therefore tend to increase the number of individuals who are exposed to negative selection. In our data, there is an excess of observed homozygotes compared to the expected number of homozygotes under HWE, with rare variants showing the greatest relative excess (Supplementary Fig. 1 and Supplementary Data 2). Based on the genomic inbreeding coefficient, less than 0.71% of study participants are first cousins, or more closely related (Supplementary Data 1). We identified 70,721 individuals (5.4%) who had homozygous geneLOFs (i.e. both parental chromosomes harbor a pLOF variant in the same gene with MAF < 2%) in a combined set of 1.30 million genotyped individuals (excluding the Finnish data set, where individual genotype data was not available). Of the 70,721 individuals with a knockout, 66,727 (94.4%) were predicted to have just one gene knocked out. A total of 2671 genes were knocked out based on geneLOFs in the meta-analysis of all 1.52 million individuals (Supplementary Data 6, and 7). We observed two or more knockouts for 1722 of these 2671 genes (66.3%). In total 1077 of the identified genes have not been reported in previous publications^10–13^. Combining the data on knockouts from the current and previous studies^10–13^, yields 4785 knocked-out genes, of which 42 are observed in all datasets (Supplementary Data 7).

**Fig. 2:**
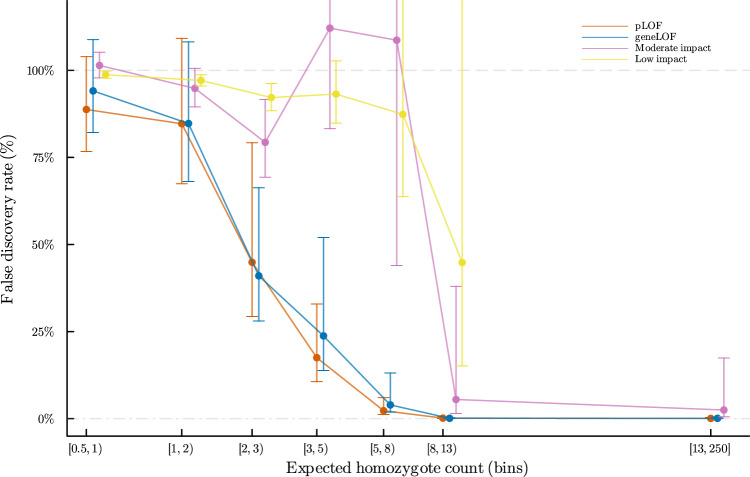
False discovery rate (FDR) for a strong deficit of homozygosity relative to intergenic variants in the combined set of 1.52 million individuals of North-Western European descent (Denmark, Finland, Iceland, Norway, Sweden, and the UK). After binning variants based on the expected number of homozygotes and functional impact, the fraction of protein-altering variants with a strong deficit of homozygosity (f_pav) in each bin was compared to that of intergenic variants (f_intergenic) to estimate an FDR (FDR = f_intergenic/f_pav). One minus the FDR estimates the fraction of homozygous deficit variants within each bin due to negative selection (1 - FDR = positive predictive power (PPV) = 1 - f_intergenic/f_pav). FDR confidence intervals were calculated using the AECI method.

We considered a variant to have a strong deficit of homozygosity if we observed 10% or less of predicted homozygotes^17,18^ based on observed heterozygote frequency and the assumption of HWE within populations (Supplementary Fig. 2, Supplementary Data 2, and 5). Variants with a less marked deficit are presented in the section “Incomplete homozygous deficit” in the Supplementary Discussion. pLOF and moderate impact sequence variants have the greatest predicted functional impact and are most likely to affect health and viability^19^. At the other end of the spectrum are intergenic variants, that have the lowest predicted functional impact^19^. Therefore, to increase power to detect deficit of homozygotes we calibrated our expectation of homozygous protein-altering variants under neutrality and compare the deficit of homozygous genotypes of protein-altering variants to that of intergenic variants. After binning variants based on the expected number of homozygotes under HWE and functional impact, we compared the fraction of protein-altering variants (f_pav) with a strong deficit of homozygosity in each bin to the intergenic one (f_intergenic) to derive a false discovery rate (FDR = f_intergenic/f_pav) (Fig. 2, Supplementary Data 2, and 5). One minus the FDR estimates the fraction of homozygous deficit variants within each bin due to negative selection rather than by chance, under the assumption that homozygosity for intergenic variants is effectively neutral (1 - FDR = positive predictive power (PPV) = 1 - f_intergenic/f_pav).

pLOF variants with five or more expected homozygotes had an FDR under 3% (Fig. 2, and Supplementary Data 2). Five or more homozygotes were expected for 1736 pLOF variants in 1425 genes. Of these, 16 variants in as many genes were deemed to have a strong deficit of homozygosity (Table 1). The FDR for moderate impact variants with eight or more expected homozygotes was under 6%, and of these, six variants had a strong deficit of homozygosity (Fig. 2, Table 1, and Supplementary Data 2). In comparison, using Bonferroni correction for multiple testing, five variants had a significant deficit for homozygosity for pLOF (*P* < 0.05/1736 = 2.9 × 10^−5^, assuming Poisson distribution) and two for moderate impact variants (*P* < 0.05/47,429 = 1.1 × 10^−6^, assuming Poisson distribution) (Supplementary Data 2). No low-impact variants had a significant strong deficit of homozygosity after accounting for multiple testing. No deficit of homozygosity was observed for variants with an expected homozygote count above 250, or minor allele frequency (MAF) above ~1.4% (Supplementary Data 2).

**Table 1:** Genes carrying sequence variants displaying significant deficit of homozygosity

		Homozygote count: by dataset			Homozygote count: combined			Gene: functional annotation			Significant single variants: functional annotation^a^			
Gene	Test^b^	Datasets	O	E	O	E	O/E	OMIM ID (inheritance)	KO mouse	Human cell-line	Pos (Hg38)^c^	Ref/Alt	Consequence (HGVS)	ClinVar (*N*)
**Loss-of-function variants**														
*DHCR7*	G,V	I/D/N/S/U/F	0/0/0/0/0/0	31/11/3.5/0.74/53/0.82	0	100	0	270400 (AR^c^)	Lethal	Non-Essential	chr11:71,435,840	C/G	Splice acceptor c.964-1 G > C	LP(2),P(27)
*TSFM*	G,V	I/D/N/S/U/F	0/1/0/0/0/0	3e-5/0.11/0.04/0.088/0.044/29	1	29.2	0.034	610505 (AR^c^)	Lethal	Essential	chr12:57,796,461	C/T	Stop gained p.Gln307Ter	B(1),LP(1),P(4)
*CCDC59*	G,V	I/D/N/S/U/F	0/0/0/0/0/0	4/6.7/4.6/0.73/4.5/1.2	0	21.6	0		Lethal E9.5	Essential	chr12:82,354,490	CTTAC../C	Splice donor c.561_564+4del	
*ATP5PB*	G,V	I/N	0/0	16/0.00064	0	15.8	0		Lethal	Essential	chr1:111,459,496	C/T	Stop gained p.Arg185Ter	
*MTG2*	G,V	I/D/N/S/U/F	0/0/0/0/0/0	0.3/0.77/0.16/0.49/0.82/11	0	13.4	0		Lethal E9.5	Essential	chr20:62,198,729	AG/A	Frameshift p.Gly191AlafsTer14	
*BRF2*	G,V	I/N/S/U	0/0/0/0	11/0.0075/0.00041/0.019	0	11.1	0			Essential	chr8:37,848,595	C/T	Splice donor c.214+1 G > A	
*GTF2H3*	G,V	I/D/N/S/U/F	0/0/0/0/0/0	1.7/4.4/0.9/1.4/1.9/0.4	0	10.7	0			Essential	chr12:123,633,862	G/A	Start lost p.Met1?	
*CENPF*	G	I/D/N/S/U	0/0/0/0/0	9.1/0.0071/0.34/0.096/1	0	10.5	0	243605 (AR^c^)	Viable	Non-Essential				
*PUM3*	G,V	I/D/N/S/U	0/0/0/0/0	8.1/0.44/0.047/0.018/1.7	0	10.2	0			Non-Essential	chr9:2,837,222	ATT/A	Frameshift p.Lys87IlefsTer12	
*ELOF1*	G,V	I/N/S/U	0/0/0/0	10/0.064/0.0059/0.00076	0	10.2	0		Lethal E12.5	Non-Essential	chr19:11,554,278	AC/A	Frameshift p.Gln23HisfsTer15	
*PKHD1*	G,V	I/D/N/S/U/F	0/0/0/0/0/0	4/0.07/0.0036/0.0015/0.49/5.1	0	9.62	0	263200 (AR^c^)	Sub-Viable	Non-Essential	chr6:52,058,349	G/A	Stop gained p.Arg496Ter	LP(1),P(10)
*RPAP2*	G,V	I/D/N/S/U	0/0/0/0/0	9.1/0.0052/0.0091/0.0013/0.11	0	9.26	0			Essential	chr1:92,333,464	TGAGT../T	Frameshift p.Lys512ValfsTer20	
*WARS2*	G,V	I/U	0/0	9.1/0.0081	0	9.10	0	617710 (AR)	Lethal E9.5	Essential	chr1:119,033,158	TG/T	Frameshift p.His279MetfsTer4	
*PNKP*	G,V	I/D/N/S/U	0/0/0/0/0	2.5/2.3/1.2/0.13/2.4	0	8.53	0	613402 (AR),616267 (AR)	Lethal	Essential	chr19:49,862,369	A/G	Splice donor c.1029+2 T > C	LP(2),P(2)
*BRIP1*	G	I/D/N/S/U	0/0/0/0/0	7.5/0.0056/0.043/0.00026/0.21	0	7.74	0	114480 (AD),609054 (AR)	Viable	Essential				
*GBE1*	G,V	I/D/N/S/U	0/0/0/0/0	1.8/2.2/2/0.33/1	0	7.29	0	232500 (AR^c^)	Lethal	Non-Essential	chr3:81,648,854	A/G	Splice donor c.691+2 T > C	P(11)
*AGK*	G,V	I/D/U	0/0/0	5.8/0.00055/0.042	0	5.79	0	212350 (AR^c^)	Sub-Viable	Non-Essential	chr7:141,649,323	A/ATAAC	Frameshift p.Ile348AsnfsTer38	
*CDC7*	G,V	I/U	0/0	5.3/0.0045	0	5.34	0		Lethal E9.5	Essential	chr1:91,520,185	T/G	Stop gained p.Tyr412Ter	
*DIAPH3*	G	I/D/N/S/U	0/0/0/0/0	1.3/1.2/2.3/0.058/0.44	0	5.29	0	609129 (AD)	Lethal	Non-Essential				
**Moderate impact**														
*MRPS30*	V	I/D/N/S/U/F	0/1/0/0/0/0	2.8/7.6/7.5/1/4.9/24	1	48.2	0.021			Essential	chr5:44,811,105	T/G	Missense p.Ile233Arg	
*PMM2*	V	I/D/N/S/U/F	0/0/2/0/2/0	5.2/15/6.3/1.6/15/11	4	54.1	0.074	212065 (AR)	Lethal E9.5	Non-Essential	chr16:8,811,153	G/A	Missense p.Arg141His	LP(2),P(29)
*HYLS1*	V	F	1	15	1	14.9	0.067	236680 (AR)	Lethal E15.5	Non-Essential	chr11:125,900,000	A/G	Missense p.Asp211Gly	P(6)
*MVD*	V	I/D/N/S/U	0/0/0/0/0	0.85/2/0.58/0.34/7.3	0	11.1	0	614714 (AR)	Lethal E9.5	Essential	chr16:88,663,006	C/T	Splice region c.70+5 G > A	
*GLE1*	V	I	0	11	0	11.0	0	253310 (AR),611890 (AR)	Lethal	Essential	chr9:128,536,414	G/A	Missense p.Arg569His	LP(2),P(2)
*CASP9*	V	I/D/N/S/U/F	0/0/0/0/0/1	1.2/3.9/1.6/0.72/2.3/2.2	1	11.9	0.084		Lethal	Non-Essential	chr1:15,506,000	T/G	Missense p.His237Pro	
**Total**	—	—	—	—	8	452	—	—	—	—	—	—	—	—

Loss-of-function variants: stop-gained, frameshift, essential splice donor and acceptor sequence variants; Moderate impact: missense, inframe indel, splice region sequence variants; Ref/Alt: reference and alternative alleles, alleles sequences over five base pairs are depicted as “..”; Consequence (HGVS): calculated variant consequence using VEP and sequence variant description according to HGVS nomenclature; E: number of homozygotes expected under HWE, O: number of homozygotes observed; DATASETS: I = Iceland, D = Denmark, N = Norway, S = Sweden, U = UK Biobank, F = Finland (Finngen R5); ClinVar (N): Clinical significance of reported as pathogenic in ClinVar and N is the number of submitted records in parenthesis, P = pathogenic, LP = likely pathogenic.; OMIM ID (Inheritance): OMIM ID of mendelian disease linked to gene, early lethality is indicated with a footnote. The mode of inheritance is indicated in parenthesis.; KO mouse: Homozygous knockout mouse mortality phenotypes (MGI and IMPC). Lethal = complete embryonic lethality, Sub-Viable = incomplete penetrance of embryonic lethality, Viable = KO mouse viable, empty = Human gene ortholog not targeted in mouse (see Supplementary Data 1 for details); Human cell-line: Genes essential for cell growth in human cell lines (DepMap). Essential = essential for cell growth in human cell-lines, Non-Essential = Non-essential for cell growth in human cell-lines. (see Supplementary Data 4 and 6 for details).

^a^Annotation is shown for variants that are significant on a single-variant test, see Supplementary Data 4.

^b^Significant homozygous deficit: G = gene-based test, V = single-variant test.

^c^Early lethality reported (see Supplementary Data 4).

geneLOFs with five or more expected homozygotes had an FDR under 4% (Fig. 2 and Supplementary Data 5). Five or more homozygous individuals were expected for 1258 geneLOFs and nineteen of these genes had a strong deficit of homozygosity (Table 1). If we determined significance based on deviation from HWE and use Bonferroni correction for multiple testing (*P* < 0.05/1258 = 4 × 10^−5^, assuming Poisson distribution), ten genes had a significant deficit of homozygosity (Supplementary Data 5).

In total, we identified 25 genes with protein-altering variants with a strong deficit of homozygosity; nineteen involving pLOF variants, and six involving moderate impact variants (Table 1 and Supplementary Data 8). The allele frequency distribution of the underlying pLOF and missense variants ranges from <0.001% to 1.4% across the six populations and are detectable but rarer in publicly available exome and genome sequence databases (Table 1, Supplementary Fig. 3, and Supplementary Data 9) (see Supplementary Discussion for details). Among the 25 genes harboring variants with a strong deficit of homozygosity, 11 are located in genes that have not been reported to cause a Mendelian condition (Table 1). The remaining 14 genes are reported to have variants causing a Mendelian condition (12 under a recessive mode of inheritance, two under a dominant mode), and in ten instances the variant in question has been observed in genotypes classified as pathogenic or likely pathogenic in the ClinVar database^20^ (Supplementary Data 4, and 10) (see Supplementary Discussion for details).

### Effect of variants with a strong deficit of homozygosity on gene expression

We assessed the impact of variants with a significant deficit of homozygosity on RNA splicing (sQTL), mRNA levels (eQTL), and protein levels (pQTL) in the Icelandic population, based on RNA sequencing of blood samples from 17,848 individuals and plasma protein levels measured with 4907 aptamers (SOMAscan) in 35,559 individuals^21^. We found that the variants in ten of the genes with a strong deficit of homozygosity were in high LD (r^2^ from 0.8 to 1.0) with five lead sQTLs, six lead cis-eQTLs, and three lead cis-pQTLs (Supplementary Data 11, 12, and 13).

In *ATP5PB*, the stop gained variant p.Arg185Ter is the lead eQTL for *ATP5PB*, and is associated with reduced blood mRNA levels (*P* < 1 × 10^−300^, effect = −2.5 SD), consistent with nonsense-mediated decay (Supplementary Fig. 4). The splice donor variant c.561_564+4delACAAGTAA in *CCDC59* causes a skipping of the third exon of this gene (effect = 2.7 SD, *P* = 3.0 × 10^−229^) inducing a frameshift (Supplementary Fig. 4). The start loss variant in *GTF2H3* associates with reduced expression (*P* < 1.3 × 10^−30^, effect = −1.3 SD) over all exons consistent with a loss-of-function effect (Supplementary Fig. 4).

In our data, the splice region variant c.70+5 G > A associated with reduced mRNA levels of *MVD* (encoding Diphosphomevalonate decarboxylase; ERG19) in blood (effect = −0.56 SD, *P* = 7.9 × 10^−7^), and was a lead cis-pQTL for MVD in plasma (effect = −0.77 SD, *P* = 5.0 × 10^−22^) (Supplementary Fig. 5). Heterozygosity of this variant is associated with a high risk of congenital malformations of skin in the UK Biobank (ICD10 code Q82; 1464 cases and 429,474 controls) (MAF_UK_ = 0.41%, OR = 6.8, *P* = 1.2 × 10^−36^). This association is consistent with autosomal dominant form of porokeratosis reported in OMIM (OMIM:614714). Diphosphomevalonate decarboxylase is an enzyme involved in cholesterol biosynthesis that catalyzes the conversion of mevalonate pyrophosphate into isopentenyl pyrophosphate. Thus, among heterozygotes, reduced dosage increases the risk of malformations of the skin but does not impact life expectancy. On the other hand, homozygosity for the *MVD* splice region variant likely reduces enzymatic activity to levels not compatible with life.

We also confirmed the previously described effects of four homozygous deficit variants reported as disease-causing on RNA and protein levels: c.964-1 G > C in *DHCR7* activates a cryptic splice-site resulting in a 134 base pair intron retention that leads to a frameshift^22^, c.691+2 T > C in *GBE1* leads to skipping of exon five^23^, p.Arg141His in *PMM2* leads to reduced levels of Phosphomannomutase 2 encoded by *PMM2*^24^, and c.1029+2 T > C *PNKP*^25^ introduces a retained intron resulting in skipping of exon 10 (Supplementary Fig. 5).

### Gene set over-representation analysis

Experimental data on the viability of mouse knockouts, and the essentiality of genes for the growth of human cell lines is valuable to infer the gestational timing of pregnancy loss^26,27^. To gain a better understanding of the biology behind a strong deficit of homozygosity, we performed a gene set over-representation analysis using three different data sets: genes harboring variants reported to cause recessive Mendelian disease, genes essential for growth of human cell lines identified through genome-wide screens, and orthologous mouse genes known to affect viability (Table 2, Supplementary Data 14, 15, and 16).

**Table 2:** Gene set over-representation analysis of genes with deficit detected through a gene-based test of pLOFs (geneLOFs)

	Expected homozygous count [1-5)				Expected homozygous count >=5			
GENE SET	OBS/EXP < = 0.1	OBS/EXP > 0.1	*P*-value^a^	OR [95%CI]^a^	OBS/EXP < = 0.1	OBS/EXP > 0.1	*P*-value^a^	OR [95%CI]^a^
**OMIM**								
*AR OMIM*	39 (39.4%)	103 (14.2%)	*1.6 × 10*^*−8*^	3.92 [2.42–6.32]	9 (47.4%)	147 (12.0%)	*1.9 × 10*^*−4*^	6.56 [2.32–18.3]
*Other*	60 (60.6%)	623 (85.8%)			10 (52.6%)	1074 (88.0%)		
Total	99	726	—	—	19	1221	—	—
**Human cell-line**								
*Essential*	31 (33.7%)	30 (4.7%)	*1.5 × 10*^*−14*^	10.2 [5.54–18.7]	11 (57.9%)	85 (8.3%)	*9.1 × 10*^*−8*^	15.1 [5.39–44.6]
*Non-essential*	61 (66.3%)	603 (95.3%)			8 (42.1%)	942 (91.7%)		
Total	92	633	—	—	19	1027	—	—
**KO mouse**								
*Lethal or subviable*	40 (57.1%)	104 (23.4%)	*3.6 × 10*^*−8*^	4.36 [2.51–7.64]	13 (86.7%)	179 (24.9%)	*1.2 × 10*^*−6*^	19.5 [4.35–179]
*Viable*	30 (42.9%)	341 (76.6%)			2 (13.3%)	539 (75.1%)		
Total	70	445	—	—	15	718	—	—

The analysis was performed using three different data sets: genes harboring variants reported to cause recessive Mendelian disease, genes essential for the growth of human cell lines identified through genome-wide screens, and orthologous mouse genes known to affect viability.

GENE SET: number of geneLOFs with an expected homozygous count of (1,5] and >= 5 (see Supplementary Data 6 for details)

OBS/EXP < = 0.1: deficit of homozygosity defined as a ratio of observed to expected homozygous count less than 0.1 among genes with an expected homozygous count over five; OBS/EXP > 0.1: ratio of observed to expected homozygous count over 0.1 among geneLOFs with expected homozygous count over five; EXP: number of homozygotes expected under HWE; OBS: number of homozygotes observed.

OR [95%CI]: Odds-ratio [95% confidence interval].

OMIM: *AR OMIM*: Gene linked to a mendelian disease linked with an autosomal recessive mode of inheritance in OMIM (see Supplementary Data 14 for details). *Other*: genes not known to harbor variants reported to cause recessive Mendelian disease.

KO mouse: Homozygous knockout mouse mortality phenotypes (see Supplementary Data 16 for details). *Lethal or subviable*:  absence of live knockout (null) homozygote pups or fewer than 12.5% live knockout pups (half of the 25%). *Viable*: homozygous (null and wild type) and heterozygous pups are observed in the same or more than the expected normal Mendelian ratios.

HUMAN CELL-LINE: Gene essentiality status for cell growth in human cell lines (DepMap). (see Supplementary Data 15 for details). *Essential*: If a gene’s inactivation significantly impairs a cell’s growth, it is categorized as an essential gene. *Non-essential*: When a gene’s inactivation does not significantly impair cell growth, it is considered a non-essential gene.

^a^Significance level based on the Fisher test.

Among the 1258 genes with geneLOFs expected to have five or more homozygotes, 96 are essential for cell growth, and 192 are lethal when knocked out in mice (Table 2). The fraction of genes with a homozygous deficit among those essential for cell growth was 11.5% (11/96), and those that are mouse lethal was 6.8% (13/192). Compared to geneLOFs that did not show a homozygous deficit, those with a homozygous deficit are 6.6-fold more likely to be linked to autosomal recessive disease (*P* = 1.9 × 10^−4^), 15.1-fold more likely to be essential for viability in human cell lines (*P* = 9.1 × 10^−8^), and 19.5-fold more likely to result in lethality when knocked out in mice (*P* = 1.2 × 10^−6^) (Table 2). Thus, pLOF variants in genes with a strong deficit of homozygosity may cause pre-natal lethality rather than a post-natal disorder. Furthermore, based on being essential for growth of human cell lines, 13 genes with a strong deficit of homozygosity are candidates for harboring variants that lead to early pregnancy loss (see Supplementary Discussion for details).

geneLOFs with an expected homozygote count between one and five were also enriched in these datasets, although not to the same extent (Table 2, Supplementary Data 17 and 18). This shows that we only have statistical power to detect the subset of such variants in the combined set of 1.52 million individuals with a MAF of at least 0.2% (pLOF: MAF ≥ 0.18% corresponding to an expected homozygous count of 5, moderate impact variants: MAF ≥ 0.23% corresponding to an expected homozygous count of 8) (Supplementary Fig. 6). It has been suggested that the majority of recessive lethal variants are very rare and likely rarer than those identified in the current study^15^.

### Effect of variants with a strong deficit of homozygosity on pregnancy loss in the Icelandic population

To determine whether a strong deficit of homozygosity is the result of early infant death or increased rate of miscarriage, we identified 140 Icelandic couples who are carriers of pLOF variants in 15 of the homozygous deficiency genes when restricting to genes where the sum of pregnancies (miscarriage or registered birth) of all carrier couples is at least two. These couples have a one-in-four chance of producing a zygote that is a homozygote for the pLOF they carry. Carrier mothers were at increased risk of ever experiencing a miscarriage if the father was a carrier compared to mothers from non-carrier couples matched on year of birth and number of pregnancies (OR = 1.93 [95% CI: 1.35–2.74], *P* = 2.4 × 10^4^, *N* couples = 140, *N* miscarriage = 57) (Table 3 and Supplementary Data 19). Consistent with a recessive inheritance pattern, couples, where one partner was a carrier, were not more likely to experience a miscarriage (OR = 1.0 [95% CI: 0.96–1.05], *P* = 0.92, *N* couples = 12,915, *N* miscarriage = 3398) (Supplementary Data 19). The most significant effect on miscarriage was observed for couples carrying pLOF variants in *DHCR7* and was significant after correcting for 15 genes being tested (OR = 5.3 [95% CI: 2.0–16], *P* = 1.9 × 10^−4^ < 0.05/15), although we could not show an excess of miscarriage for any other gene individually (Table 3). Couples carrying pLOF variants in the remaining 14 genes also had an excess of miscarriages (OR = 1.6 [95% CI: 1.3–2.7], *P* = 0.012, *N* couples = 119, *N* miscarriage = 43) (Table 2). We came to the same conclusion by comparing the number of pregnancies that result in miscarriage between mothers from carrier couples and controls (Supplementary Data 19).

**Table 3:** Excess miscarriage in Icelandic couples that are carriers of homozygous deficit pLOF variants among 61,848 genotyped couples from Iceland were the female partner answered a routine pregnancy history questionnaire in a healthcare setting between 1964 and 1994

Gene	*N* couples	*N* children (death < 2 YOA)	Miscarriage ever	OR [95% CI]	*P*-value^a^
*DHCR7*	21	51 (2)	14	5.34 [2.02–15.7]	1.9 × 10−^4^
*BRIP1*	7	16 (1)	5	7.13 [1.16–74.9]	0.015
*RPAP2*	6	24 (1)	4	3.21 [0.59–17.3]	0.097
*PUM3*	9	21 (0)	5	2.67 [0.57–13.5]	0.16
*BRF2*	17	45 (1)	8	1.96 [0.66–5.72]	0.19
*CCDC59*	8	18 (0)	4	2.60 [0.48–14.0]	0.23
*GBE1*	2	4 (0)	1	3.14 [0.04–247]	0.42
*ELOF1*	13	28 (0)	4	1.52 [0.34–5.45]	0.51
*ATP5BP*	24	54 (0)	7	1.16 [0.41–2.94]	0.82
*AGK*	6	15 (3)	1	0.78 [0.017–6.99]	1.00
*CDC7*	2	5 (0)	1	1.83 [0.023–144]	1.00
*CENPF*	9	26 (0)	2	0.77 [0.078–4.03]	1.00
*PKHD1*	5	12 (2)	1	0.80 [0.016–8.14]	1.00
*PNKP*	5	11 (0)	1	0.84 [0.017–8.50]	1.00
*WARS2*	8	17 (0)	1	0.57 [0.012–5.12]	1.00
*Total: all*	140	342 (10)	57	1.93 [1.35–2.74]	2.4 ×10−^4^
*DCHR7 removed*	119	291 (8)	43	1.63 [1.09–2.40]	0.012

*N* couples: Number of carrier couples where both partners are carriers, gene: geneLOFs, Miscarriage ever: number of mothers having ever experienced a miscarriage Miscarriage events: number of miscarriages experienced by mothers.

^a^Fisher s exact test was used to assess excess miscarriage for the number of mothers who experienced at least one miscarriage compared to never between carriers couples (Carrier Father and carrier mother) and non-carrier control couples matched according to birth year and number of offspring.

For *BRIP1*, one of the 15 genes tested for excess miscarriage, the stop gained variant p.Arg798Ter (MAF_Iceland_ = 0.21%), and the frameshift variant p.Leu680PhefsTer9 (MAF_Iceland_ = 0.46%) account for the large majority of pLOF carriers. The p.Leu680PhefsTer9 is absent from most population databases^13^ and is likely an Icelandic founder mutation. Homozygous and compound heterozygous mutations in *BRIP1* have been reported as a cause of Fanconi anemia, complementation group J (OMIM:607039). A compound heterozygous genotype consisting of p.Arg798Ter and the missense mutation p.Ala349Pro has been reported in a stillborn fetus at a gestational age of 22 weeks, who was diagnosed with Fanconi anemia complementation group J^28^. Frameshift at the Leu680 position are reported to cause Fanconi anemia (VCV000128166), and p.Leu680PhefsTer9 is associated with a high risk of ovarian cancer in Iceland among heterozygotes^29^. Interestingly, a *BRIP1* compound heterozygous genotype consisting of the p.Arg798Ter stop-gain and p.Leu680PhefsTer9 frameshift variants was deemed causative in a clinical sequencing setting in Iceland in a fetus diagnosed with radial dysplasia in utero.

For c.946-1 G > C in *DHCR7* which has the most prominent homozygous deficit and miscarriage excess in the current study, in a few reported cases, homozygosity leads to either early miscarriage and intrauterine fetal demise or severe Smith-Lemli-Optiz syndrome and death before three months of age^30,31^. Our results confirm a recent observation in the Israeli population of excess miscarriage in carrier couples of the c.946-1 G > C variant in *DHCR7*^31^. As we previously reported, two children of heterozygous couples died in their first year^12^. Importantly, carrier couples were not more likely to experience a miscarriage if one parent was a carrier (OR = 1.04 [95% CI: 0.94–1.15], *P* = 0.45, *N* couples = 2034, *N* miscarriage = 554) (Supplementary Data 19). This indicates that the effect of the c.946-1 G > C variant in *DHCR7* on miscarriage is consistent with a recessive model.

## Discussion

We identified 25 genes with protein-altering variants for which there was a significant deficit of homozygosity in a set of 1.52 million individuals. Nineteen of those involve pLOF variants expected to disrupt the protein and six moderate impact variants (five missense and one splice region). Sequence variants in 12 of the 25 genes, cause Mendelian disease under a recessive mode of inheritance, two under a dominant mode, but variants in the remaining 11 genes have not been reported as disease-causing.

We demonstrate that when comparing the 1239 genes without a homozygous deficit based on geneLOFs to the 19 genes with such a deficit, the latter are more likely to be linked to autosomal recessive disease, to result in embryonic lethality when knocked out in mice, and to be essential for the viability of human cell lines. Interestingly, there is evidence of lethality in animal models of orthologous genes in addition to mice. Mutations in *PNKP*, and *RPAP2* orthologs are linked to recessive lethality in the OMIA database (Online Mendelian Inheritance in Animals)^32^ in purebred cattle and pig populations, respectively. A splice acceptor variant in *RPAP2* with a carrier frequency of 21% in a purebred cattle population shows a complete homozygous deficit due to early embryonic lethality^33^. A missense variant p.Gln96Arg in *PNKP* with a carrier frequency of 4.7% has a complete homozygous deficit in purebred pig populations^34^. In addition, inactivation of *ATP5PB, PMM2*, and *WARS2* orthologs causes embryonic lethality in zebrafish, fruit-flies, and worm^35–39^ (Supplementary Data 20).

Thirteen genes with a strong deficit of homozygosity are most likely crucial in early development, based on the fact that they are essential for the growth of human cell lines or lethality if knocked out in mice (Supplementary Data 21). Importantly, eight of those genes are not currently linked to Mendelian disease in humans^15^. If a mutation in a gene is not known to cause human disease but exhibits a strong deficit of homozygosity it can, in theory, be due to any event from early embryonic selection to sickness in adults that prevents them from participation in research. If variants with a strong deficit of homozygosity led to disease after birth then they could have been recognized in OMIM already. Consequently, we postulate that a strong deficit of homozygosity in these unreported genes confer their effect early in development. Among the eight genes not currently linked to Mendelian disease in humans, the p.Ile233Arg variant in the mitoribosomal protein^40^ MRPS30 has the most prominent deficit of 48 homozygotes. This variant is present in all of the European populations considered with an allelic frequency ranging from 0.3% to 1%, indicating that it is ancient in origin. Assuming a generation time of 25 years, the estimated age of the G allele of rs72756207 resulting in the Ile233Arg missense variation of MRPS30 is estimated to be 16,000 years (637 generations) (95% CI: 380–923 generations, 9500–23,000 years)^41^. In comparison, the homozygous deficit observed for p.Ile233Arg in MRPS30 is on par with p.Arg141His in PMM2 which is the most frequently reported pathogenic variant for congenital disorder of glycosylation^42–44^(OMIM:601785.0001, ClinVar Variation ID:7706) with an allelic frequency ranging from 0.5% to 0.7%. *MRPS30* is essential for the growth of human cell lines but a knockout in mice has not been reported. Further studies are required to understand the biological impact of p.Ile233Arg in MRPS30.

Known disease-causing sequence variants with an established loss-of-function effect that have a homozygous deficit in our data (i.e. *DHCR7*, *GBE1*, *GLE1*, *PMM2, PNKP*, and *TSFM*) have almost exclusively been reported in compound heterozygous cases in combination with a hypomorphic allele (resulting in only partial loss-of-function as cataloged in OMIM and ClinVar). This suggests that the variants that we describe are at least partial loss-of-function variants and that some minimum level of activity is required for successful embryonic development. By assessing RNA and protein levels in heterozygous carriers we are able to provide experimental validation of the effect of variants in ten of the genes with a strong deficit of homozygosity. This includes six variants not reported as disease-causing in *ATP5PB*, *CCDC59*, *GTF2H3*, *MVD*, *PUM3*, and *RPAP2* in addition to the abovementioned known disease-causing loss-of-function variants in *DHCR7*, *GBE1*, *PMM2*, and *PNKP*.

In addition to the genes for which we observe a significant deficit, the results presented here also include information about the genes that do not reach significance (Supplementary Data 4, 6, 17, 18, and 21). Whereas we determined the cutoff for the significance of deficit at five or more expected homozygotes of pLOF variants, we noted that the group of genes with one to five expected homozygotes and a deficit, is also enriched for recessive Mendelian disease, lethal when knocked-out in mice and essential in cell lines. This information, despite not reaching significance, may help in the interpretation of clinical sequencing and study of Mendelian diseases, including cases of neuropsychiatric disease as previously demonstrated^45^.

In addition to detecting genes with a deficit of homozygotes, we identified 2671 genes with observed homozygotes for pLOFs, most of which involve two or more individuals (1722/2671 = 66.3%) in the set of 1.52 M individuals. Some of the annotated pLOF variants where we observe homozygots may not be true loss-of-function variants meaning that true loss-of-function homozygotes could still not be viable. Also, our analysis will only identify deficit of genes that cause loss-of-function homozygotes to be absent from the general population, and the detection of homozygotes for pLOFs suggests that biallelic loss-of-function mutations of these genotypes are not lethal before adult age. However, we cannot exclude the possibility that some of these genotypes would have severe phenotypic effects (Supplementary Discussion).

The approach employed in this study allows for the detection of genes with a strong deficit of homozygosity, resulting from the impact of homozygous genotypes on early stages of development. Homozygous deficit variants that have previously been unnoticed can now be detected in data sets derived from a combination of whole-genome sequencing and genotype imputation into large population sets. The overall burden of homozygous deficit variants at the population level is notable, where the combined deficit of significant protein-altering variants amounts to 444 individuals who were not born in our combined population set of 1.52 million (~3/10,000 individuals). We have identified recessive alleles that decrease reproductive success in the general population. Furthermore, they shed light on the genetic causes of pregnancy loss and add to the understanding of the function of genes that are essential for successful development of a human.

## Methods

### Study samples and ethics declarations

For Iceland, this study is based on whole-genome sequence data from the white blood cells of 49,708 Icelanders participating in various disease projects at deCODE Genetics^14^. In addition, a total of 155,250 Icelanders have been genotyped using Illumina SNP chips. All participating individuals who donated blood or buccal tissue samples, or their guardians, provided written informed consent. All sample identifiers were encrypted in accordance with the regulations of the Icelandic Data Protection Authority. Personal identities of the participants and biological samples were encrypted by a third-party system approved and monitored by the Icelandic Data Protection Authority. The study was approved by the Data Protection Authority (ref. 2013030423/ÞS/−, with amendments) and the National Bioethics Committee (ref. VSN-19-023, VSNb2019010015/03.01), which also reviewed and approved the protocol, methodology, and all documents presented to the participants. All methods were performed in accordance with the relevant guidelines and regulations.

The UK Biobank resource is a large-scale prospective study that includes data from 500,000 volunteer participants who were recruited between the age of 40–69 years in 2006–2011 across the United Kingdom (https://www.ukbiobank.ac.uk/). Various health records and health-related information is available and regularly updated for these 500,000 participants. The UK Biobank phenotype and genotype data were collected following an informed consent and the study is overseen by The North West Research Ethics Committee that reviewed and approved UK Biobanks scientific protocol and operational procedures (REC Reference Number: 06/MRE08/65).

Danish samples were obtained through collaboration with the Danish Blood Donor Study (DBDS) and the Copenhagen Hospital Biobank (CHB). The Danish Blood Donor Study (DBDS) GWAS study is a large prospective cohort study of ~110,000 blood donors across Denmark^46^. The Danish Data Protection Agency (P-2019-99) and the Danish National Committee on Health Research Ethics (NVK-1700704) approved the studies under which genetic data on DBDS participants were obtained. CHB is a research sample repository, which contains left-over samples obtained from diagnostic procedures on hospitalized and outpatient patients in the Danish Capital Region hospitals^47,48^. Genotypic data from the CHB were included as part of the study.

Norwegian genotype data were obtained from both hospital and population-based samples. Clinical samples included data from the DemGene and TOP studies which consist of case control samples of neuropsychiatric disorders. Written informed consent was obtained, and the Regional Committee for Medical and Health Research Ethics (REC) South East (#2009/2485) and Mid Norway (#2014/631) approved the studies. Population-based samples included data from the Norwegian Mother, Father and Child cohort study (Mor og Barn; MoBa) and the Hordaland Health Study (HUSK). MoBa is a population-based pregnancy cohort study conducted by the Norwegian Institute of Public Health. Participants were recruited from all over Norway from 1999–2008. The women provided consent to participation in 41% of the pregnancies. The cohort includes approximately 114,500 children, 95,200 mothers and 75,200 fathers. Blood samples were obtained from both parents during pregnancy and from mothers and children (umbilical cord) at birth. For a more detailed description of the MoBa sample see Magnus et al.^49,50^. The current study included genotype data from 168,000 mothers, fathers and offspring. The establishment of MoBa and initial data collection was based on a license from the Norwegian Data Protection Agency and approval from the REC. The MoBa cohort is currently regulated by the Norwegian Health Registry Act. Written informed consent was obtained from all mothers and fathers participating in MoBa. The current study was approved by REC South East (#2016/1226). MoBa is supported by the Norwegian Ministry of Health and Care Services and the Ministry of Education and Research. We are grateful to all the participating families in Norway who take part in this on-going cohort study. The HUSK Study is a community-based prospective study conducted in Hordaland County in Norway (http://husk.b.uib.no). The project was approved by REC (Western Norway 2018/915), and written informed consent was obtained from all participants. Genotypic data was provided by the HARVEST collaboration (supported by the Research Council of Norway (RCN) (#229624), the NORMENT Centre (RCN #223273) South East Norway Health Authorities and Stiftelsen Kristian Gerhard Jebsen; in collaboration with deCODE Genetics, and the Center for Diabetes Research at the University of Bergen (funded by the ERC AdG project SELECTionPREDISPOSED, Stiftelsen Kristian Gerhard Jebsen, Trond Mohn Foundation, the RCN, the Novo Nordisk Foundation, the University of Bergen, and the Western Norway Health Authorities).

Genotypic data from Sweden was primarily retrieved from disease-specific population-based case-control studies on chronic inflammatory diseases, including studies on multiple sclerosis (EIMS)^51,52^ (04/252 1-4 & 2019-00639) and STOPMS2 (2009/2107-31/2 & 2020-0712), approved by National Ethical review board, GEMS^53^, IMSE^54^, and IMSE2 (2011/641-31/4), STOPMS^55^ (02-548), and COMBATMS^56^ (2017/32-31/4) approved by The Stockholm Regional Ethical Review Board, and rheumatoid arthritis (EIRA, Umea)^57,58^. The original rheumatoid arthritis studies were approved by the Swedish Ethical Review Authority and all data have been de-identified prior to analyses. Furthermore, genotypic data from the Swedish National Myeloma Biobank^59,60^ (Swedish Ethical Review Authority; Dnr 2019-06386), Skåne University Hospital, Lund, and from Swedish blood donors and primary care patients aged 18 to 71 years from Skane county^61^ (Lund University Ethics Review Board; Dnr 2018/2) were also included. The original studies were approved by the Lund University Ethical Review Board, and all data have been de-identified prior to analyses.

The Finnish data on genotype counts were obtained from the FinnGen project (https://www.finngen.fi/en), which gathers samples and phenotype data from a nationwide network of Finnish biobanks and national health registers. The Coordinating Ethics Committee of the Helsinki and Uusimaa Hospital District evaluated and approved the FinnGen research project which complies with existing legislation (in particular the Biobank Law and the Personal Data Act). The official data controller of the study is the University of Helsinki. The genotype data were imported on May 11th, 2021 from a source available to consortium partners (version 5; http://r5.finngen.fi).

### Genotyping

The 155 K Icelanders had 27.2 million imputed sequence variants discovered through whole-genome sequencing of 50 K Icelanders^21^. Our approach to WGS, genotyping, long-range phasing, and imputation of a substantial fraction of the Icelandic population has been described in detail in previous publications^14,62^. In brief here for the benefit of the readers, 56,959 Icelanders have been WGS using standard TrueSeq methodology (Illumina), to a median depth of 37X, and genotyped with Illumina microarrays (chip-genotyped). An additional 96,095 Icelanders have been chip-genotyped and not WGS. Genotypes of sequence variants identified through sequencing (SNPs and indels) have been imputed into all chip-typed Icelanders, resulting in a set of 153,054 chip-genotyped and imputed Icelanders. We report carrier status among imputed samples if genotype probability exceeds 0.9. Samples and variants with less than 98% yield were excluded. For the purpose of this study, individuals with either one or both parents of foreign ancestry, and individuals WGS for the purpose of clinical diagnostics were removed from the set.

The 432 K participants in the UK Biobank in this study had 57.7 million imputed sequence variants discovered through whole-genome sequencing of 150,119 individuals from UKB^63^. We report carrier status among imputed samples if genotype probability exceeds 0.9. Samples and variants with less than 98% yield were excluded. For the purpose of this study, our analysis was limited to individuals with British-Irish ancestry (XBI) as defined elsewhere^63^.

Samples from Denmark, Norway, and Sweden were genotyped using Illumina Global Screening Array chips and long-range phased together with other genotyped samples from North-western Europe using Eagle2^64^. For the purpose of this study, individuals of non-European ancestry were removed from the set based on principal component analysis based on genotypes in the set of North-western Europeans.

We report carrier status among imputed samples if genotype probability exceeds 0.9. Samples and variants with less than 98% yield were excluded. A haplotype reference panel was prepared in the same manner as for the Icelandic and UK data^14,65^ by phasing whole-genome sequence genotypes of 15,576 individuals from Scandinavia, the Netherlands, and Ireland using the phased chip data. Graphtyper was used to call the genotypes which were subsequently imputed into the phased chip data.

Whole-genome sequencing, chip-typing, quality control, long-range phasing, and imputation from which the data for this analysis were generated was performed at deCODE genetics.

A custom-made FinnGen ThermoFisher Axiom array (>650,000 SNPs) was used to genotype ~177,000 FinnGen samples at Thermo Fisher genotyping service facility in San Diego. Genotype calls were made with AxiomGT1 algorithm. Individuals with ambiguous gender, high genotype missingness (>5%), excess heterozygosity (±4 SD), and non-Finnish ancestry were excluded. Variants with high missingness (>2%), low Hardy-Weinberg equilibrium (HWE) (<1 × 10^−6^), and minor allele count (<3) were excluded. High coverage (25–30×) WGS data was used to develop the Finnish population-specific SISu v3 imputation reference panel with Beagle 4.1. More than 16 million variants have been imputed (https://finngen.gitbook.io/documentation/methods/genotype-imputation).

We manually assessed BAM files of different regions of variants with homozygous deficit, with particular interest in those with indels. These included the AGK chr7:141649323 TAAC duplication, the MVD chr16:88663006 C to T substitution, the CCDC59 chr12:82354490 TTACTTGT deletion, and the RPAP2 chr1:92333464 GAGTA deletion (Supplementary Figs. 8–11). We examined the BAM files of more than 20 individuals of each genotype, including heterozygotes and non-carriers, to confirm that the data in the BAM files corresponded to the reported genotypes in all cases. The reference allele was observed to have multiple copies in heterozygotes in all cases.

### Imputation

Samples chip-typed and whole-genome sequenced at deCODE genetics from Denmark, Iceland, UK, Norway, and Sweden were long-range phased^65^, and the variants identified in the whole-genome sequencing were imputed into the chip-typed individuals, as has been described in detail elsewhere^14,63^. We restrict our analysis to variants that are reliably imputed with leave-one-out r-squared score (L1oR2) score greater than 0.5 and imputation info above 0.9^14,63^. Because our imputations are based on haplotype rather than genotype, we are less likely to encounter artificial deficits in homozygotes as a result of genotyping or imputation errors^14,63^. Importantly, given the two phased haplotypes of each individual, the imputation of the individual’s two haplotypes was performed independently which leads to less dependence between the imputed alleles than when genotypes are imputed from genotypic data.

For samples from Finland imputation was done with the population-specific SISu v3 reference panel^66^ with Beagle 4.1 (version 08Jun17.d8b) as described in the following protocol: dx.doi.org/10.17504/protocols.io.nmndc5e. We restrict our analysis to variants with INFO score greater than 0.9.

### Identification of a deficit in the number of observed homozygotes

We tested the deficit of observed homozygotes for variants with an expected homozygote count over 0.5. This corresponds to an allelic frequency >0.1% the set of 1.5 million. Given the frequency (p) in a population and assuming random mating, the number of homozygotes is expected to be p^2^ under HWE. The combined expected number of homozygotes in the six populations is the sum of the expected number of homozygotes from each population.

We used Variants Effect Predictor (VEP)^19^ to assess the functional impact of sequence variants. We assessed homozygote count for intergenic variants (located in intergenic regions more than 5 kb from a RefSeq annotated genic region), low-impact variants (intronic variants, synonymous variants, and 3’UTR/5’UTR variants within 5 of an exon), moderate-impact variants (missense, inframe indel, splice region), and high impact variants (a.k.a. predicted loss-of-function variants) (stop-gained, frameshift, essential splice donor and acceptor). We restricted our analysis to autosomal variants that fall within Tier 1 high confidence regions based on Genome in a Bottle consortium (GiaB)^67^, and excluded variants located in segmental duplications, centromeres, telomeres, and low mappability regions that are difficult to map with short-read sequencing technologies^67^.

For each sequence variant, we derived an estimate of the allele frequency of the variant in each population *i* from the genotyped individuals as

$${\hat{p}}_{i}=\frac{{Expected}\,{number}\,{of}\,{carrier}\,{haplotypes}\,{in}\,{population}\,i}{2{n}_{i}}$$,

where n_*i*_ denotes the number of individuals in population *i* that were genotyped for the variant. Since here we are primarily interested in rare sequence variants, the estimated allele frequency is driven by the number of observed non-carriers and heterozygotes, and only slightly affected by the number of homozygotes. Under HWE, ${n}_{i}{\hat{{p}_{i}}}^{2}$ is the expected number of homozygotes within population i. Under HWE within each population, the expected total number homozygotes is then ${\lambda={\varSigma }_{i}{n}}_{i}{\hat{{p}_{i}}}^{2}$. We considered a variant to have a strong deficit of homozygosity if the observed number of homozygotes was 10% or less of the expected number of homozygotes under HWE, i.e. if the observed number of homozygotes was less than 0.1λ. This criterion was used instead of 0% to allow for some deviation from a total deficit as used in animal models^17,18^.

Since we are focusing on rare variants, the observed number of homozygotes then approximately follows a Poisson distribution with mean λ. This allows us to calculate a *P*-value for deviation from HWE which can then be corrected using Bonferroni correction to obtain a significance threshold for each set of variants. However, deviations from random mating within each population tend to increase the number of homozygotes. We therefore used the intergenic variants, which are the sequence variants with the lowest predicted functional impact, to estimate the probability that a sequence variant has a strong deficit of homozygosity in the absence of HWE. We grouped variants based on their expected number of homozygotes under HWE and calculated the fraction of variants with a strong deficit of homozygosity. The groupings of expected number of homozygosity we used were: [0.5–1), [1, 2), [2, 3), [3, 5), [5, 8), [8, 13), [13, 250), [250, ∞). Within one of these ranges of expected number of homozygotes under HWE, let f_intergenic and f_pav denote the fraction of variants with a strong deficit of homozygosity among intergenic sequence variants, and protein-altering sequence variants, respectively. A false discovery rate (FDR) was estimated by dividing the fraction of intergenic sequence variants with a strong deficit of homozygosity by the fraction of protein-altering sequence variants with a strong deficit of homozygosity:

$$\,{FDR}\,=\frac{f{{{{{\rm{\_}}}}}}{intergenic}}{f{{{{{\rm{\_}}}}}}{pav}}$$

Using the fraction of variants at deficits of homozygosity among intergenic variants as a reference does address the issue of artificial deficit of homozygotes caused by genotyping or imputation artifacts since imputation artifacts should not preferentially affect protein-altering variants over intergenic variants. FDR confidence intervals were calculated using the ad-hoc approximate-estimate CI (AECI) method, which estimates a confidence interval for the ratio of two independent Poisson rates^68^.

To account for hitchhiking effects due to linked selection, we excluded highly correlated variants between impact classes and additionally defined sets of intergenic variants with different exclusion regions outside of RefSeq annotated genes to calibrate the FDR. Specifically, moderate-impact variants highly correlated (R^2^ > 0.8) with high-impact variants were removed from the moderate-impact class, low-impact variants highly correlated with moderate or high-impact variants were removed from the low-impact class, and intergenic variants highly correlated with moderate, high, or low-impact variants were removed from the intergenic class. Additionally, we defined sets of intergenic variants located 5 kb, 50 kb, 100 kb, 250 kb, and 500 kb outside of annotated genic regions (Supplementary Data 22). There were no substantial fluctuations in the FDR as a result of the choice of intergenic variant sets (Supplementary Fig. 7). For further analysis we used intergenic variants located 5 kb outside of annotated genic regions which is the definition used by VEP^19^. As the number of intergenic variants 500 kb outside annotated genic regions is lower than the number of low-impact variants (875,258 compared to 877,296), it is likely that an exclusion region of such a size is excessive (Supplementary Data 22).

### geneLOFs

We collapsed rare and low frequency (<2% minor allele frequency) predicted loss-of-function variants by autosomal genes for the geneLOF tests^69,70^. Assuming that all loss-of-function variants have the same phenotypic effect, collapsing genotypes across the variants maximizes the power to detect association^71^. We excluded sequence variants deemed as low-confidence by the LoFtee (Loss-Of-Function Transcript Effect Estimator) algorithm, and variants labeled “likely not LoF” and “not LoF” after manual curation of pLOF variants that have passed all LoFtee filters^13^. Loss-of-function burden tests have used frequency thresholds from 0.1% to 5% MAF^72,73^ to attenuate the probability of false-positive loss-of-function variants in the burden test. Here, we filtered on loss-of-function MAF below 2% because pathogenic variants can be of higher allele frequencies in populations with founder effects, such as in Iceland and Finland^74–76^.

### Gene expression analysis

We sequenced RNA from whole blood from 17,848 Icelanders, described in detail elsewhere^77^. We computed gene expression based on personalized transcript abundances using kallisto^78^. We quantile normalized the gene expression estimates and adjusted for measurements of sequencing artifacts, demographic variables, blood composition, and hidden covariates^79^. We then tested for association with sequence variants.

We used the SomaLogic® SOMAscan proteomics assay to measure protein levels in plasma^21^. The assay scanned 4907 aptamers that measure 4719 proteins in samples from 35,559 Icelanders with genetic information available at deCODE genetics. We quantile standardized the plasma protein levels and adjusted for year of birth, sex, and year of sample collection (2000–2019). We performed a proteome-wide association study and evaluated whether sequence variants associated with protein levels (pQTL).

### Miscarriage among carrier couples

We identified couples where both partners carry variants with a strong deficit of homozygosity in a heterozygous state. In each pregnancy, these couples have a one-in-four chance of transmitting two copies of the variant with a strong deficit of homozygotes. We looked for records of miscarriage among 61,848 genotyped couples from Iceland where the female partner completed a pregnancy history questionnaire at the Cancer Detection Clinic of the Icelandic Cancer Society, carried out in connection with routine screening for cancers of the cervix and breast between 1964 and 1994 (Supplementary Data 23). Participants were asked if they had experienced a miscarriage, and if so, how many times. Differences in miscarriage risk between carrier couples (carrier mother + carrier father, and where one partner is a carrier) versus control couples (non-carrier mother + non-carrier father) were evaluated using Fisher’s exact test. In this study, we assess excess miscarriage both in terms of the number of mothers experiencing at least one miscarriage, and the number of pregnancies resulting in miscarriage between mothers from carrier couples and control couples. Non-carrier control couples were randomly drawn from the group of 61,848 genotyped couples from Iceland where the female partner answered a routine pregnancy history questionnaire and matched on age and number of pregnancies (1:100 nearest neighbor matching with replacement).

### Gene set over-representation analysis

We performed a gene over-representation analysis using three sets of data: (1) genes harboring variants reported to cause recessive Mendelian disease, (2) genes essential for the growth of human cell lines identified through genome-wide screens, and (3) orthologous mouse genes known to affect viability. Gene set over-representation was estimated by a two-sided Fisher exact test. As the unit of the test is the gene, we used the 1258 geneLOFs with five or more expected homozygotes in the meta-analysis of all 1.52 million individuals.

1. Information on the mode of inheritance of Mendelian disease and linked genes was extracted from the Inheritance subontology of The Human Phenotype Ontology (HPO)^80^ (http://purl.obolibrary.org/obo/hp/hpoa/phenotype.hpoa) (see Supplementary Data 14).
2. Data on genes essential for the growth of human cell lines were derived from genome-wide screens were downloaded from Project Achilles^81,82^ website (https://depmap.org/portal/download). A unified list of of common essential genes from three gene sets was used (Achilles_common_essentials.csv, CRISPR_common_essentials.csv, and Common_essentials.csv) (see Supplementary Data 15).
3. Data on mouse lethal phenotypes was retrieved from the Mouse Genome Informatics (MGI) database (http://www.informatics.jax.org/downloads/reports/MGI_GenePheno.rpt) and the International Mouse Phenotyping Consortium (IMPC). The 15th release of IMPC mouse phenotype data was downloaded from the IMPC ftp site (http://ftp.ebi.ac.uk/pub/databases/impc/all-data-releases/release-15.1/results/viability.csv.gz). A unified list of ‘embryonic lethal’ genes was identified through query of the Mammalian Phenotype Ontology (MP) terms^83^ associated with viability among the joint MGI and IMPC dataset (see Supplementary Data 16).

### Variant age estimation

To estimate the age of selected variants, human genome dating database was used (https://human.genome.dating/snp/rs72756207). Using the reference allele as the ancestral state, age was estimated for the alternate allele, and the generation time was assumed to be 25 years^41^.

### Power analysis

For power analysis, we used a two-sample proportional test. We assumed that the true homozygote frequency in the population was 10% of its expected frequency. We estimated the sample size required to detect a strong deficit of homozygosity with 80% power (significance level = 0.05), as well as the power to detect the effect of a strong deficit of homozygosity on minor allele frequencies between 0 and 1.6%. We used the R function stats::power.prop.test to perform the power analysis (sig.level = 0.05, power = 0.80, p1 = expected frequency of homozygous genotype, p2 = 0.1*p1).

### Reporting summary

Further information on research design is available in the Nature Portfolio Reporting Summary linked to this article.

## Supplementary information

Supplementary Information

Peer Review File

Description of Additional Supplementary Files

Supplementary Data 1-23

Reporting Summary

## Data Availability

All data generated during this study are included in this published article and its supplementary files. Genotype data for protein-altering-variants for the combined set of 1.52 million individuals generated for this study are publicly available and tabulated in Supplementary Data 4, and Supplementary Data 6. Figshare https://figshare.com/s/c498d3df17cb04189135 (2023). This study made use of publicly available datasets. This research has been conducted using the FinnGen resource. The FinnGenn GWAS summary statistics, variant annotation, and genotype counts are publicly accessible following registration at https://www.finngen.fi/en/access_results. To gain access to Finngen data an online form needs to be filled out at https://elomake.helsinki.fi/lomakkeet/102575/lomake.html. Instructions on how to download data from Finngen are then sent per e-mail; This research has been conducted using the UK Biobank Resource under application number 56270. Data from the UK Biobank are available by application to all bona fide researchers in the public interest at https://www.ukbiobank.ac.uk/enable-your-research/apply-for-access. Additional information about registration for access to the data are available at www.ukbiobank.ac.uk/register-apply/. Data access for approved applications requires a data transfer agreement between the researcher’s institution and UK Biobank, the terms of which are available on the UK Biobank website (www.ukbiobank.ac.uk/media/ezrderzw/applicant-mta.pdf); GWAS summary statistics for RNA splicing (sQTL), mRNA levels (eQTL), and protein levels (pQTL) in the Icelandic population, based on RNA sequencing of blood samples from 17,848 individuals and plasma protein levels measured with 4907 aptamers (SOMAscan) in 35,559 individuals^21^ used in this study are publicly accessible following registration at https://www.decode.com/summarydata/ (https://download.decode.is/form/folder/proteomics); Information on the mode of inheritance of Mendelian disease and linked genes was extracted from the Inheritance subontology of The Human Phenotype Ontology (HPO) are freely available at http://purl.obolibrary.org/obo/hp/hpoa/phenotype.hpoa, and tabulated in Supplementary Data 14; Data on genes essential for the growth of human cell lines were derived from genome-wide screens were downloaded from Project Achilles website (22Q2) are freely available at https://depmap.org/portal/download. A unified list of of common essential genes from three gene sets was used (https://depmap.org/portal/download/all/?releasename=DepMap+Public+22Q2&filename=Achilles_common_essentials.csv, https://depmap.org/portal/download/all/?releasename=DepMap+Public+22Q2&filename=CRISPR_common_essentials.csv, and https://depmap.org/portal/download/all/?releasename=DepMap+Public+22Q2&filename=common_essentials.csv), and is tabulated in Supplementary Data 15; Data on mouse lethal phenotypes are freely available and was retrieved from the Mouse Genome Informatics (MGI) database (http://www.informatics.jax.org/downloads/reports/MGI_GenePheno.rpt) and the International Mouse Phenotyping Consortium (IMPC), the 15th release of IMPC mouse phenotype data was downloaded from the IMPC ftp site at http://ftp.ebi.ac.uk/pub/databases/impc/all-data-releases/release-15.1/results/viability.csv.gz. This data is tabulated in Supplementary Data 16.; To estimate the age of selected variants, human genome dating database was used which is freely available (https://human.genome.dating); Data from the OMIA database is freely available. A list of genes for which mutations have been shown to result in Mendelian traits in non‐laboratory animals is available for download at https://www.omia.org/download/causal_mutations/?format=X2.
