# Publisher Correction: Deficit of homozygosity among 1.52 million individuals and genetic causes of recessive lethality

**DOI:** 10.1038/s41467-023-39492-4

**Published:** 2023-07-03

**Authors:** Asmundur Oddsson, Patrick Sulem, Gardar Sveinbjornsson, Gudny A. Arnadottir, Valgerdur Steinthorsdottir, Gisli H. Halldorsson, Bjarni A. Atlason, Gudjon R. Oskarsson, Hannes Helgason, Henriette Svarre Nielsen, David Westergaard, Juha M. Karjalainen, Hildigunnur Katrinardottir, Run Fridriksdottir, Brynjar O. Jensson, Vinicius Tragante, Egil Ferkingstad, Hakon Jonsson, Sigurjon A. Gudjonsson, Doruk Beyter, Kristjan H. S. Moore, Helga B. Thordardottir, Snaedis Kristmundsdottir, Olafur A. Stefansson, Solbritt Rantapää-Dahlqvist, Ida Elken Sonderby, Maria Didriksen, Pernilla Stridh, Jan Haavik, Laufey Tryggvadottir, Oleksandr Frei, G. Bragi Walters, Ingrid Kockum, Henrik Hjalgrim, Thorunn A. Olafsdottir, Geir Selbaek, Mette Nyegaard, Christian Erikstrup, Thorsten Brodersen, Saedis Saevarsdottir, Tomas Olsson, Kaspar Rene Nielsen, Asgeir Haraldsson, Mie Topholm Bruun, Thomas Folkmann Hansen, DBDS Genomic Consortium, Søren Brunak, Kasper Rene Nielsen, Mie Topholm Brun, Hreinn Stefánsson, Unnur Þorsteinsdóttir, Thora Steingrimsdottir, Rikke Louise Jacobsen, Rolv T. Lie, Srdjan Djurovic, Lars Alfredsson, Aitzkoa Lopez de Lapuente Portilla, Soren Brunak, Pall Melsted, Bjarni V. Halldorsson, Jona Saemundsdottir, Olafur Th. Magnusson, Leonid Padyukov, Karina Banasik, Thorunn Rafnar, Johan Askling, Lars Klareskog, Ole Birger Pedersen, Gisli Masson, Alexandra Havdahl, Bjorn Nilsson, Ole A. Andreassen, Mark Daly, Sisse Rye Ostrowski, Ingileif Jonsdottir, Hreinn Stefansson, Hilma Holm, Agnar Helgason, Unnur Thorsteinsdottir, Kari Stefansson, Daniel F. Gudbjartsson

**Affiliations:** 1 deCODE genetics/Amgen, Inc., Reykjavik, Iceland; 2 Faculty of Medicine, School of Health Sciences, University of Iceland, Reykjavik, Iceland; 3 Deptartment of Obstetrics and Gynecology, Copenhagen University Hospital, Hvidovre, Denmark; 4 Department of Clinical Medicine, Faculty of Health, University of Copenhagen, Copenhagen, Denmark; 5 Novo Nordisk Foundation Center for Protein Research, Faculty of Health and Medical Sciences, University of Copenhagen, Copenhagen, Denmark; 6 Methods and Analysis, Statistics Denmark, Copenhagen, Denmark; 7 Institute for Molecular Medicine, Finland, University of Helsinki, Helsinki, Finland; 8 Department of Anthropology, University of Iceland, Reykjavik, Iceland; 9 Department of Public Health and Clinical Medicine, Rheumatology, Umea University, Umea, Sweden; 10 Department of Medical Genetics, Oslo University Hospital and University of Oslo, Oslo, Norway; 11 NORMENT Centre, University of Oslo, Oslo, Norway; 12 KG Jebsen Centre for Neurodevelopmental disorders, University of Oslo, Oslo, Norway; 13 Department of Clinical Immunology, Copenhagen University Hospital, Rigshospitalet, Copenhagen, Denmark; 14 Neuroimmunology Unit, Department of Clinical Neuroscience, Center of Molecular Medicine, Karolinska University Hospital, Karolinska Institutet, Stockholm, Sweden; 15 Department of Biomedicine, University of Bergen, Bergen, Norway; 16 Bergen Center of Brain Plasticity, Division of Psychiatry, Haukeland University Hospital, Bergen, Norway; 17 Icelandic Cancer Registry, Icelandic Cancer Society, Reykjavik, Iceland; 18 Faculty of Medicine, BMC, Laeknagardur, School of Health Sciences, University of Iceland, Reykjavik, Iceland; 19 Division of Mental Health and Addiction, Oslo University Hospital, Oslo, Norway; 20 Centre for Bioinformatics, Department of Informatics, University of Oslo, Oslo, Norway; 21 Danish Cancer Society Research Center, Copenhagen, Denmark; 22 Department of Epidemiology Research, Statens Serum Institut, Copenhagen, Denmark; 23 Norwegian National Centre of Ageing and Health, Vestfold Hospital Trust, Tonsberg, Norway; 24 Department of Geriatric Medicine, Oslo University Hospital, Oslo, Norway; 25 Faculty of Medicine, University of Oslo, Oslo, Norway; 26 Deptartment of Health Science and Technology, Aalborg University, Aalborg, Denmark; 27 Department of Clinical Immunology, Aarhus University Hospital, Aarhus, Denmark; 28 Department of Clinical Medicine, Aarhus University, Aarhus, Denmark; 29 Department of Clinical Immunology, Zealand University Hospital, Koge, Denmark; 30 Department of Clinical Immunology, Aalborg University Hospital, Aalborg, Denmark; 31 Children’s Hospital Iceland, Landspitali University Hospital, Reykjavik, Iceland; 32 Department of Clinical Immunology, Odense University Hospital, Odense, Denmark; 33 Department of Neurology, Copenhagen University Hospital, Rigshospitalet, Glostrup, Denmark; 34 Department of Global Public Health and Primary Care, University of Bergen, Bergen, Norway; 35 Centre for Fertility and Health, Norwegian Institute of Public Health, Oslo, Norway; 36 Institute of Environmental Medicine, Karolinska Institutet, Stockholm, Sweden; 37 Hematology and Transfusion Medicine, Department of Laboratory Medicine, Lund, Sweden; 38 School of Engineering and Natural Sciences, University of Iceland, Reykjavik, Iceland; 39 School of Science and Engineering, Reykjavik University, Reykjavik, Iceland; 40 Department of Medicine, Solna, Karolinska Institutet, Stockholm, Sweden; 41 Department of Mental Disorders, Norwegian Institute of Public Health, Oslo, Norway; 42 Nic Waals Institute, Lovisenberg Diaconal Hospital, Oslo, Norway; 43 PROMENTA Research Center, Department of Psychology, University of Oslo, Oslo, Norway; 44 Analytic and Translational Genetics Unit, Massachusetts General Hospital, Boston, MA USA; 45 Broad Institute of MIT and Harvard, Cambridge, MA USA; 46 Deptartment of Clinical Medicine, Faculty of Health and Medical Sciences, University of Copenhagen, Copenhagen, Denmark

**Keywords:** Genetic predisposition to disease, Disease genetics, Genetics research, Development, Disease genetics

Correction to: *Nature Communications* 10.1038/s41467-023-38951-2, published online 10 June 2023

The original version of the Article omitted to include a statement to indicate Kari Stefansson and Daniel F. Gudbjartsson have jointly supervised the work. This has been corrected in both the PDF and HTML versions of the Article.

