# Supplementary material for: Deficit of homozygosity among 1.52 million individuals and genetic causes of recessive lethality
Source: Nat Commun. 2023 Jun 10;14:3453. doi: 10.1038/s41467-023-38951-2 (PMC10257723; doi:10.1038/s41467-023-38951-2)
Supplement: Supplementary file 1 — Supplementary Information [file 41467_2023_38951_MOESM1_ESM.pdf]

# Supplementary Information

Deficit of homozygosity among 1.5 million individuals and genetic causes of recessive lethality

# Table of contents

**Supplementary Figures** 3
Supplementary Figure 1 4
Supplementary Figure 2 5
Supplementary Figure 3 6
Supplementary Figure 4 6
Supplementary Figure 5 8
Supplementary Figure 6 8
Supplementary Figure 7 10
Supplementary Figure 8 11
Supplementary Figure 9 12
Supplementary Figure 10 13
Supplementary Figure 11 14
Supplementary Figure 12 15
Supplementary Figure 13 16
**Supplementary Discussion** 17
Distribution and medical impact of homozygous deficit variants 17
Early-acting recessive lethal candidate genes 18
Incomplete homozygous deficit 19
Homozygous deficit variants in known Mendelian genes 20
DHCR7 20
MVD 22
PMM2 23
PNKP 23
Homozygous deficit variants in genes not linked to Mendelian disease 25
ATP5PB 25
CCDC59 25
MRPS30 26
BRF2 27
GTF2H3 27
ELOF1 28
RPAP2 28
CASP9 29
MTG2 30
**Supplementary Note 1** 31
Members of the DBDS Genomic Consortium 31
**Supplementary References** 32

# Supplementary Figures

## Supplementary Figure 1

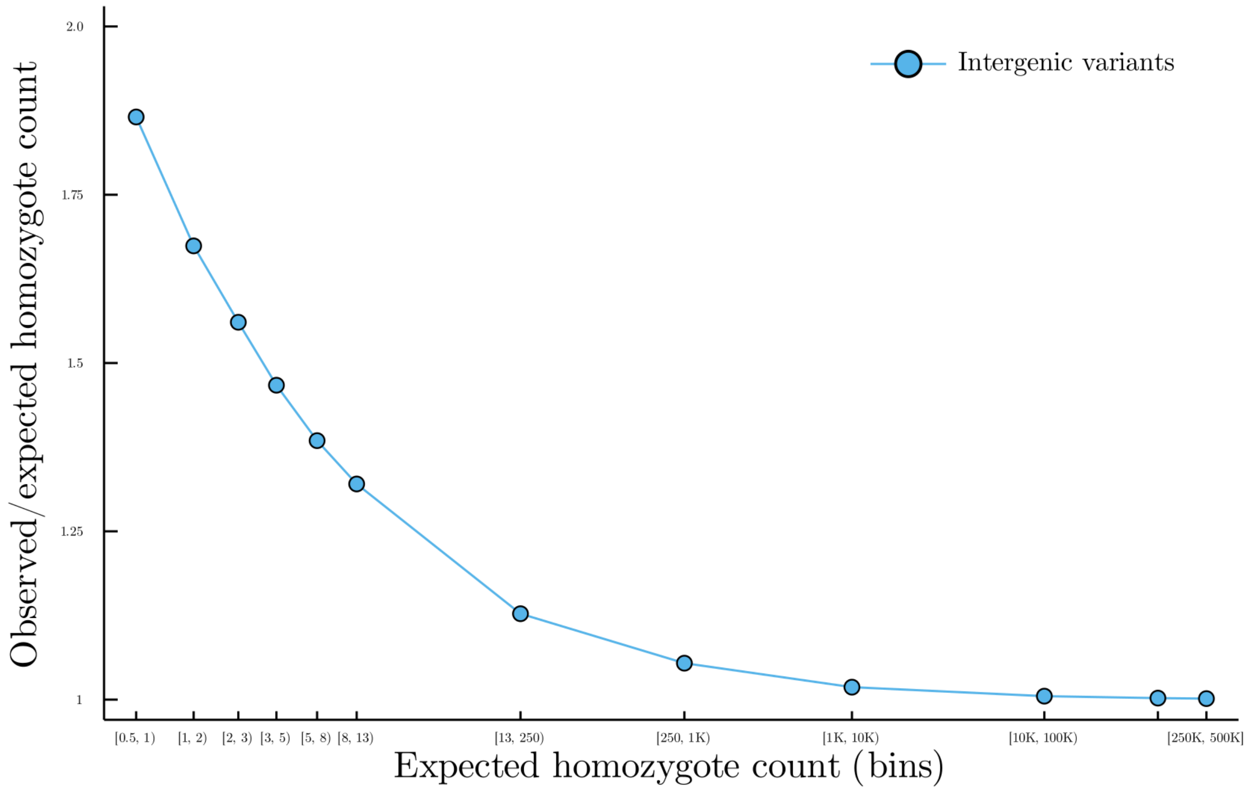


**Supplementary Figure 1.** The ratio of observed to expected minor allele homozygote counts by bins of expected homozygote counts, based on observed heterozygote frequency and the assumption of Hardy-Weinberg equilibrium (HWE) within populations.

## Supplementary Figure 2

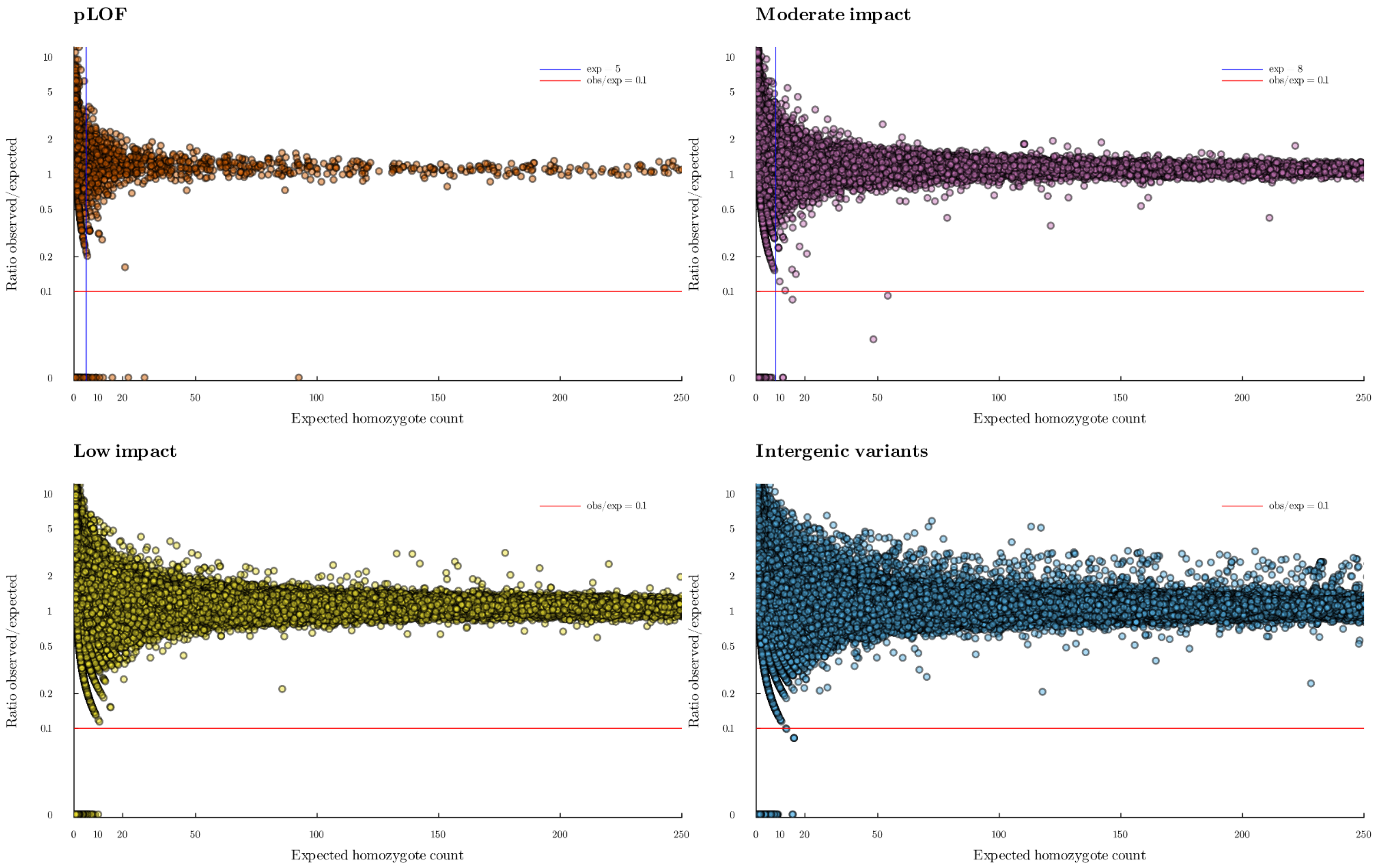


**Supplementary Figure 2.** Observed/expected homozygote count ratio versus frequency for the combined set of 1.52 million individuals from six countries stratified by impact class. The red line indicates a 90% homozygous deficit. The blue line marks 5 and 8 expected homozygote counts for pLOF and moderate impact variants, respectively, corresponding to a false discovery rate cutoff <10%.

## Supplementary Figure 3

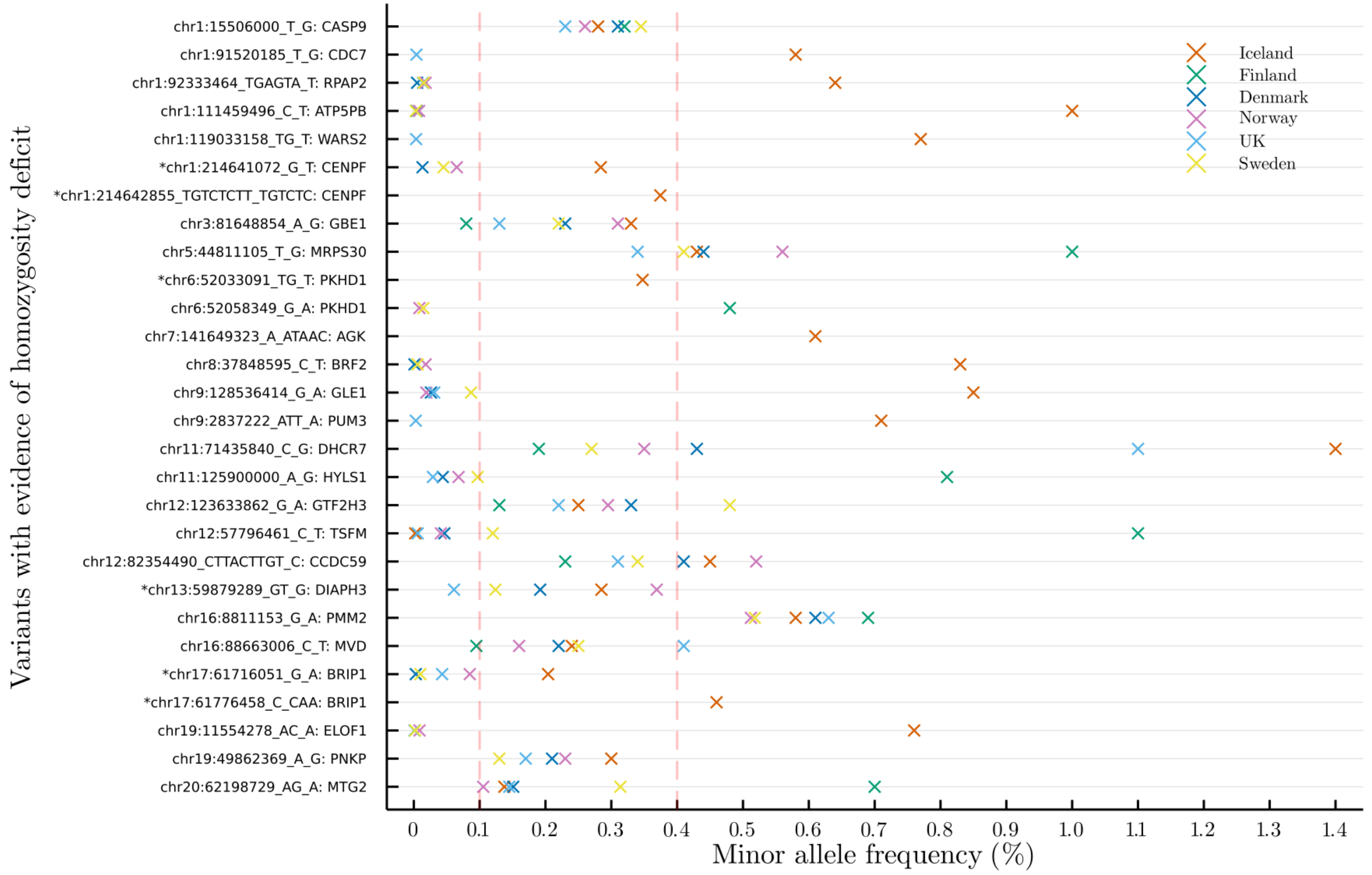


**Supplementary Figure 3.** Comparison of allele frequencies of the 22 single variants with evidence of a homozygosity deficit. Marked with an asterisk are six variants, not reaching significance with single variant testing, included in the geneLOFs of *BRIP1*, *CENPF*, *DIAPH3*, and *PKHD1* with minor allele frequency (MAF) above 0.1% in at least one of the six populations included in the study. Red vertical lines highlight MAF 0.1% and 0.4%.

# Supplementary Figure 4

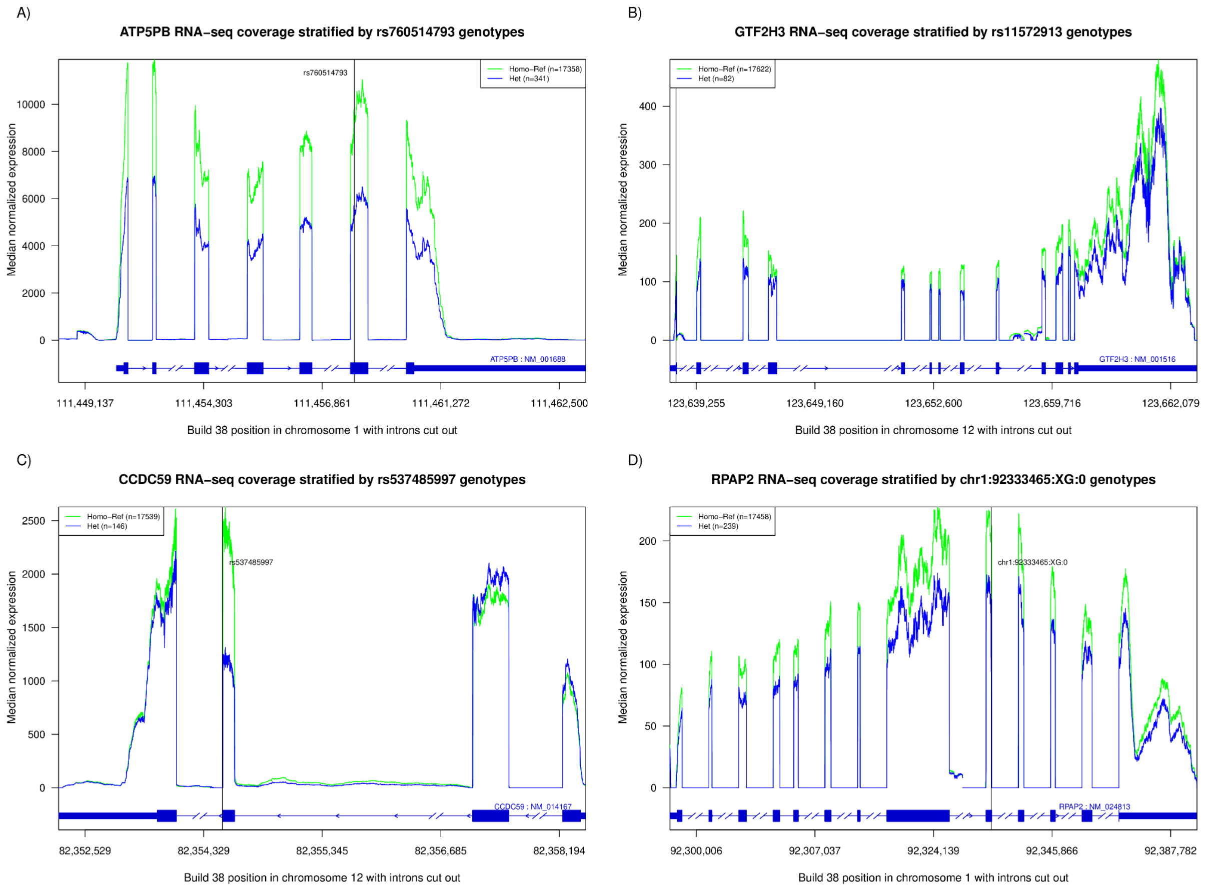


**Supplementary Figure 4.** Effect on RNA expression of homozygous deficit variants in four genes not known to cause Mendelian conditions (*ATP5PB, CCDC59, GTF2H3, and RPAP2*). ). The plots depict the median RNA-sequence coverage for heterozygous (in blue) and noncarriers (in green). A) The stop gained variant p.Arg185Ter (rs760514793) in *ATP5PB* is the top cis-eQTL for *ATP5PB* associating with reduced mRNA levels, consistent with nonsense-mediated decay. B) The start-lost variant rs11572913 in *GTF2H3* associates with homogeneously reduced mRNA levels over all exons, indicating that no expression is initiated from an alternative start site. C) The splice donor variant rs537485997 in *CCDC59* results in exon skipping of exon 3. D) The predicted frameshift variant rs772722639 in *RPAP2* is in LD ($R^2$ = 0.998) with rs772722639 the top cis-eQTL for reduced mRNA levels of RPAP2 and creates an alternative splice acceptor resulting in an inframe deletion of four amino acids.

# Supplementary Figure 5

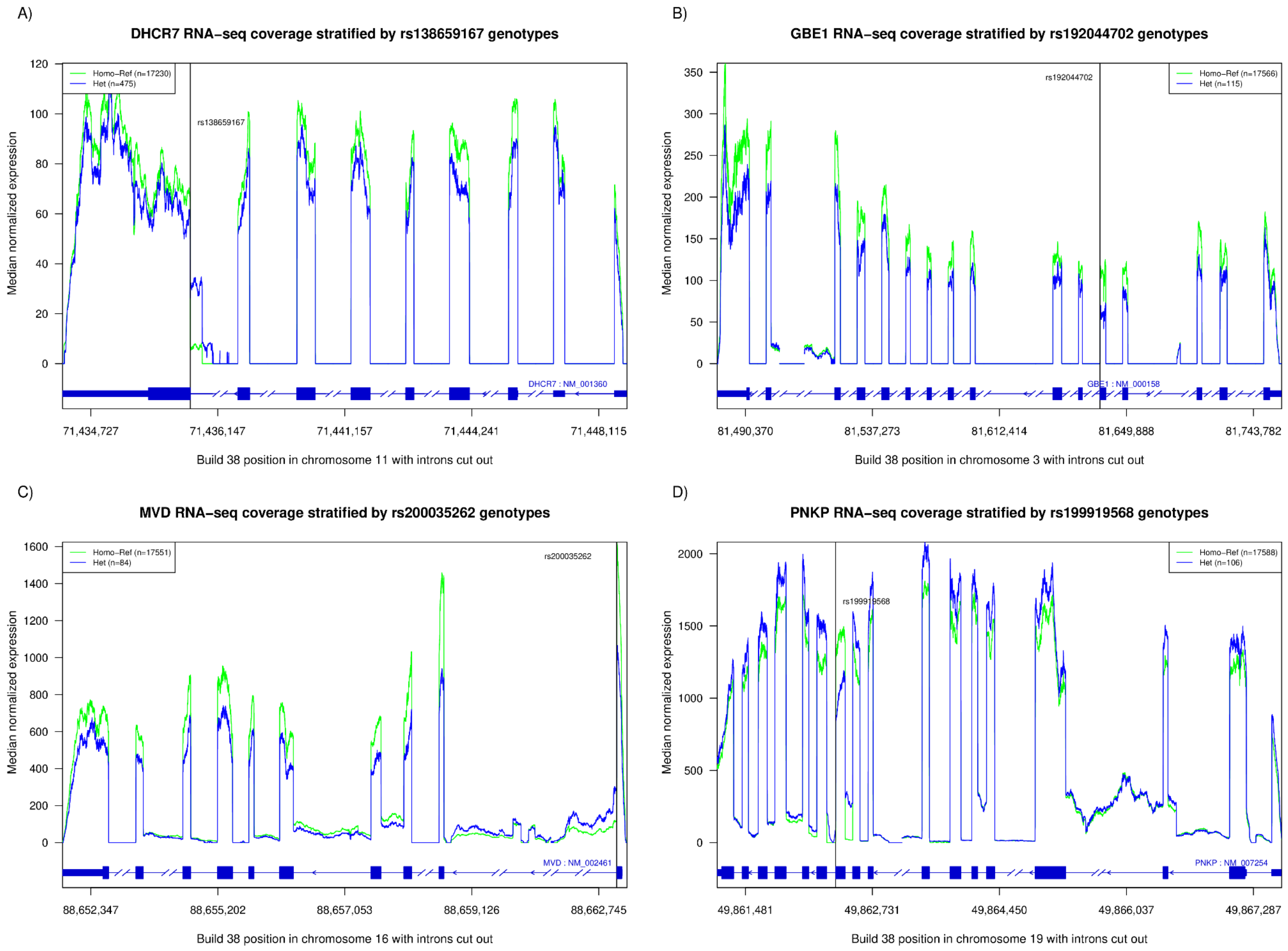


**Supplementary Figure 5.** Effect on RNA expression of homozygous deficit variants in five genes known to cause Mendelian conditions (*DHCR7, GBE1, MVD, and PNKP*). A) The splice donor variant rs138659167 in *DHCR7* creates an alternative splice acceptor in the last exon resulting in a 134 base pair intron retention leading to a frameshift. B) The rs192044702 variant in the *GBE1* results in exon skipping of exon 5. C) The splice region variant c.70+5G>A associated with reduced mRNA levels of MVD in blood. D) The splice donor variant rs199919568 in *PNKP* results in the retained intron between exon 10 and 11 and exon skipping of exon 10.

# Supplementary Figure 6

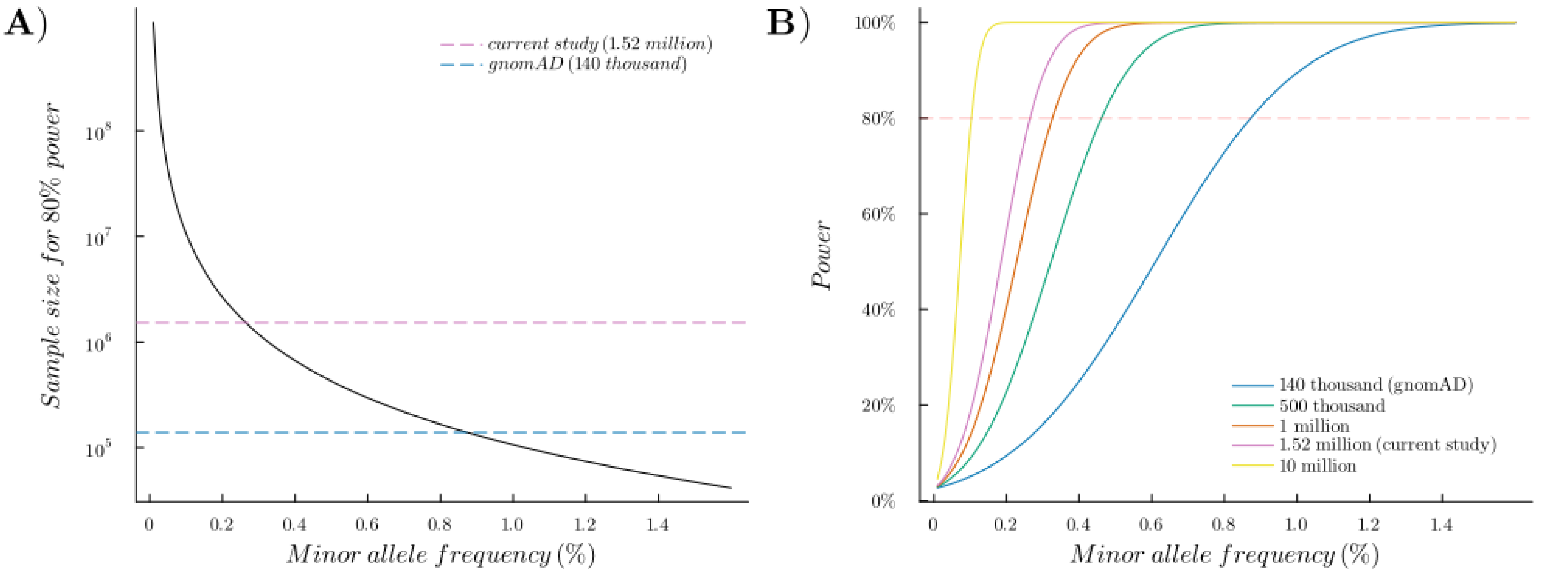


**Supplementary Figure 6.** Power analysis. A) sample size to detect deficit of homozygosity at 80% power at a significance level of 0.05, and B) the power to detect an effect at minor allele frequencies between 0 and 1.6% for various sample sizes (dashed red line indicates a power of 80%).

# Supplementary Figure 7

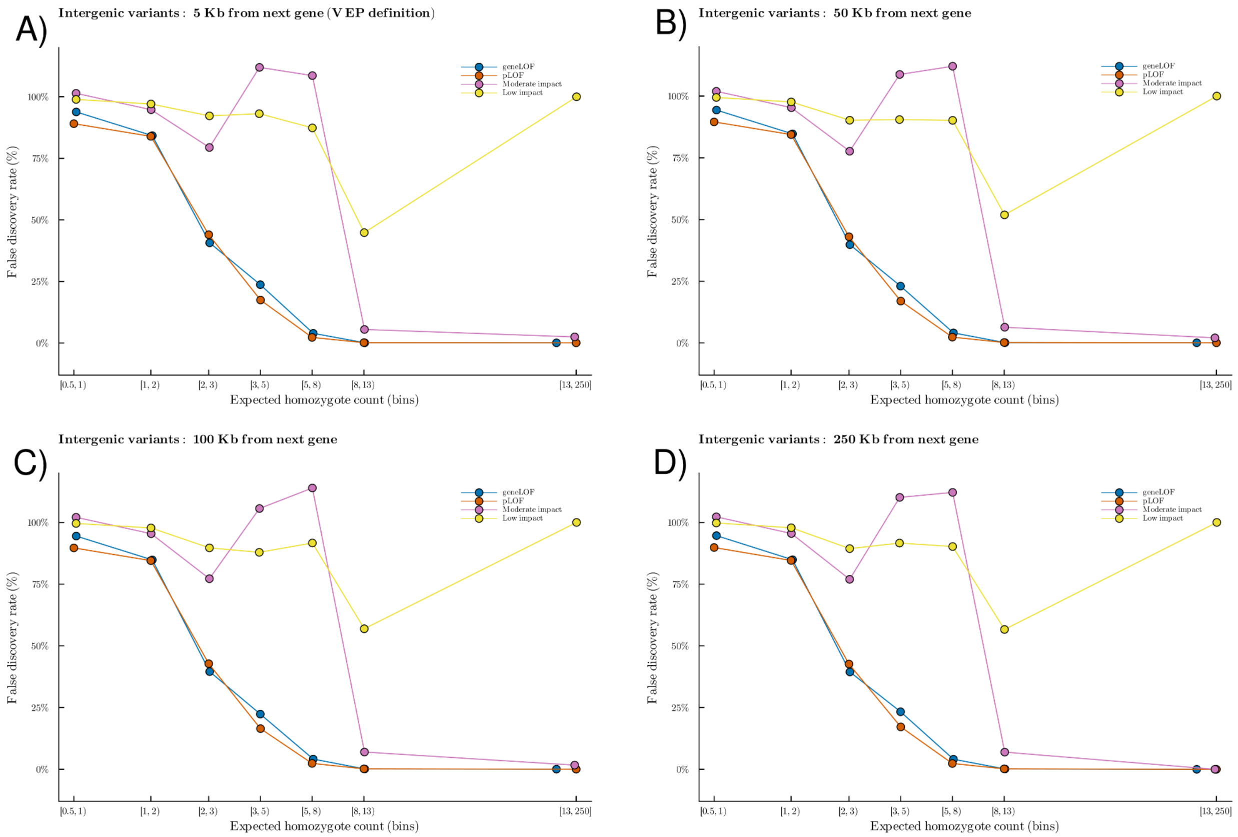


**Supplementary Figure 7:** False discovery rate (FDR) for strong deficit of homozygosity relative to intergenic variants in the combined set of 1.52 million individuals of North-Western European descent (Denmark, Finland, Iceland, Norway, Sweden, and the UK) calculated from different sets of intergenic variants located A) 5 kb (as defined by VEP), B) 50 kb, C) 100 kb, and D) 250 kb outside of RefSeq annotated genic regions.

# Supplementary Figure 8

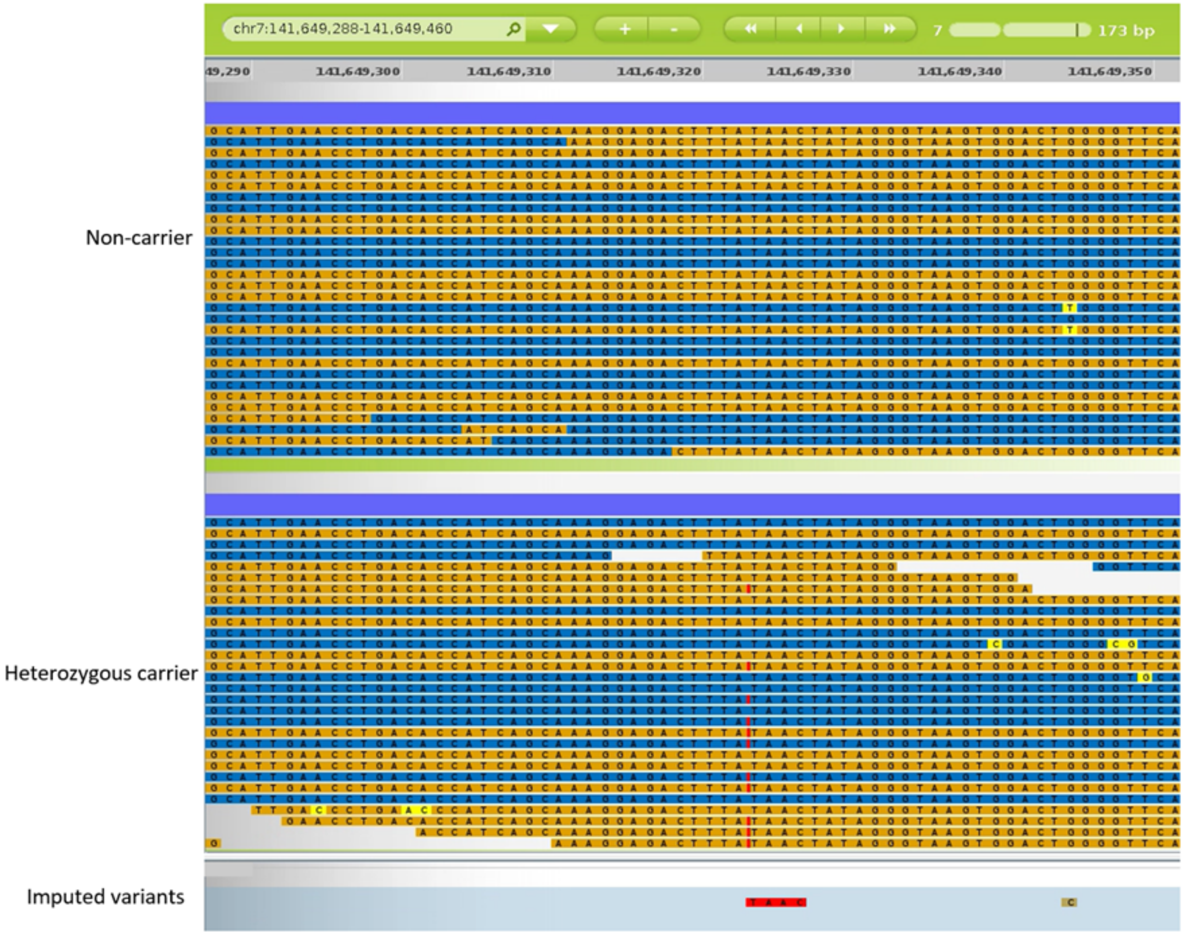


**Supplementary Figure 8.** AGK chr7:141649323 TAAC duplication: The chromosomal position (chr7:141649323) of the TAAC duplication in AGK in a heterozygous carrier (middle track) and a non-carrier (top track). The TAAC duplication is noted in red and marked with an arrow in the figure. The bottom track shows variant calls for all variants that we impute in this region, including the TAAC duplication (Yellow and blue reads are in opposite directions).

# Supplementary Figure 9

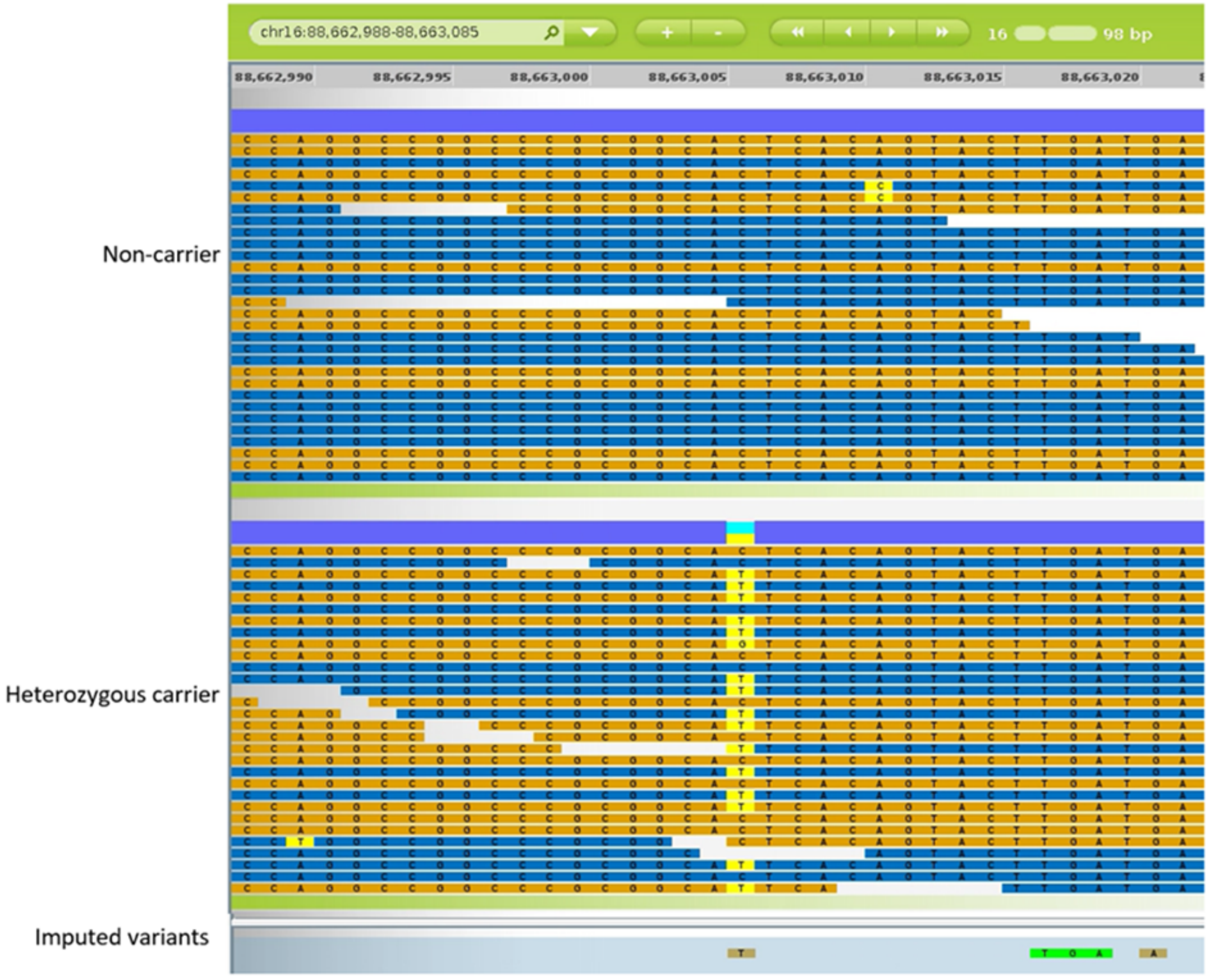


**Supplementary Figure 9.** MVD chr16:88663006 C-T substitution: The chromosomal position (chr16:88663006) of the C-T substitution in MVD in a heterozygous carrier (middle track) and a non-carrier (top track). The C-T substitution is noted in yellow and marked with an arrow. The bottom track shows variant calls for all variants that we impute in this region, including the C-T substitution.

# Supplementary Figure 10

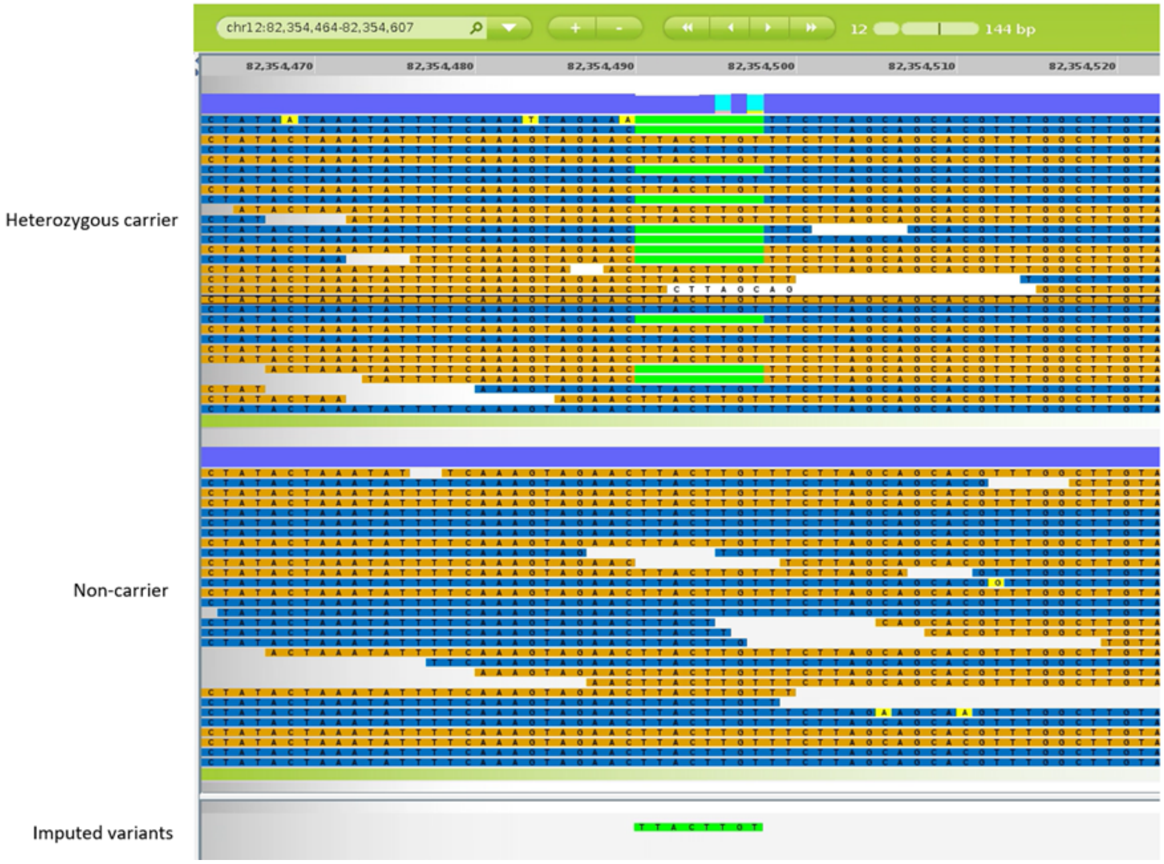


**Supplementary Figure 10.** .CCDC59 chr12:82354490 TTACTTGT deletion: The chromosomal position (chr12:82354490) of the TTACTTGT deletion in CCDC59 in a heterozygous carrier (top track) and a non-carrier (middle track). The TTACTTGT deletion is noted in green and marked with an arrow. The bottom track shows variant calls for all variants that we impute in this region, including the TTACTTGT deletion.

# Supplementary Figure 11

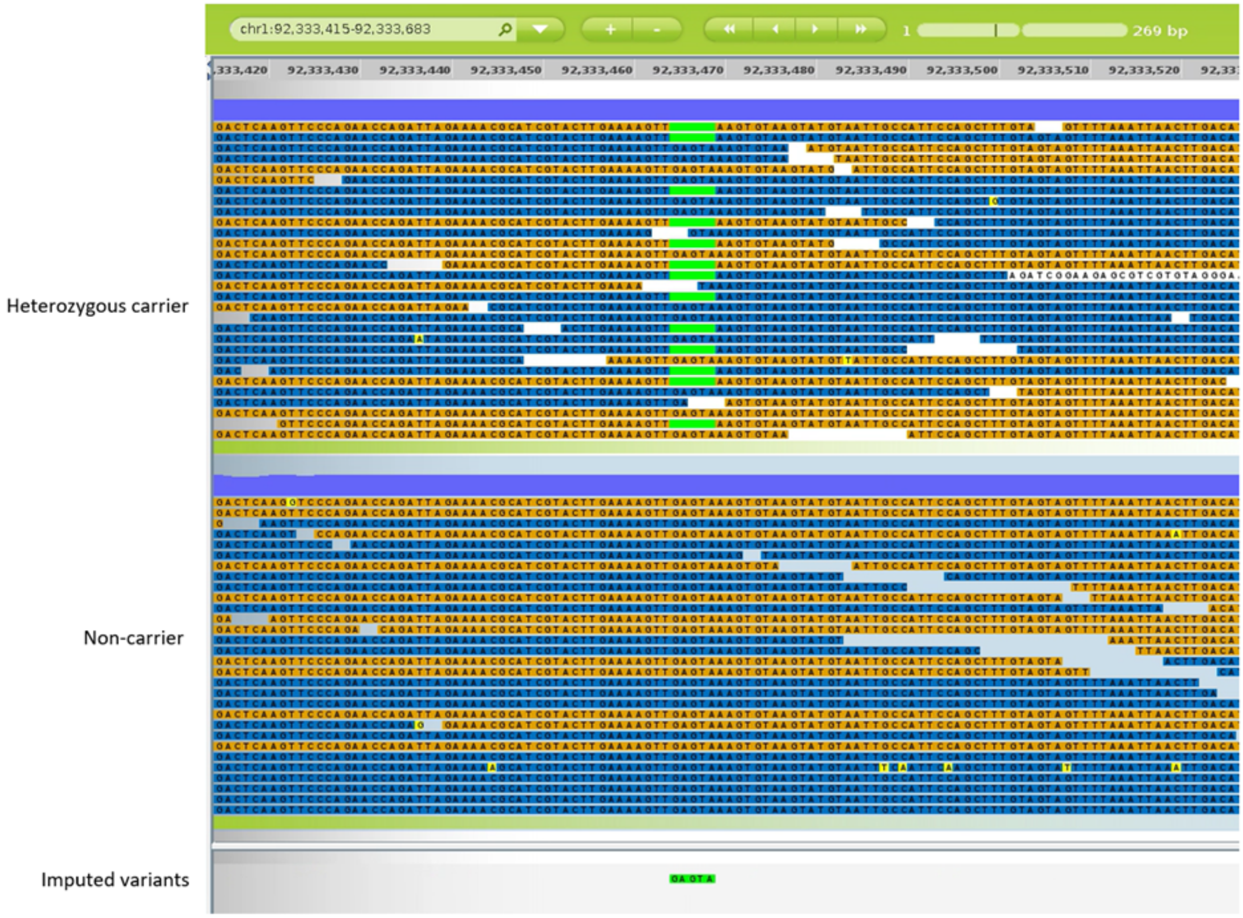


**Supplementary Figure 11.** RPAP2 chr1:92333464 GAGTA deletion**:** The chromosomal position (chr1:92333464) of the GAGTA deletion in RPAP2 in a heterozygous carrier (top track) and a non-carrier (middle track). The GAGTA deletion is noted in green and marked with an arrow. The bottom track shows variant calls for all variants that we impute in this region, including the GAGTA deletion.

# Supplementary Figure 12

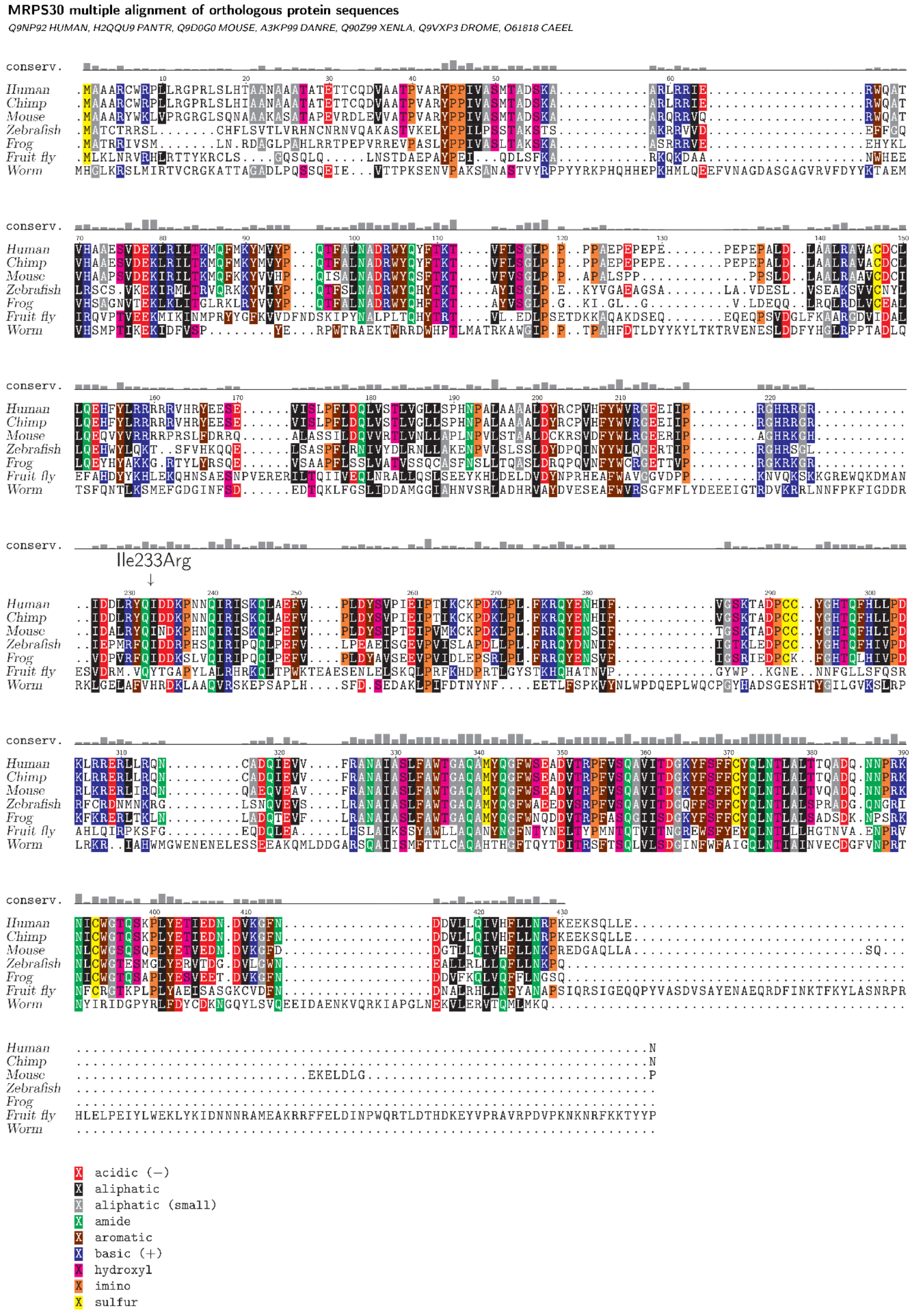


**Supplementary Figure 12.** Multiple alignments of the MRPS30 orthologous protein sequences. The location of the Ile233Arg variant is indicated with a black arrow. Relative conservation (gray bars) and amino-acid chemical properties (see legend) are indicated.

# Supplementary Figure 13

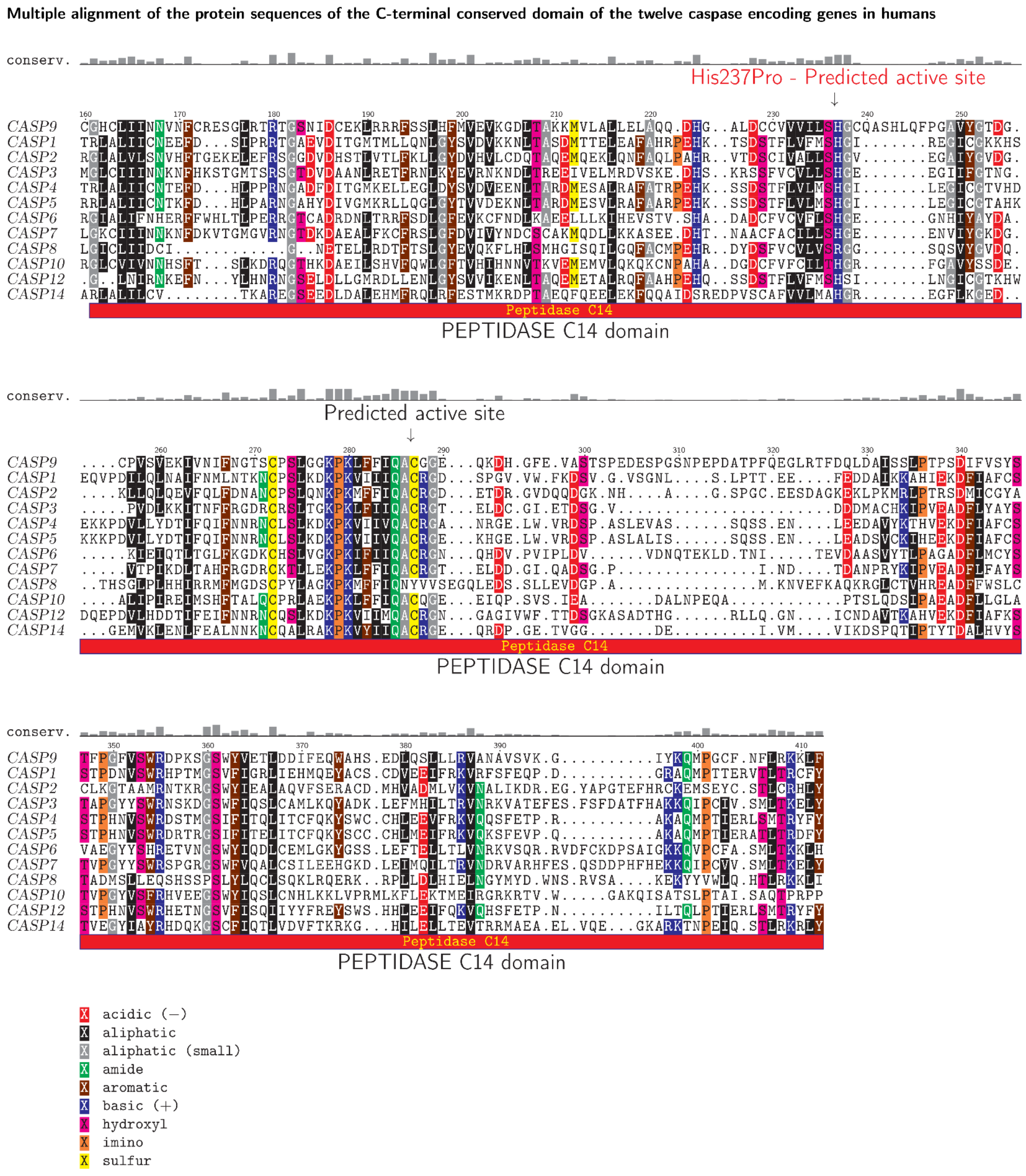


**Supplementary Figure 13.** Multiple alignments of the conserved C-terminal domain of the twelve paralogous caspase encoding genes of the human genome. The location of the His237Pro variant in the predicted active site of CASP9 is indicated with a red arrow. Relative conservation (gray bars) and amino-acid chemical properties (see legend) are indicated.

# Supplementary Discussion

## Distribution and medical impact of homozygous deficit variants

The current study is based on the detection of variants through whole-genome sequencing and imputation into a larger set of chip-typed individuals. The rarest variants tested are common enough to be shared by a sufficient number of sequenced carriers to allow accurate imputation. As we have demonstrated in previous studies, the identification of five sequenced carriers is adequate for accurate imputation[1,2]. The allele frequency distribution of the 22 single variants with evidence of a homozygosity deficit ranges from < 0.001% to 1.4% across the six populations (Table 1, Supplementary Data 4, and 10). Of the 22 variants, 19 have MAF > 0.4% in at least one of the populations. Seven of the variants have MAF > 0.1% in all six populations (Supplementary Figure 3). In contrast, 11 variants are predominantly present in only one of the six populations, in all cases from either Iceland or Finland (MAF > 0.4%, but < 0.1% in the other populations) consistent with a founder effect described in these two populations[2,3]. It is important to bear in mind that the majority of recessive lethal variants are much rarer than those we were able to identify in this study[4]. Thus, we only have statistical power to detect the subset of such variants that have reached the greatest frequencies in at least one or more of the six populations[4].

Of the sixteen pLOF variants with evidence of a homozygosity deficit, all lead to a deficit in a gene-based test (Table 1). Thus, for most geneLOFs, a single pLOF variant accounts for the majority of carriers (Supplementary Data 4 and 6). We did not detect a strong deficit of homozygosity using single variants in three genes detected in the gene test (*BRIP1, CENPF,* and *DIAPH3*). In the case of *BRIP1* and *CENPF*, the geneLOF consisted of two pLOF variants in the Icelandic population that each contributed considerably to the expected number of homozygotes (Supplementary Figure 3). For *DIAPH3*, a single pLOF variant was most common, but the deficit only became significant upon combination with multiple rarer variants (Table 1, and Supplementary Data 4 and 6). For *PKHD1,* the geneLOF was driven by two separate population-specific pLOF variants in Iceland (p.Thr1101AsnfsTer6) and Finland (p.Arg496Ter), with an expected homozygote count of 1.9 and 5.1, respectively (Supplementary Figure 3).

Nine variants with evidence of a homozygosity deficit in the following genes were expected to be carried by one or more homozygotes in at least three populations: *DHCR7*,

*CCDC59*, *GTF2H3*, *GBE1, PNKP, DIAPH3, MRPS30, PMM2,* and *CASP9* (Table 1 and Supplementary Data 8). In contrast, 13 variants were largely population-specific, either to Iceland (*ATP5PB*, *BRF2*, *WARS2*, *ELOF1*, *PUM3*, *RPAP2*, *AGK*, *CDC7*, and *GLE1*) or Finland (*TSFM*, *MTG2*, *PKHD1*, and *HYLS1*) (Table 1 and Supplementary Data 8).

Among the 25 genes harboring variants with a strong deficit of homozygosity, 11 are located in genes that have not been reported to cause a Mendelian condition (Table 1). In ten instances, the variant in question has been reported in genotypes classified as pathogenic or likely pathogenic in the ClinVar database[5] (Table 1 and Supplementary Data 10). Twelve of the 25 genes harbor variants causing recessive Mendelian diseases, of which nine are reported to cause early lethality (*AGK*, *CENPF*, *DHCR7*, *GBE1, GLE1, HYLS1, TSFM*, *PKHD1,* and *PMM2*). Two of the 25 genes harbor variants reported to cause a dominant Mendelian disease (*MVD* and *DIAPH3*).

## Early-acting recessive lethal candidate genes

Based on being lethal when knocked out in mice and essential for growth of human cell lines, ten of the 25 genes with a strong deficit of homozygosity are candidates for harboring variants that lead to early pregnancy loss[6]: *ATP5PB*, *CCDC59, CDC7, GLE1, GTF2H3, WARS2*, *MTG2*, *MVD*, *PNKP*, and *TSFM* (Table 1). Time of embryonic death during mouse development has been assessed for five of these ten genes, and all were found to be lethal during early gestation[7] prior to embryonic day 9.5 (Theiler stage 15) in mice, approximately equivalent to week five in humans (Carnegie stage 11~14)[8]: *CCDC59, CDC7, WARS2*, *MTG2*, and *MVD*. When knocked out in mice, about 90% of genes essential for viability of human cell lines lead to embryonic lethality[6]. Thus, three more genes, *BRF2*, *MRPS30*, and *RPAP2*, could also be considered candidates for causing early pregnancy loss, because, although they have not yet been targeted in mouse knockout projects, they are essential in human cell lines. Prior experimental evidence from animal models can help in elucidating the pathogenic effects of variants with a strong deficit of homozygosity that do not reach significance (Supplementary Data 20).

## Incomplete homozygous deficit

In addition to genes where we observed 10% or less of predicted homozygotes, we also examined variants in genes with a less pronounced deficit (Supplementary Data 4 and 6), with the aim to detect sequence variants in known or novel disease genes that have a pathological impact on individuals primarily after birth.

The F508del-CFTR variant causes autosomal recessive cystic fibrosis, which is the most common lethal monogenic disease in Europeans[9], and is present in ~90% of cystic fibrosis cases[10]. In the combined set, we observe an incomplete homozygous deficit (59%) for the F508del-CFTR variant (expected homozygotes = 211, observed homozygotes = 86) (Supplementary Data 4). In the UK we observe a near complete deficit for F508del-CFTR (expected homozygotes = 117, observed homozygotes = 2, $MAF_{UK}$ = 1.6%). In contrast, in Denmark, the expected numbers of homozygotes did not deviate from HWE ($P$ =0.62, expected homozygotes = 72, observed homozygotes = 70, $MAF_{DNK}$ = 1.3%). Given the pathophysiology of F508del-CFTR and its known impact on life expectancy[11], the vast difference of homozygous deficit of F508del-CFTR between the populations included in the study is likely because of differences in the recruitment strategies used to obtain the population samples, leading to very different age distribution. This possibly also reflects the changing epidemiology of cystic fibrosis which went from being a uniformly fatal disease of infancy to one in which the median age of survival approaches 50 years of age[11].

The stop-gained variant p.Glu70Ter in *IVD*, which is specific to the Icelandic population, is likely an example of incomplete homozygous deficit. Six homozygotes are expected, but we only observe two (Supplementary Data 4). Missense and pLOF variants in *IVD* cause recessive isovaleric acidemia (OMIM:607036). In Iceland, one homozygote of p.Glu70Ter had a diagnostic code of disorders of branched-chain amino acid metabolism (ICD-10: E71.1) indicative of isovaleric acidemia. Out of a total of 20 offspring of five *IVD* carrier couples of the stop gained variant p.Glu70Ter, four non-genotyped offspring are recorded to have died early in life, two in the first year of life, one at seven, and one at 26 years of age. This indicates that homozygosity of p.Glu70Ter in IVD has variable expressivity which might explain the incomplete homozygous deficit.

# Homozygous deficit variants in known Mendelian genes

## *DHCR7*

Consistent with our previous finding[1], we observe the most prominent homozygous deficit for the splice acceptor variant c.946-1G>C in the *DHCR7* gene (expected homozygotes = 93, observed homozygotes = 0) (Table 1). This variant is a loss-of-function mutation causing Smith-Lemli-Opitz syndrome (SLOS), a severe recessive congenital syndrome caused by variants in *DHCR7* (OMIM:270400). For c.946-1G>C in *DHCR7* which has the most prominent homozygous deficit in the current study, in a few reported cases, homozygosity leads to either early miscarriage and intrauterine fetal demise or severe Smith-Lemli-Optiz syndrome and death before three months of age[12,13]. The c.946-1G>C variant is the most frequently identified pathogenic *DHCR7* allele, and it is reported in at least one-third of all SLOS cases, but it is almost exclusively found in compound heterozygotes[12,14] (OMIM:602858.0001, ClinVar Variation ID:6776). It has also been speculated that Smith-Lemli-Optiz syndrome and specifically the c.946-1G>C splice variant may be responsible for a high number of miscarriages, and it has been estimated that 43–88% of homozygotes are miscarried[12,15]. In our data, the splice acceptor variant c.946-1G>C is a top-eQTL in adipose tissue, and associates with strongly reduced *DHCR7* mRNA levels ($P$ =5.2×10$^{-7}$, Effect = -1.07 SD, or -19.1% $CI_{95\%}$[-25.1-12.7%]). The variant results in the usage of an alternative acceptor splice-site downstream of the variant. This causes a 134 base pair intron retention, resulting in a frameshift and nonsense-mediated decay of the mutated transcript (Supplementary Figure 5).

We observe an excess of miscarriage in 75 pregnancies among 21 carrier couples of the c.946-1G>C variant (n miscarriages/n pregnancies = 24/75) ($P$ =8.5×10$^{-6}$) (Supplementary Data 8). Furthermore, in the case of carrier couples, we also observe that a significantly higher proportion has been affected by miscarriage (couples affected /couples total = 14/21) compared non-carrier control couples (OR = 5.34 [95%CI: 2.02-15.7], $P$ =1.9×10$^{-4}$; Fisher-test) (Supplementary Data 19). However, we do not observe an effect for couples where one partner is a carrier when comparing ever versus never miscarriage status in Icelandic among women who answered a routine pregnancy history questionnaire (OR = 1.04 [95%CI: 0.94-1.15], $P$ = 0.45) (Supplementary Data 19).

In Iceland, among all genotyped individuals (n = 154,085), we observe 27 heterozygous couples with 60 offspring. As we have previously reported, two children of heterozygous couples were recorded to die in their first year[1]. As expected based on population-level data, among the 26 genotyped offspring of carrier couples for c.946-1G>C in *DHCR7*, no homozygous carriers are observed (13 are heterozygotes, 13 homozygotes for the reference allele[G]).

Additionally, we observe a genome-wide significant association of c.946-1G>C in *DHCR7* (only carried by heterozygotes) with a large increasing effect on Vitamin D levels in a meta-analysis including 445K individuals from Iceland and the UK (Effect = 0.18 SD, $P$ = $1.1\times10^{-60}$). Multiple variants have been reported at the *DHCR7* and the neighboring *NADSYN1* loci[16]. The c.946-1G>C splice acceptor variant in *DHCR7* is independent of the reported lead variants for vitamin D at the locus[16]. To our knowledge, no association of the *DHCR7* splice variant with vitamin D has been reported on a population scale in the literature (EBI GWAS Catalog).

*DHCR7* is an enzyme that catalyzes the last step in the biosynthesis of cholesterol and thus controls the availability of 7-dehydrocholesterol for conversion to vitamin D[17]. Reduced DHCR7 enzyme function leads to higher vitamin D levels and is thought to have facilitated early human migration to northern latitudes[18]. The splice acceptor c.946-1G>C is the most common variant causing SLOS in Western Europe and, by extension, in North America (frequency ~ 1.1%)[12]. Haplotype analysis indicates that the c.946-1G>C splice variant arose about 3,000 years ago in North-Western Europe[19], and it is thought to be a founder mutation in the British Isles[20]. Also, a geographic West to East gradient in allele frequency of the *DHCR7* splice variant is observed in Europe, on a common haplotype background[18,20]. In accordance with this, we observe an allele frequency gradient of the variant from West to East in our set of North-Western European populations, Iceland (1.4%) > UK (1.1%) > Denmark (0.53%) > Norway (0.41%) > Sweden (0.28%) > Finland (0.19%).

Taken together, our observations based on large-scale population data support previous knowledge that homozygosity for c.964-1G>C in *DHCR7* is incompatible with life. Additionally, we observe an excess of miscarriages among heterozygous carrier couples. Importantly, we do not observe an association of carrier couples where one partner is a carrier, with an increased risk of miscarriage, which indicates that the effect on miscarriage is recessive. Also, we observe a

heterozygous effect of c.964-1G>C on increased vitamin D levels in the general population, which may signify a heterozygous advantage conferred by this variant.

## *MVD*

In our combined population set, the splice region variant c.70+5G>A in *MVD* shows a significant homozygous deficit (expected homozygotes = 10.9, observed homozygotes = 0) (Table 1). This variant has a similar allele frequency in the five North-Western European populations studied (0.16% – 0.41%), and the highest observed frequency is in the UK Biobank (Supplementary Data 9, and Supplementary Figure 3). No homozygotes are observed in public databases. The highest population frequency of the variant in GnomAD is in non-Finnish Europeans (AF = 0.34%), and among 177 thousand Finns, the allele frequency is lower than in other Europeans (AF = 0.09%). The *MVD*:c.70+5G>A splice variant is a top cis-pQTL, and it associates with strongly reduced levels of MVD in a GWAS of plasma protein levels in 35,559 Icelanders ($\text{Effect}_{adj}$ = -0.77 SD, $P_{adj}$ =$5.0\times10^{-22}$). *MVD* encodes the enzyme mevalonate diphosphate decarboxylase, which catalyzes the final step in the mevalonate pathway, which is essential for the synthesis of isoprenoids and cholesterol[21]. Knocking out the ortholog of the *MVD* gene in mice causes embryonic lethality, and the gene is essential for cell proliferation in human cell lines. Thus, MVD can be designated as a strong candidate developmental lethal gene[6]. MVD has not been linked to recessive Mendelian disease, but two missense variants in *MVD* are reported to cause an autosomal dominant form of porokeratosis (OMIM:614714) in the OMIM database (OMIM:603236.0001, OMIM:603236.0002). The *MVD*:c.70+5G>A is not correlated to the two missense variants and has not been linked to porokeratosis. In our data, the variant associates with an increased risk of congenital malformations of the skin (ICD10-Q82) (OR = 3.7, $P$ =$3.4\times10^{-24}$).

Taken together, we observe a homozygous deficit for the splice region variant c.70+5G>A. This variant is associated with strongly reduced MVD protein levels, indicating that the splice region variant is a loss-of-function allele. Also, heterozygotes are at increased risk of congenital skin disorders. Thus, our results suggest that MVD enzyme activity is required for embryonic development and postnatal viability and that reduced levels lead to a pathological state.

## *PMM2*

We observe the most significant homozygous deficit among missense variants for PMM2:p.Arg141His (expected homozygotes = 54, observed homozygotes = 4). The gene *PMM2* encodes an enzyme that is necessary for the synthesis of GDP-mannose. Deficiency of PMM2 causes the most frequently diagnosed type of disorder of glycosylation (CDG), biallelic CDG type 1a, under a recessive mode of inheritance[22] (OMIM:212065). The PMM2:p.Arg141His variant is the most frequently reported pathogenic variant and is present in most reported cases of CDG-1a[23–25](OMIM:601785.0001, ClinVar Variation ID:7706). In our data, the PMM2:p.Arg141His variant is a top cis-pQTL and associates with strongly reduced plasma protein levels of PMM2 in heterozygotes ($\text{Effect}_{adj}$ = -1.0 SD, $P_{adj}$ =$1.0\times10^{-75}$). Consistently, *in vitro* studies have demonstrated that this variant completely abolishes PMM2 enzymatic acitvity[26]. The PMM2:p.Arg141His variant is almost exclusively found in compound heterozygotes that carry one allele with residual enzymatic activity, and homozygosity is absent from patient populations[27]. In our data, the allele frequency of the variant is similar in the five North-Western Europen populations under study, ranging from 0.53% to 0.69% (Supplementary Figure 3). In the GnomAD database, the highest population frequency is observed in Finnish Europeans (AF = 0.84%). Consistently, in our data, a homozygous deficit is also observed among 177 thousand Finns ( expected homozygotes = 11, observed homozygotes = 0)[25,28], where the allele frequency is slightly higher (AF = 0.69%) than in other Europeans.

Notably, we observe four individuals with a homozygous genotype, two in Norway and two in the UK, and these four individuals do not have diagnostic codes or quantitative measures indicative of CDG. The complete loss of PMM2 activity is probably not compatible with life[24] and only a few cases of (nearly) normal PMM2:p.Arg141His homozygous carriers are reported in the literature[29,30]. We observe no evidence supporting an excess of miscarriage in ten pregnancies among four carrier couples of the PMM2:p.Arg141His variant.

## *PNKP*

In our combined population set, gene burden of loss-of-function variants in *PNKP* show a homozygous deficit (expected homozygotes = 8.2, observed homozygotes = 0) (Table 1). The most common loss-of-function allele in *PNKP* is the splice donor variant c.1029+2T>C

(expected homozygotes = 5.4, observed homozygotes = 0) (Supplementary Data 8). The variant's allele frequency ranges from 0.14% in Sweden to 0.30% in Iceland in the five North-Western European populations studied. The variant is absent among 177 thousand Finns, and in GnomAD the highest population frequency is observed in non-Finnish Europeans where no homozygotes are observed (AF = 0.2%). *PNKP* encodes polynucleotide kinase 3'-phosphatase, which is an important DNA repair enzyme[31], and biallelic mutations in the gene cause early infantile epileptic encephalopathy (OMIM:613402), ataxia-oculomotor apraxia type 4 (OMIM:616267) and a type of Charcot–Marie–Tooth disease (OMIM:605589). The *PNKP*:c.1029+2T>C variant has been reported multiple times in the ClinVar database as pathogenic or likely pathogenic in cases with biallelic ataxia-oculomotor apraxia or early infantile epileptic encephalopathy (Variation ID: 206401). Interestingly, the variant is exclusively found in the compound heterozygous state, which suggests a gene dose effect on the pathogenicity of PNKP variants as has been described for many enzymes[32,33].

In our data, the *PNKP:*c.1029+2T>C splice donor variant is a top cis-pQTL and associates with strongly reduced plasma protein levels of PNKP in heterozygotes ($Effect_{adj}$ = -0.93 SD, $P_{adj}$ =$3.3\times10^{-44}$). These results indicate that the *PNKP* splice donor variant is a loss-of-function allele that is likely to abolish PNKP enzymatic activity in the homozygous state. Thus, based on our results, some level of normal PNKP function seems to be required for embryonic development and postnatal viability in humans. Consistent with this, experimental evidence indicates that *PNKP* is likely a developmental lethal gene as it has been demonstrated that it is essential for cell proliferation in human cell lines and homozygous inactivation of *PNKP* causes embryonic lethality in mice[6]. Furthermore, it has been shown in several cancer types that variable level of PNKP inactivation in PTEN depleted tumors leads to synthetic lethality which has led to the development of PNKP inhibitors as potential cancer drugs. Interestingly, a missense variant in *PNKP* with a carrier frequency of 4.7% has been demonstrated to show a complete homozygous deficit in purebred pig populations[34] (Supplementary Data 20).

## Homozygous deficit variants in genes not linked to Mendelian disease

### *ATP5PB*

The stop gained variant p.Arg185Ter in *ATP5PB* has an allele frequency of 1.0% in Iceland, but absent from the other five populations, and exhibits a complete homozygous deficit (expected homozygotes = 15.8, observed homozygotes = 0) (Table 1). Knocking out the *ATP5PB* gene causes embryonic lethality in mice, is essential for proliferation in human cell lines, and thus, is a strong candidate developmental lethal gene[6]. Furthermore, inactivation of *ATP5PB* orthologs causes embryonic lethality across animal model systems[35,36,37–39](Supplementary Data 20). The *ATP5PB* gene encodes ATP synthase peripheral stalk-membrane subunit-Beta, which is a component of complex V($F_1F_0$-ATPase), which is the terminal enzyme in oxidative phosphorylation[40]. *ATP5PB* is not linked to a Mendelian disease.

In Iceland, we observe 27 couples that are heterozygous for p.Arg185Ter, with 59 offspring. We observe no signs of severe congenital conditions or early death among the offspring. In total 28 offspring are genotyped, and none are homozygous (18 are heterozygotes, 10 homozygotes for the reference allele[T]).

Taken together, we observe a pronounced homozygous deficit of p.Arg185Ter in ATP5PB in Iceland, but we do not observe evidence for an excess of reported miscarriage among carrier couples despite substantial power to detect a difference. This suggests that homozygosity of ATP5PB:p.Arg185Ter leads to early embryonic lethality.

### *CCDC59*

In our combined population set, a loss-of-function variant c.561_564+4del in *CCDC59* has a homozygous deficit of 19 (expected homozygotes = 20.7, observed homozygotes = 0) (Table 1). The *CCDC59*:c.561_564+4del variant is an eight-base pair deletion that is predicted to disrupt the splice donor site at the end of exon 3 of the *CCDC59* gene sequence. The frequency of this variant is similar in all of the six populations (0.23% to 0.48%). Homozygous *CCDC59* knockout mice have been reported to show early embryonic lethality before oranogenesis[7] and, *CCDC59* is essential for proliferation in human cell lines. Consequently, we hypothesize that *CCDC59* is a developmental lethal gene[6]. However, the *CCDC59* gene has not been reported to

cause disease in humans[41]. The *CCDC59* gene encodes TAP26, which is a transcription factor that plays a role in surfactant metabolism[42].

In Iceland, we identified seven heterozygous carrier couples of the *CCDC59* splice donor variant. We do not observe a history of early lethality or indication of severe congenital conditions among the 16 offspring. Furthermore, we neither observe an excess of reported miscarriage in the set of 22 pregnancies among the seven carrier couples (n miscarriages /n pregnancies = 5/22) (Supplementary Data 19). Among the nine genotyped offspring of carrier couples, (five are heterozygotes, and four are homozygous for the reference allele).

Taken together, the data are consistent with the hypothesis that, as in mice, homozygosity for *CCDC59* loss of function causes embryonic lethality in humans.

## *MRPS30*

The missense variant p.Ile233Arg in MRPS30 shows a significant homozygous deficit (expected homozygotes = 48.2, observed homozygotes = 1) (Table 1). This variant has a similar allele frequency in the six populations studied (0.34 – 1.05%). Notably, we observe a single homozygote that was detected in Denmark. The *MRPS30* gene has not been knocked out in mice, but it is essential for proliferation in human cell lines[6]. Since cell essential genes are effectively a subset of mouse-lethal genes, we designate MRPS30 as a plausible candidate developmental lethal gene[6] (Table 1). MRPS30, also known as programmed cell death 9 (PDCD9), is a nuclear-encoded mitochondrial ribosomal protein S30. Little is known about the role of nuclear-encoded mitochondrial ribosomal proteins during mammalian embryogenesis, but results from mouse knockout studies indicate a role in the initiation of gastrulation[43]. The fruit-fly ortholog is lethal when inactivated and might correspond to a classic ‘Minute’ locus which typically harbors genes encoding ribosomal proteins[44] (Supplementary Data 20). The missense variant Ile233Arg is located at a conserved site in the MRPS30 protein sequence (Supplementary Figure 12).

In the Icelandic population, we observe four couples that are heterozygous for the p.Ile233Arg variant in *MRPS30*. We do not observe a history of early lethality or information

indicative of severe congenital conditions among seven offspring. Furthermore, we do not observe a significantly higher incidence of miscarriage in carrier couples.

## *BRF2*

We detect a significant deficit of homozygous genotypes in Iceland for the splice donor variant c.214+1G>A at the end of exon 2 of *BRF2* (expected homozygotes = 10.5, observed homozygotes = 0). The *BRF2*:c.214+1G>A variant is not observed outside of Iceland and is essentially absent in public databases. Only three alleles are observed among 125 thousand whole-exome sequenced individuals in the GnomAD database.

*BRF2* (also known as *TFIIIB50*) encodes a subunit of the BRF2–TFIIIB complex, which is involved in the recruitment of RNA polymerase III to genes with type 3 promoters that transcribe short, abundant nonprotein-coding RNA transcripts that have key functional roles, particularly in the protein synthesis apparatus[45].

*BRF2* is an oncogene, and increased gene expression levels have been detected in several types of cancers, including melanoma, gastric, kidney, esophageal, and lung cancers[46]. Furthermore, gene inactivation experiments in human cell lines have shown that *BRF2* is essential for cell proliferation[6]. However, the effect of *BRF2* loss-of-function sequence variation in living organisms is unknown. Such mutations have not been linked to severe clinical phenotypes in humans and are yet to be introduced in mice models.

In our data, we do not observe a significant excess of reported miscarriage in 58 pregnancies among the 19 carrier couples compared to non-carrier couples matched on year of birth and number of pregnancies ($P$ = 0.19) (Table 3). Also, we do not observe an excess in the proportion of couples affected by miscarriage (OR = 1.23 [95%CI: 0.53, 2.55], $P$ = 0.56; Fisher-test) (Supplementary Data 19). One of the 49 offspring of heterozygous couples was recorded to die in the first year of life. However, we do not have a record of diagnostic codes or quantitative measures indicative of severe congenital disease among these offspring.

## *GTF2H3*

In our combined population set, a start-lost variant in *GTF2H3* shows a complete homozygous deficit (expected homozygotes = 9.16, observed homozygotes = 0). The frequency of this variant ranges from 0.13 in Finland to 0.51% in the UK. The highest observed frequency

in the GnomAD database is 0.27% in non-Finnish Europeans (observed homozygotes = 0), and the variant is very rare or absent in non-European populations.

*GTF2H3* encodes a core subunit of the TFIIH basal transcription factor that regulates RNA polymerase II transcription and is involved in nucleotide excision repair[47]. The severe autosomal-recessive disorders Xeroderma Pigmentosum (OMIM:278730), Cockayne Syndrome (OMIM:610651), and Trichothio-dystrophy (OMIM:616390, OMIM:601675, OMIM:616395) are caused by mutations in genes encoding other TFIIH core subunits (*ERCC3*, *ERCC2*, and *GTF2H5*)[47,48].

The *GTF2H3* gene is predominantly expressed in the inner cell mass of mouse blastocysts[49]. Knockout of *Gtf2h3* in mice leads to embryonic lethality prior to the tooth bud stage. Also, *GTF2H3* has been shown to be essential for cell proliferation in human cell lines[6] (Supplementary Data 20). Consequently, GTF2H3 is a promising candidate developmental lethal gene[6].

## *ELOF1*

We detect a frame-shift variant in *ELOF1* showing complete homozygous deficit in the Icelandic set (expected homozygotes = 8.8, observed homozygotes = 0). *ELOF1* encodes the evolutionary conserved zinc-finger protein called Elongation Factor 1 Homolog[50], which has recently been shown to function as a transcription-elongation and DNA repair factor[51,52]. During transcription of an undamaged strand, *ELOF1* works as an elongation factor. However, when coming across DNA lesions caused by UV light or a bulky DNA lesion, *ELOF1* takes part in a DNA repair mechanism called transcription-coupled nucleotide excision repair (TC-NER)[51,52]. *ELOF1* regulates the ubiquitylation of the main RNA Pol II subunit for the recruitment of TFIIH, required for the excision repair. Studies also suggest *ELOF1* takes part in DNA replication[51,52]. Knockout of *Elof1* in mice shows that the gene is essential for viability during mammal gastrulation (Supplementary Data 20), and so far, mutations in *ELOF1* have not been observed in any TC-NER-related syndromes[53,54].

## *RPAP2*

We detect a frame-shift variant in *RPAP2* showing a complete homozygous deficit in Iceland (expected homozygotes = 6.4, observed homozygotes = 0). Frameshift variant

chr1:92333464 (Hg38) is located at exon 9 out of 13. Only eight alleles are observed among 125 thousand whole-exome sequenced individuals in the GnomAD database, of which all but one are in non-Finnish Europeans and no homozygous carrier. *RPAP2* encodes the RNA Pol II-associated protein 2, which is required to shuttle RNA Pol II to the nucleus from the cytoplasm[55,56] and also functions as a phosphatase of Ser5 of the RNA Pol II Carboxy-Terminal Domain (CTD). The CTD consists of heptapeptide repeats of consensus Tyr1-Ser2-Pro3-Thr4-Ser5-Pro6-Ser7, which is highly conserved from bacteria to mammals, where the number of repeats differs[57]. The phosphorylation state of Ser2, Ser5, and Ser7 changes throughout the transcription process performed by the RNA Pol II and replacements of any of the serines is incompatible with life[58]. For transcription of snRNA, Ser7 phosphorylation recruits RPAP2, which dephosphorylates Ser5, which gives access to factors required for snRNA transcription[55]. The *RPAP2* gene has not been targeted in mice but has been shown to be essential for cell proliferation in human cell lines (Table 1).

Interestingly, a splice variant in *RPAP2* with a carrier frequency of 21% in a purebred cattle population shows a complete homozygous deficit due to early embryonic lethality. This variant is also associated with a strong negative effect on reproduction in the same population[59] (Supplementary Data 20).

## *CASP9*

We observe a significant deficit of individuals homozygous for the missense variant c.His237Pro in *CASP9,* which encodes the cysteine-aspartic protease caspase 9. In the combined dataset, 11.9 c.His237Pro homozygotes are expected, and one is observed.

Casp9 is a pro-apoptotic protein that acts as a regulator of physiological cell death and degeneration of pathological tissues. More specifically, caspase 9 is an initiator caspase that can activate downstream effector caspases and trigger a signaling cascade that induces apoptotic cell death[60]. Experiments have shown that *CASP9* knockout mice have an enlarged and malformed cerebrum as a result of reduced apoptosis during development, and a majority of them die perinatally. Furthermore, in vivo, deletion of *CASP9* has been shown to inhibit activation of the downstream effector caspase, Casp3, and *CASP*9 knockout thymocytes show resistance to a number of apoptotic stimuli[61].

The missense variant p.His237Pro disrupts the caspase active site that is highly conserved between the twelve caspase genes in the human genome[62] (Supplementary Figure 13). A prior report has described a family with recurrent, folate-resistant, neural tube defects where two affected fetuses were heterozygous for both c.His237Pro and c.924dupT, a frameshift variant in CASP9[63]. In vitro experiments of cells transfected with the missense variant have shown that the mutation impairs the cellular response to apoptosis and that mutant Casp9 protein has a dominant-negative effect on wild-type Casp9[64].

## MTG2

In our combined population set of Iceland, the UK, and Finland, a frameshift variant in *MTG2* shows a complete homozygous deficit (expected homozygotes = 12, observed homozygotes = 0). The variant is vanishingly rare in Iceland and the UK, whereas it is present at a MAF of 1,1% in Finland. In the Gnomad database, the frameshift variant p.Gly191AlafsTer14 in *MTG2* is observed in 417 mostly European (both Finnish and non-Finnish) individuals, out of 150 thousand whole-exome sequenced individuals. No case of homozygous carriers was observed. The frameshift is caused by a deletion of a single nucleotide in exon 5 out of 7 exons. The *MTG2* gene encodes the mitochondrial ribosome-associated GTPase 2, also known as GTP-binding protein 5 (GTPBP5). GTPBP5 is involved in the assembly of the mitochondrial ribosome 55S by facilitating modification of the 16S mt-rRNA[65,66], which is part of the 39S large subunit. *MTG2* knockouts using gene editing in HEK293 cell lines have been shown to lead to severely affected oxidative phosphorylation in mitochondria, reduction in the synthesis rate of mtDNA-encoded proteins, and decreased 55S monosome formation[65]. *Mtg2* mouse knockouts show embryonic lethality in early gestation (E9.5)[7].

# Supplementary Note 1

## Members of the DBDS Genomic Consortium

Steffen Andersen[1], Karina Banasik[2], Søren Brunak[2], Kristoffer Burgdorf[3], Christian Erikstrup[4], Thomas Folkmann Hansen[5] , Henrik Hjalgrim[6], Gregor Jemec[7], Poul Jennum[8], Pär Ingemar Johansson[3], Kasper Rene Nielsen[9], Mette Nyegaard[10], Mie Topholm Brun[11], Ole Birger Pedersen[12], Susan Mikkelsen[13], Khoa Manh Dinh[13], Erik Sørensen[3], Henrik Ullum[3], Sisse Rye Ostrowski[3], Thomas Werge[14], Daniel Gudbjartsson[15], Kari Stefansson[15], Hreinn Stefánsson[15], Unnur Þorsteinsdóttir[15], Margit Anita Hørup Larsen[3], Maria Didriksen[3], Susanne Sækmose[16]

1. Department of Finance, Copenhagen Business School, Copenhagen, Denmark
2. Novo Nordisk Foundation, Center for Protein Research, Faculty of Health and Medical Sciences, University of Copenhagen, Copenhagen, Denmark
3. Department of Clinical Immunology, Copenhagen University Hospital, Copenhagen, Denmark
4. Department of Clinical Immunology, Aarhus University Hospital, Aarhus, Denmark
5. Danish Headache Center, Department of Neurology, Rigshospitalet, Glostrup, Denmark
6. Department of Epidemiology Research, Statens Serum Institut, Copenhagen, Denmark
7. Department of Clinical Medicine, Zealand University Hospital, Roskilde, Denmark
8. Department of Clinical Neurophysiology at University of Copenhagen, Copenhagen, Denmark
9. Department of Clinical Immunology, Aalborg University Hospital, Aalborg, Denmark
10. Department of Biomedicine, Aarhus University, Denmark
11. Department of Clinical Immunology, Odense University Hospital, Odense, Denmark
12. Department of Clinical Immunology, Zealand University Hospital, Køge, Denmark
13. Department of Clinical Immunology, Aarhus University Hospital, Aarhus, Denmark
14. Institute of Biological Psychiatry, Mental Health Centre Sct. Hans, Copenhagen University Hospital, Roskilde, Denmark
15. deCODE genetics, Reykjavik, Iceland
16. Department of Clinical Immunology, Zealand University Hospital, Køge, Denmark

# Supplementary References


1. Sulem, P. *et al.* Identification of a large set of rare complete human knockouts. *Nat. Genet.* **47**, 448–452 (2015).
2. Gudbjartsson, D. F. *et al.* Large-scale whole-genome sequencing of the Icelandic population. *Nat. Genet.* **47**, 435–444 (2015).
3. Lim, E. T. *et al.* Distribution and medical impact of loss-of-function variants in the Finnish founder population. *PLoS Genet.* **10**, e1004494 (2014).
4. Amorim, C. E. G. *et al.* The population genetics of human disease: The case of recessive, lethal mutations. *PLoS Genet.* **13**, 1–23 (2017).
5. Landrum, M. J. *et al.* ClinVar: public archive of interpretations of clinically relevant variants. *Nucleic Acids Res.* **44**, D862–8 (2016).
6. Dawes, R., Lek, M. & Cooper, S. T. Gene discovery informatics toolkit defines candidate genes for unexplained infertility and prenatal or infantile mortality. *NPJ Genom Med* **4**, 8 (2019).
7. Dickinson, M. E. *et al.* High-throughput discovery of novel developmental phenotypes. *Nature* **537**, 508–514 (2016).
8. Xue, L. *et al.* Global expression profiling reveals genetic programs underlying the developmental divergence between mouse and human embryogenesis. *BMC Genomics* **14**, 568 (2013).
9. Ratjen, F. & Döring, G. Cystic fibrosis. *Lancet* **361**, 681–689 (2003).
10. Riordan, J. R. CFTR function and prospects for therapy. *Annu. Rev. Biochem.* **77**, 701–726

(2008).

11. Bell, S. C. *et al.* The future of cystic fibrosis care: a global perspective. *Lancet Respir Med* **8**, 65–124 (2020).

12. Nowaczyk, M. J. M., Waye, J. S. & Douketis, J. D. DHCR7 mutation carrier rates and prevalence of the RSH/Smith-Lemli-Opitz syndrome: where are the patients? *Am. J. Med. Genet. A* **140**, 2057–2062 (2006).

13. Daum, H. *et al.* Smith-Lemli-Opitz syndrome: what is the actual risk for couples carriers of the DHCR7:c.964-1G>C variant? *Eur. J. Hum. Genet.* **28**, 938–942 (2020).

14. Lazarin, G. A., Haque, I. S., Evans, E. A. & Goldberg, J. D. Smith-Lemli-Opitz syndrome carrier frequency and estimates of in utero mortality rates. *Prenat. Diagn.* **37**, 350–355 (2017).

15. Lazarin, G. A. *et al.* Systematic Classification of Disease Severity for Evaluation of Expanded Carrier Screening Panels. *PLoS One* **9**, e114391 (2014).

16. Manousaki, D. *et al.* Genome-wide Association Study for Vitamin D Levels Reveals 69 Independent Loci. *Am. J. Hum. Genet.* **106**, 327–337 (2020).

17. Prabhu, A. V., Luu, W., Sharpe, L. J. & Brown, A. J. Cholesterol-mediated Degradation of 7-Dehydrocholesterol Reductase Switches the Balance from Cholesterol to Vitamin D Synthesis. *J. Biol. Chem.* **291**, (2016).

18. Kuan, V., Martineau, A. R., Griffiths, C. J., Hyppönen, E. & Walton, R. DHCR7 mutations linked to higher vitamin D status allowed early human migration to northern latitudes. *BMC Evol. Biol.* **13**, 144 (2013).

19. Witsch-Baumgartner, M. *et al.* Age and origin of major Smith-Lemli-Opitz syndrome (SLOS) mutations in European populations. *J. Med. Genet.* **45**, 200–209 (2008).

20. Witsch-Baumgartner, M. *et al.* Frequency gradients of DHCR7 mutations in patients with Smith-Lemli-Opitz syndrome in Europe: evidence for different origins of common mutations. *Eur. J. Hum. Genet.* **9**, 45–50 (2001).

21. Goldstein, J. L. & Brown, M. S. Regulation of the mevalonate pathway. *Nature* **343**, 425–430 (1990).

22. Van Schaftingen, E. & Jaeken, J. Phosphomannomutase deficiency is a cause of carbohydrate-deficient glycoprotein syndrome type I. *FEBS Lett.* **377**, 318–320 (1995).

23. Kjaergaard, S., Skovby, F. & Schwartz, M. Absence of homozygosity for predominant mutations in PMM2 in Danish patients with carbohydrate-deficient glycoprotein syndrome type 1. *Eur. J. Hum. Genet.* **6**, 331–336 (1998).

24. Jaeken, J., Lefeber, D. & Matthijs, G. Clinical utility gene card for: Phosphomannomutase 2 deficiency. *Eur. J. Hum. Genet.* **22**, (2014).

25. Erlandson, A. *et al.* Scandinavian CDG-Ia patients: genotype/phenotype correlation and geographic origin of founder mutations. *Hum. Genet.* **108**, 359–367 (2001).

26. Matthijs, G., Schollen, E., Heykants, L. & Grünewald, S. Phosphomannomutase deficiency: the molecular basis of the classical Jaeken syndrome (CDGS type Ia). *Mol. Genet. Metab.* **68**, 220–226 (1999).

27. Andreotti, G., Pedone, E., Giordano, A. & Cubellis, M. V. Biochemical phenotype of a common disease-causing mutation and a possible therapeutic approach for the phosphomannomutase 2-associated disorder of glycosylation. *Mol Genet Genomic Med* **1**, 32–44 (2013).

28. Schollen, E., Kjaergaard, S., Legius, E., Schwartz, M. & Matthijs, G. Lack of Hardy-Weinberg equilibrium for the most prevalent PMM2 mutation in CDG-Ia (congenital

disorders of glycosylation type Ia). *Eur. J. Hum. Genet.* **8**, 367–371 (2000).

29. Vuillaumier-Barrot, S. *et al.* Expanding the Spectrum of PMM2-CDG Phenotype. *JIMD Rep.* **5**, 123–125 (2012).

30. Grünewald, S. The clinical spectrum of phosphomannomutase 2 deficiency (CDG-Ia). *Biochim. Biophys. Acta* **1792**, 827–834 (2009).

31. Gatti, M. *et al.* From congenital microcephaly to adult onset cerebellar ataxia: Distinct and overlapping phenotypes in patients with PNKP gene mutations. *Am. J. Med. Genet. A* **179**, 2277–2283 (2019).

32. Veitia, R. A. & Birchler, J. A. Dominance and gene dosage balance in health and disease: why levels matter! *J. Pathol.* **220**, (2009).

33. Veitia, R. A., Caburet, S. & Birchler, J. A. Mechanisms of Mendelian dominance. *Clin. Genet.* **93**, 419–428 (2018).

34. Derks, M. F. L. *et al.* Loss of function mutations in essential genes cause embryonic lethality in pigs. *PLoS Genet.* **15**, e1008055 (2019).

35. Clark, K. J. *et al.* In vivo protein trapping produces a functional expression codex of the vertebrate proteome. *Nat. Methods* **8**, 506–515 (2011).

36. Mummery-Widmer, J. L. *et al.* Genome-wide analysis of Notch signalling in Drosophila by transgenic RNAi. *Nature* **458**, 987–992 (2009).

37. Gönczy, P. *et al.* Functional genomic analysis of cell division in C. elegans using RNAi of genes on chromosome III. *Nature* **408**, 331–336 (2000).

38. Colaiácovo, M. P. *et al.* A targeted RNAi screen for genes involved in chromosome morphogenesis and nuclear organization in the Caenorhabditis elegans germline. *Genetics* **162**, 113–128 (2002).

39. Simmer, F. *et al.* Genome-wide RNAi of C. elegans using the hypersensitive rrf-3 strain reveals novel gene functions. *PLoS Biol.* **1**, E12 (2003).

40. Hock, D. H., Robinson, D. R. L. & Stroud, D. A. Blackout in the powerhouse: clinical phenotypes associated with defects in the assembly of OXPHOS complexes and the mitoribosome. *Biochem. J* **477**, 4085–4132 (2020).

41. OMIM. *Online Mendelian Inheritance in Man, OMIM®. McKusick-Nathans Institute of Genetic Medicine, Johns Hopkins University (Baltimore, MD), November 11, 2018. World Wide Web URL: https://omim.org/* https://omim.org/.

42. Yang, M. C. W., Wang, B., Weissler, J. C., Margraf, L. R. & Yang, Y. S. BR22, a 26 kDa thyroid transcription factor-1 associated protein (TAP26), is expressed in human lung cells. *Eur. Respir. J.* **22**, 28–34 (2003).

43. Cheong, A. *et al.* Nuclear-encoded mitochondrial ribosomal proteins are required to initiate gastrulation. *Development* **147**, (2020).

44. Marygold, S. J. *et al.* The ribosomal protein genes and Minute loci of Drosophila melanogaster. *Genome Biol.* **8**, R216 (2007).

45. Turowski, T. W. & Tollervey, D. Transcription by RNA polymerase III: insights into mechanism and regulation. *Biochem. Soc. Trans.* **44**, 1367–1375 (2016).

46. Yeganeh, M. & Hernandez, N. RNA polymerase III transcription as a disease factor. *Genes Dev.* **34**, 865–882 (2020).

47. Rimel, J. K. & Taatjes, D. J. The essential and multifunctional TFIIH complex. *Protein Sci.* **27**, 1018–1037 (2018).

48. Singh, A., Compe, E., Le May, N. & Egly, J.-M. TFIIH subunit alterations causing xeroderma pigmentosum and trichothiodystrophy specifically disturb several steps during

transcription. *Am. J. Hum. Genet.* **96**, 194–207 (2015).

49. Yoshikawa, T. *et al.* High-throughput screen for genes predominantly expressed in the ICM of mouse blastocysts by whole mount in situ hybridization. *Gene Expr. Patterns* **6**, 213–224 (2006).
50. Daniels, J.-P., Kelly, S., Wickstead, B. & Gull, K. Identification of a crenarchaeal orthologue of Elf1: implications for chromatin and transcription in Archaea. *Biol. Direct* **4**, 24 (2009).
51. van der Weegen, Y. *et al.* ELOF1 is a transcription-coupled DNA repair factor that directs RNA polymerase II ubiquitylation. *Nat. Cell Biol.* **23**, 595–607 (2021).
52. Geijer, M. E. *et al.* Elongation factor ELOF1 drives transcription-coupled repair and prevents genome instability. *Nat. Cell Biol.* **23**, 608–619 (2021).
53. Laugel, V. Cockayne syndrome: the expanding clinical and mutational spectrum. *Mech. Ageing Dev.* **134**, 161–170 (2013).
54. Tellier, A. P., Archambault, D., Tremblay, K. D. & Mager, J. The elongation factor Elof1 is required for mammalian gastrulation. *PLoS One* **14**, e0219410 (2019).
55. Egloff, S., Zaborowska, J., Laitem, C., Kiss, T. & Murphy, S. Ser7 phosphorylation of the CTD recruits the RPAP2 Ser5 phosphatase to snRNA genes. *Mol. Cell* **45**, 111–122 (2012).
56. Forget, D. *et al.* Nuclear import of RNA polymerase II is coupled with nucleocytoplasmic shuttling of the RNA polymerase II-associated protein 2. *Nucleic Acids Res.* **41**, 6881–6891 (2013).
57. Eick, D. & Geyer, M. The RNA polymerase II carboxy-terminal domain (CTD) code. *Chem. Rev.* **113**, 8456–8490 (2013).
58. Zhang, D. W. *et al.* Ssu72 Phosphatase-dependent Erasure of Phospho-Ser7 Marks on the

RNA Polymerase II C-terminal Domain Is Essential for Viability and Transcription Termination*. *J. Biol. Chem.* **287**, 8541–8551 (2012).

59. Guarini, A. R. *et al.* Estimating the effect of the deleterious recessive haplotypes AH1 and AH2 on reproduction performance of Ayrshire cattle. *J. Dairy Sci.* **102**, 5315–5322 (2019).

60. Avrutsky, M. I. & Troy, C. M. Caspase-9: A Multimodal Therapeutic Target With Diverse Cellular Expression in Human Disease. *Front. Pharmacol.* **12**, 701301 (2021).

61. Kuida, K. *et al.* Reduced apoptosis and cytochrome c-mediated caspase activation in mice lacking caspase 9. *Cell* **94**, 325–337 (1998).

62. Denault, J.-B. & Salvesen, G. S. Caspases: keys in the ignition of cell death. *Chem. Rev.* **102**, 4489–4500 (2002).

63. Spellicy, C. J. *et al.* Key apoptotic genes APAF1 and CASP9 implicated in recurrent folate-resistant neural tube defects. *Eur. J. Hum. Genet.* **26**, 420–427 (2018).

64. Cardone, M. H. *et al.* Regulation of cell death protease caspase-9 by phosphorylation. *Science* **282**, 1318–1321 (1998).

65. Cipullo, M., Gesé, G. V., Khawaja, A., Hällberg, B. M. & Rorbach, J. Structural basis for late maturation steps of the human mitoribosomal large subunit. *Nat. Commun.* **12**, 3673 (2021).

66. Cipullo, M. *et al.* Human GTPBP5 is involved in the late stage of mitoribosome large subunit assembly. *Nucleic Acids Res.* **49**, 354–370 (2021).
