# Supplementary material for: Deficit of homozygosity among 1.52 million individuals and genetic causes of recessive lethality
Source: Nat Commun. 2023 Jun 10;14:3453. doi: 10.1038/s41467-023-38951-2 (PMC10257723; doi:10.1038/s41467-023-38951-2)
Supplement: Supplementary file 3 — Description of Additional Supplementary Files [file 41467_2023_38951_MOESM3_ESM.pdf]

File name: Supplementary Data 1-23

Description: Supplementary data as a separate excel sheets (SupData 1-23).

Supplementary Data 1

Descriptive statistic for the datasets included in the current study.

Supplementary Data 2

Single variants belonging to pLOF, moderate impact, low Impact, and intergenic variant classes. Variants count by expected homozygous count bins in the combined set of 1.52 million individuals. After binning variants based on the expected number of homozygotes and functional impact, the fraction of protein-altering variants (pav) with a strong deficit of homozygosity (f pav) in each bin was compared to that of intergenic variants (f intergenic) to estimate an FDR (FDR = f intergenic/f pav).

Supplementary Data 3

Single variants by dataset belonging to pLOF, moderate impact, low Impact, and intergenic variant classes. Variants count by expected homozygous count bins in the combined set of 1.52 million individuals, true positive rate are relative to intergenic variants.

Supplementary Data 4

Gentoype counts, single variants (pLOF and Moderate impact)

Column name	Description	Source
Variant	Variant genomic position (Hg38) alternative and reference allele	
Gene Symbol	Gene symbol	HGNC Database, www.genenames.org.
Max Consequence	Max functional consequence assigned to variants by the Variant Effect predictor (VEP)	
Dataset	Population datasets: D = Denmark, I = Iceland, U = UK, N = Norway, S = Sweden	
obs HOM comb	Combined number of homozygotes observed	
obs HOM	Number of homozygotes observed within each population	
exp HOM comb	Combined number of homozygotes expected	

Column name	Description	Source
exp HOM	Number of homozygotes observed within each population	
obs exp ratio	Ratio of the observed and expected homozygout count	
MAF pct	Allele frequency within each population (%)	
comb P HW	The probability of observing "q" homozygotes with an expectation "lambda"• estimated using the Poisson distribution	
HGVSp	Protein altering variant description according to Human Genome Variation Society standards(HGVS)	
Max Impact	Max functional impact class assigned to variant by the Variant Effect predictor (VEP)	
OMIM ID	Mendelian disease linked to gene	Online Mendelian Inheritance in Man, www.omim.org
OMIM inheritance	Mode of inheritance of the linked Mendelian disease	Online Mendelian Inheritance in Man, www.omim.org
Stillbirth mortality info	Evidence of stillbirth or early mortality in humans	
cell essentiality	Essentiality status for cell growth in human cell-lines	DepMap
mouse mortality	Information of viability status from heterozygous crosses in mice	Mouse Genome Informatics and International mouse phenotyping consortium: Impc.org, informatics.jax.org
life stage	Developmental stage of embryonic lethality	International mouse phenotyping consortium: Impc.org
CLINVAR CLINSIG	ClinVar clinical significance and the number of submissions	www.ncbi.nlm.nih.gov/clinvar/
clinvar ALT	Alternative allele of variant submitted to ClinVar (join is by genomic position)	www.ncbi.nlm.nih.gov/clinvar/
low confidence lof	flagged as low-confidence pLOF by the LoFTee algorithm	https://github.com/konradjk/loftee

Supplementary Data 5

geneLOF count by expected homozygous count bins in the combined set of 1.52 million individuals. After binning variants based on the expected number of homozygotes and functional impact, the fraction of geneLOFs with a strong deficit of homozygosity (f pav) in each bin was compared to that of intergenic variants (f intergenic) to estimate an FDR (FDR = f intergenic/f pav).

Supplementary Data 6

Gentoype counts, geneLOFs

Column name	Description	Source
Chromosome	chromosomal location	HGNC Database, www.genenames.org.
Gene Symbol	Gene symbol	HGNC Database, www.genenames.org.
NCBI Gene ID	Stable gene ID	HGNC Database, www.genenames.org.
dataset	population datasets: D = Denmark, I = Iceland, U = UK, N = Norway, S = Sweden	
comb P pois	The probability of observing "q" homozygotes with an expectation "lambda"• estimated using the Poisson distribution	
obs HOM comb	Combined number of homozygotes observed	
obs HOM	Number of homozygotes observed within each population	
exp HOM comb	Combined number of homozygotes expected	
exp HOM	Number of homozygotes observed within each population	
obs exp ratio	Ratio of the observed and expected homozygout count	
MAF pct	Allele frequency within each population (%)	
OMIM ID	Mendelian disease linked to gene	Online Mendelian Inheritance in Man, www.omim.org
OMIM inheritance	Mode of inheritance of the linked Mendelian disease	Online Mendelian Inheritance in Man, www.omim.org

Column name	Description	Source
stillbirth mortality info	Evidence of stillbirth or early mortality in humans	
Panel	Fetal anomalies gene panel	Genomics England PanelApp
Panel info	gene panel review status	Genomics England PanelApp
cell	Essentiality status for cell growth in human cell-lines	https://depmap.org/portal/download
mouse mortality	Information of viability status from heterozygous crosses in mice	Mouse Genome Informatics and International mouse phenotyping consortium: Impc.org, informatics.jax.org
life stage	Developmental stage of embryonic lethality	International mouse phenotyping consortium: Impc.org
ortholog relationship hs to mm	Ortholog relationship of gene between human and mouse	Mouse Genome Informatics : informatics.jax.org
Gene group name	Name given to a gene group the gene has been assigned to	HGNC Database, www.genenames.org.
Locus type	Specifies the type of locus described by the given entry	HGNC Database, www.genenames.org.
Locus group	Groups locus types together into related sets	HGNC Database, www.genenames.org.

Supplementary Data 7

human KO genes

Column name	Description	Source
gene	gene symbol	
Sulem	gene with rare biallelic predicted loss-of-function (pLOF): Yes = 1, No = 0	Sulem, P. et al. Identification of a large set of rare complete human knockouts. Nat. Genet. 47, 448–452 (2015)
Narasimhan	gene with rare biallelic predicted loss-of-function (pLOF): Yes = 1, No = 0	Narasimhan, V. M. et al. Health and population effects of rare gene knockouts in adult humans with related parents. Science 352, 474–477 (2016)
Saleheen	gene with rare biallelic predicted loss-of-function (pLOF): Yes = 1, No = 0	Saleheen, D. et al. Human knockouts and phenotypic analysis in a cohort with a high rate of consanguinity. Nature Publishing Group 544, 235–239 (2017)
gnomAD	gene with rare biallelic predicted loss-of-function (pLOF): Yes = 1, No = 0	Karczewski, K. J. et al. The mutational constraint spectrum quantified from variation in 141,456 humans. bioRxiv 531210 (2020) https://doi.org/10.1101/531210
Oddsson	gene with rare biallelic predicted loss-of-function (pLOF): Yes = 1, No = 0	Current study: Supplementary Data S1
row sum	"row sum of gene KO status (Yes = 1) from Sulem,Narasimhan,Saleheen,gnomAD, and Oddsson	"

Supplementary Data 8

Predicted loss-of-function (pLOF) and moderate impact sequence variants with strong deficit of homozygosity.

Supplementary Data 9

Allele frequency of homozygous deficit variants in the GnomAD (gnomAD exomes r2.1.1, n = 125,748 individuals; gnomAD genomes v3.1.1, n = 15,708 individuals) and TOPMed databases (TOPMed Freeze 8 genomes, n = 132,345 individuals).

Supplementary Data 10

pLOFs included in gene test (geneLOFs)

Column name	Description
Chrom	Chromosome location
Pos	genomic position (Hg38)
REF	Reference allele
ALT	Alternative allele
MAF	Imputed minor allele frequency
ImpInfo	Imputation info
Gene Symbol	Gene symbol
Max Consequence	Max functional consequence assigned to variants by the Variant Effect predictor (VEP)
strata	Population dataset: D = Denmark, I = Iceland, U = UK, N = Norway, S = Sweden
ID	ClinVar ID
Stars	ClinVar star rating
Guidelines	ACMG clinical practive guidelines
clinvar ALT	Alternative allele of variant submitted to ClinVar (join is by genomic position)
CLINVAR CLINSIG	ClinVar clinical significance and the number of submissions

Supplementary Data 11

Top cis-eQTls among homozgous deficit variant. RNA sequencing was performed on whole blood from Icelanders (n = 13,175).

Supplementary Data 12

Top splice-QTls among homozgous deficit variant. RNA sequencing was performed on whole blood from Icelanders (n = 13,175).

Supplementary Data 13

Top cis-pQTls among homozgous deficit variant from a GWASs of plasma protein levels of 4,907 aptamers that measure 4,719 proteins in 35,559 Icelanders (Ferkingstad et al., PMID: 34857953).

Supplementary Data 14

OMIM inheritance

Column name	Description	Source
Gene Symbol	Gene symbol	HGNC Database, www.genenames.org.
omim id	OMIM disease ID	Online Mendelian Inheritance in Man, www.omim.org
inheritance	Mode of inheritance: A = autosomal, X = X-linked, D = dominant, R = recessive	Online Mendelian Inheritance in Man, www.omim.org
disease name	Disease name	Online Mendelian Inheritance in Man, www.omim.org

Supplementary Data 15

cell essentiality

Column name	Description	Source
Gene Symbol	Gene symbol	HGNC Database, www.genenames.org.
NCBI Gene ID	Stable gene ID	HGNC Database, www.genenames.org.
Achilles common essential	List of genes identified as pan-essentials using Chronos	https://depmap.org/portal/download
CRISPR common essential	List of genes identified as dependencies	https://depmap.org/portal/download
common essential	Intersection of Biomen (2014) and Hart (2015) essentials	https://depmap.org/portal/download
non essential	List og genes tested but not deemed as essential for cell growth	https://depmap.org/portal/download
Chromosome	Chromosome location	HGNC Database, www.genenames.org.
Locus group	Groups locus types together into related sets.	HGNC Database, www.genenames.org.
Gene group name	Groups genes together into related functional sets.	HGNC Database, www.genenames.org.

Supplementary Data 16

Mouse viability

Column name	Description	Source

Column name	Description	Source
Gene Symbol	Gene symbol of orthologous human gene	HGNC Database, www.genenames.org.
phenotype	Viability related phenotype in mouse: viable, sub-viable, lethal	Mouse Genome Informatics and International mouse phenotyping consortium: Impc.org, informatics.jax.org

Supplementary Data 17

geneLOFs, excluding low-confidence LoF (LoFtee) with deficit of homozygotes and an homozygout count between 2 and 5.

Supplementary Data 18

Sequence variants with deficit of homozygosity and an expected homozygote count between one and five.

Supplementary Data 19

Excess miscarriage in Icelandic couples that are carriers of homozygous deficit pLOF variants among 61,848 genotyped couples from Iceland were the female partner answered a routine pregnancy history questionnaire in a healthcare setting between 1964 and 1994.

Supplementary Data 20

Evidence of lethality in various animal models for orthologs of genes carrying variants deficit of homozygosity.

Supplementary Data 21

Early acting recessive lethal candidate genes based on being essential for growth of human cell lines. Sequence variants with a homozygous deficit with expected homozygous count over 2 for pLOF, and 2.5 for moderate impact.

Supplementary Data 22

Sets of intergenic variants located 5 kb, 50 kb, 100 kb, 250 kb, and 500 kb outside of annotated genic regions

Supplementary Data 23

Cancer rate among 87,078 Icelandic women who answered a routine pregnancy history questionnaire in a healthcare setting between 1964 and 1994.
