# Supplementary material for: Deficit of homozygosity among 1.52 million individuals and genetic causes of recessive lethality
Source: Nat Commun. 2023 Jun 10;14:3453. doi: 10.1038/s41467-023-38951-2 (PMC10257723; doi:10.1038/s41467-023-38951-2)
Supplement: Supplementary file 5 — Reporting Summary [file 41467_2023_38951_MOESM5_ESM.pdf]

nature portfolio

Corresponding author(s): Patrick Sulem, Kari Stefansson

Last updated by author(s): Mar 13, 2023

Reporting Summary

Nature Portfolio wishes to improve the reproducibility of the work that we publish. This form provides structure for consistency and transparency in reporting. For further information on Nature Portfolio policies, see our Editorial Policies and the Editorial Policy Checklist.

Statistics

For all statistical analyses, confirm that the following items are present in the figure legend, table legend, main text, or Methods section.

n/a	Confirmed	
☐	☒	The exact sample size (*n*) for each experimental group/condition, given as a discrete number and unit of measurement
☐	☒	A statement on whether measurements were taken from distinct samples or whether the same sample was measured repeatedly
☐	☒	The statistical test(s) used AND whether they are one- or two-sided<br>*Only common tests should be described solely by name; describe more complex techniques in the Methods section.*
☐	☒	A description of all covariates tested
☐	☒	A description of any assumptions or corrections, such as tests of normality and adjustment for multiple comparisons
☐	☒	A full description of the statistical parameters including central tendency (e.g. means) or other basic estimates (e.g. regression coefficient) AND variation (e.g. standard deviation) or associated estimates of uncertainty (e.g. confidence intervals)
☐	☒	For null hypothesis testing, the test statistic (e.g. *F*, *t*, *r*) with confidence intervals, effect sizes, degrees of freedom and *P* value noted<br>*Give P values as exact values whenever suitable.*
☒	☐	For Bayesian analysis, information on the choice of priors and Markov chain Monte Carlo settings
☒	☐	For hierarchical and complex designs, identification of the appropriate level for tests and full reporting of outcomes
☒	☐	Estimates of effect sizes (e.g. Cohen's *d*, Pearson's *r*), indicating how they were calculated

Our web collection on statistics for biologists contains articles on many of the points above.

Software and code

Policy information about availability of computer code

Data collection	inio software was used for data collection.
Data analysis	We used publicly available software in conjunction with methods developed at deCODE Genetics as described in the methods section.<br>Graphtyper version 2, https://github.com/DecodeGenetics/graphtyper;<br>We used R, version 4.1.0 and Julia, version 1.8 extensively to analyze data and create plots;<br>Multi-trait analysis of genome-wide association summary statistics version 1, https://github.com/JonJala/mtag;<br>PANTHER v.16.0, http://www.pantherdb.org/tools/;<br>Variant Effect Predictor (release 100), https://github.com/Ensembl/ensembl-vep;<br>BOLT-LMM version 2.1, https://data.broadinstitute.org/alkesgroup/BOLT-LMM/downloads/;<br>IMPUTE2 version 2.3.1, https://mathgen.stats.ox.ac.uk/impute/impute_v2.html;<br>dbSNP version 140, http://www.ncbi.nlm.nih.gov/SNP/;<br>STAR software package, version 2.7.10, https://github.com/alexdobin/STAR;<br>Ensembl version 87, https://www.ensembl.org/index.html;<br>LeafCutter version 1, https://github.com/davidaknowles/leafcutter;<br>kallisto version 0.46, https://github.com/pachterlab/kallisto;<br>SAMtools, version 1.3, http://samtools.github.io/;<br>BWA-MEM version 0.7.10, https://github.com/lh3/bwa,<br>GenomeAnalysisTKLite version 2.3.9, https://github.com/broadgsa/gatk/,<br>Picard tools version 1.117, https://broadinstitute.github.io/picard/,

For manuscripts utilizing custom algorithms or software that are central to the research but not yet described in published literature, software must be made available to editors and reviewers. We strongly encourage code deposition in a community repository (e.g. GitHub). See the Nature Portfolio guidelines for submitting code & software for further information.

Data

Policy information about availability of data

All manuscripts must include a data availability statement. This statement should provide the following information, where applicable:

- Accession codes, unique identifiers, or web links for publicly available datasets
- A description of any restrictions on data availability
- For clinical datasets or third party data, please ensure that the statement adheres to our policy

Genotype counts for protein-altering-variants presented in this study are included in this publication and its Supplementary information and Supplementary Data.
Sequence variants tested have been deposited in the European Variation Archive under accession number PRJEB15197 (https://www.ebi.ac.uk/ena/browser/view/PRJEB15197).
FinnGen data are publicly available and were downloaded from https://finngen.fi/.
The UKB data was downloaded under application number 56270.
Proteomics data and protein mapping to UniProt identifiers and gene names were provided by SomaLogic and Olink.
Information on the mode of inheritance of Mendelian disease and linked genes was extracted from the Inheritance subontology of The Human Phenotype Ontology (HPO)40 (http://purl.obolibrary.org/obo/hp/hpoa/phenotype.hpoa)
Data on genes essential for the growth of human cell lines were derived from genome-wide screens were downloaded from Project Achilles41,42 website (https://depmap.org/portal/download)
A unified list of of common essential genes from three gene sets was used (Achilles_common_essentials.csv, CRISPR_common_essentials.csv, and Common_essentials.csv)
Data on mouse lethal phenotypes was retrieved from the Mouse Genome Informatics (MGI) database (http://www.informatics.jax.org/downloads/reports/MGI_GenePheno.rpt) and the International Mouse Phenotyping Consortium (IMPC). The 15th release of IMPC mouse phenotype data was downloaded from the IMPC ftp site (http://ftp.ebi.ac.uk/pub/databases/impc/all-data-releases/release-15.1/results/viability.csv.gz).
To estimate the age of selected variants, human genome dating database was used (https://human.genome.dating)
Other data presented in this study are included in this publication and its Supplementary information.

Human research participants

Policy information about studies involving human research participants and Sex and Gender in Research.

Reporting on sex and gender	Regardless of sex or gender, the study's results are applicable to anyone. Sex of the participants were taken into account in the analysis when matching carrier couples by genotype to assess miscarriage risk. A more detailed can be found in the methods section.
Population characteristics	A detailed description of population characteristics can be found in the methods section.<br>The UK Biobank project is a large prospective cohort study of "500,000 individuals from across the United Kingdom, aged between 40-69 years at recruitment. In the UK Biobank 46% recruited were male, 54% female. 57% aged 40-59 years; 43% aged 60-69 years.<br>The Icelandic deCODE genetics study is based on whole-genome sequence data from 49,708 Icelanders participating in various research projects at deCODE genetics. Variants identified through whole-genome sequencing were imputed into 155,250 chip-genotyped Icelanders as well as their untyped close relatives based on genealogy.<br>Finngen summary statistics, including fatty liver disease and cirrhosis, were imported on December 2020 from a source available to researchers (version 4; https://www.finngen.fi/en/access_results) and methods have been documented (https://finngen.gitbook.io/documentation/).<br>The Copenhagen Hospital Biobank Cardiovascular Study (CHB- CVDC) was used to acquire secondary cardiovascular phenotypes. CHB-CVDC involves a targeted selection of patients with cardiovascular disease from the CHB, a biobank based on patient blood samples drawn in Danish hospitals. For binary phenotypes, the control group included blood donors from The Danish Blood Donor Study (DBDS).<br>Norwegian genotype data were obtained from both hospital and population-based samples. Clinical samples included data from the DemGene and TOP studies which consist of case control samples of neuropsychiatric disorders.<br>Genotypic data from Sweden was primarily retrieved from disease-specific population-based case-control studies on chronic inflammatory diseases, including studies on multiple sclerosis (EIMS).
Recruitment	For the deCODE Genetics study individuals were recruited through various research projects at deCODE genetics. The participants are a large fraction of the adult Icelandic population.<br>UK Biobank holds data on half a million participants throughout the UK. All participants in UK Biobank were recruited through assessment centres, designed specifically for this purpose.<br>The US data are individuals recruited at the Intermountain healthcare.<br>The FinnGen database consists of samples collected from Finnish biobanks.<br>CHB-CVDC involves a targeted selection of patients with cardiovascular disease from the CHB, a biobank based on patient blood samples drawn in Danish hospitals.<br>Norwegian genotype data were obtained from both hospital and population-based samples. Clinical samples included data from the DemGene and TOP studies which consist of case control samples of neuropsychiatric disorders.<br>Genotypic data from Sweden was primarily retrieved from disease-specific population-based case-control studies on chronic inflammatory diseases, including studies on multiple sclerosis (EIMS).
Ethics oversight	All participating subjects in the deCODE genetics study who donated blood signed informed consent. The personal identities

Ethics oversight

of the participants and biological samples were encrypted by a third-party system. The study was approved by the Icelandic Data Protection Authority and the National Bioethics Committee of Iceland (no VSN-20-182).
The CHB-CVDC has been approved by The National Committee on Health Research Ethics (1708829) and the Danish Data Protection Agency (P-2019-93). The Danish Blood Donor Study (DBDS), approved by the Danish Data Protection Agency (P-2019-99) and the Scientific Ethical Committee system (NVC 1700407).
The FinnGen database consists of samples collected from the Finnish biobanks and and phenotype data collected at the national health registers. The Coordinating Ethics Committee of the Helsinki and Uusimaa Hospital District evaluated and approved the FinnGen research project. The project complies with existing legislation (in particular the Biobank Law and the Personal Data Act). The official data controller of the study is University of Helsinki.
The UK Biobank Resource was used under application number 56270. All phenotype and genotype data were collected following an informed consent obtained from all participants. The North West Research Ethics Committee reviewed and approved UK Biobank's scientific protocol and operational procedures (REC Reference Number: 06/MRE08/65).
For the Intermountain dataset, the Intermountain Healthcare Institutional Review Board approved the study, and all participants provided written informed consent prior to enrollment.
Norwegian genotype data were obtained from both hospital and population-based samples. Clinical samples included data from the DemGene and TOP studies which consist of case control samples of neuropsychiatric disorders. Written informed consent was obtained, and the Regional Committee for Medical and Health Research Ethics (REC) South East (#2009/2485) and Mid Norway (#2014/631) approved the studies. Population-based samples included data from the Norwegian Mother, Father and Child cohort study (Mor og Barn; MoBa) and the Hordaland Health Study (HUSK). MoBa is a population-based pregnancy cohort study conducted by the Norwegian Institute of Public Health. Participants were recruited from all over Norway from 1999-2008. The women provided consent to participation in 41% of the pregnancies. The cohort includes approximately 114,500 children, 95,200 mothers and 75,200 fathers. Blood samples were obtained from both parents during pregnancy and from mothers and children (umbilical cord) at birth. For a more detailed description of the MoBa sample see Magnus et a15,6. The current study included genotype data from 168,000 mothers, fathers and offspring . The establishment of MoBa and initial data collection was based on a license from the Norwegian Data Protection Agency and approval from the REC. The MoBa cohort is currently regulated by the Norwegian Health Registry Act. Written informed consent was obtained from all mothers and fathers participating in MoBa. The current study was approved by REC South East (#2016/1226). MoBa is supported by the Norwegian Ministry of Health and Care Services and the Ministry of Education and Research. We are grateful to all the participating families in Norway who take part in this on-going cohort study. The HUSK Study is a community-based prospective study conducted in Hordaland County in Norway (http://husk.b.uib.no). The project was approved by REC (Western Norway 2018/915), and written informed consent was obtained from all participants. Genotypic data was provided by the HARVEST collaboration (supported by the Research Council of Norway (RCN) (#229624), the NORMENT Centre (RCN #223273) South East Norway Health Authorities and Stiftelsen Kristian Gerhard Jebsen; in collaboration with deCODE Genetics, and the Center for Diabetes Research at the University of Bergen (funded by the ERC AdG project SELECTionPREDISPOSED, Stiftelsen Kristian Gerhard Jebsen, Trond Mohn Foundation, the RCN , the Novo Nordisk Foundation, the University of Bergen, and the Western Norway Health Authorities).
Genotypic data from Sweden was primarily retrieved from disease-specific population-based case-control studies on chronic inflammatory diseases, including studies on multiple sclerosis (EIMS)7,8 (04/252 1-4 & 2019-00639) and STOPMS2 (2009/2107-31/2 & 2020-0712), approved by National Ethical review board, GEMS9, IMSE10, and IMSE2 (2011/641-31/4), STOPMS11 (02-548), and COMBATMS12 (2017/32-31/4) approved by The Stockholm Regional Ethical Review Board, and rheumatoid arthritis (EIRA, Umea)13,14. The original rheumatoid arthritis studies were approved by the Swedish Ethical Review Authority and all data have been de-identified prior to analyses. Furthermore, genotypic data from the Swedish National Myeloma Biobank15,16 (Swedish Ethical Review Authority; Dnr 2019-06386), Skane University Hospital, Lund, and from Swedish blood donors and primary care patients aged 18 to 71 years from Skane countyl7 (Lund University Ethics Review Board; Dnr 2018/2) were also included. The original studies were approved by the Lund University Ethical

Note that full information on the approval of the study protocol must also be provided in the manuscript.

Field-specific reporting

Please select the one below that is the best fit for your research. If you are not sure, read the appropriate sections before making your selection.

☒ Life sciences ☐ Behavioural & social sciences ☐ Ecological, evolutionary & environmental sciences

For a reference copy of the document with all sections, see nature.com/documents/nr-reporting-summary-flat.pdf

Life sciences study design

All studies must disclose on these points even when the disclosure is negative.

Sample size

Sample sizes are reported in the article and correspond to all available data.
We performed a power analysis to estimated the sample size required to detect a strong deficit of homozygosity with 80% power, as well as the power to detect the effect of a strong deficit of homozygosity on minor allele frequencies between 0 and 1.6% (see Supplementary information for details).

Data exclusions

No available data was excluded from the study

Replication

The homozygous deficit analysis was performed using data from 6 populations (Iceland, UK, Denmark, Norway, Sweden and Finland) and results across populations were compared. The supplementary data contain results on all genotypes tested, which enable the comparison of replications between populations.

Randomization	Not applicatble (not a randomized trial). As the sample size of the current study is large, the potential for bias is reduced and randomization is not necessary.
Blinding	Not applicable (not a randomized trial, so no blinding is required). Since the current study does not require researcher-based judgments, blinding is not necessary

Reporting for specific materials, systems and methods

We require information from authors about some types of materials, experimental systems and methods used in many studies. Here, indicate whether each material, system or method listed is relevant to your study. If you are not sure if a list item applies to your research, read the appropriate section before selecting a response.

Materials & experimental systems

n/a	Involved in the study
☒	☐ Antibodies
☒	☐ Eukaryotic cell lines
☒	☐ Palaeontology and archaeology
☒	☐ Animals and other organisms
☒	☐ Clinical data
☒	☐ Dual use research of concern

Methods

n/a	Involved in the study
☒	☐ ChIP-seq
☒	☐ Flow cytometry
☒	☐ MRI-based neuroimaging
