# Supplementary material for: Deficit of homozygosity among 1.52 million individuals and genetic causes of recessive lethality
Source: Nat Commun. 2023 Jun 10;14:3453. doi: 10.1038/s41467-023-38951-2 (PMC10257723; doi:10.1038/s41467-023-38951-2)
Supplement: Supplementary file 2 — Peer Review File [file 41467_2023_38951_MOESM2_ESM.pdf]

nature portfolio

Peer Review File

Deficit of homozygosity among 1.52 million individuals and genetic causes of recessive lethality

Open Access This file is licensed under a Creative Commons Attribution 4.0 International License, which permits use, sharing, adaptation, distribution and reproduction in any medium or format, as long as you give appropriate credit to the original author(s) and the source, provide a link to the Creative Commons license, and indicate if changes were made. In the cases where the authors are anonymous, such as is the case for the reports of anonymous peer reviewers, author attribution should be to 'Anonymous Referee' followed by a clear attribution to the source work. The images or other third party material in this file are included in the article's Creative Commons license, unless indicated otherwise in a credit line to the material. If material is not included in the article's Creative Commons license and your intended use is not permitted by statutory regulation or exceeds the permitted use, you will need to obtain permission directly from the copyright holder. To view a copy of this license, visit http://creativecommons.org/licenses/by/4.0/.

REVIEWER COMMENTS</B>

Reviewer #1 (Remarks to the Author):

In this manuscript, Asmundur Oddsson and colleagues have cast light on the genetic bases of recessive lethality by scrutinizing the genomes of 1.52 million individuals from six North-Western European populations inhabiting Denmark, Finland, Iceland, Norway, Sweden, and the UK. By conducting rigorous analyses, the authors have reported a set of 25 genes carrying homozygous deficit protein-altering variants, of which 11 genes are not known to cause any Mendelian disease previously. Through gene set over-representation analysis and gene expression profiling, they characterized more than thousand genes with predicted LOFs and confirmed their important role in cell growth. They also presented a catalogue of homozygous deficit variants in genes linked and unlinked to Mendelian disease for further clinical evaluations.

Overall, this study is comprehensive and the results are well presented, although some of the aspects could be clear by following the supplementary data in great detail. In line with the authors' previous Icelandic population-based explorations, this study is interesting with important findings and significant clinical implications with regard to recessive lethality.

I have the following minor comments that should be addressed in a revision:

1) In the results section, the authors mention "selection" and "negative selection" of homozygous variants. But there is no information on the type of selection test(s) performed in support of their statements.

2) In the discussion section, page 13 paragraph 2, the following statement, "This variant is present in all of the European populations considered with an allelic frequency ranging from 0.3% to 1%, indicating that it is ancient in origin", needs supporting reference or suitable age estimates, may be from the Atlas of Variant Age (https://human.genome.dating/).

3) In the discussion section, page 13, in the following sentence, "Further studies are required to understand the biological impact of p.Ile233Arg in MRPS30 (Supplementary Fig. 4)." the referenced supplementary fig. 4 is not relevant.

4) In the supplementary fig.1 legend, the following line is incomplete, "The blue line indicates true positive rate cutoff of…"

5) In the supplementary information page 28, correct the typo "homozygout"

6) In the supplementary information page 35, under the sub-title, Incomplete homozygous deficit, rephrase the following sentences:

“In addition to genes where we observed 10% or less of predicted homozygotes, we also examinedscrutinize variants in genes with a less pronouncedprofound deficit (Supplementary Data S1 and S2), with the aim of. This approach can detecting sequence variants in known or novel disease genes that have a pathological impact on individuals primarily after birththe effect of genotypes, in known or novel disease genes, occurring after birth.”

7) In the supplementary information page 36 paragraph 2, correct the typos and rephrase the following sentence, “….due to the differentces in the recruitment strategies used to obtain the population samples, leading to very different age distributionage structures and recruitment strategies between the populations.”

8) In the supplementary information page 36 paragraph 3, correct the typo, “heterozygoutes” and present the missing information: ? = 0.18 SD, P = 1.1×10-60.

9) In the supplementary information page 39, present the missing information: ?adj = -0.77 SD, Padj =5.0×10-22.

10) I noticed that the special character “β” is missing throughout the supplementary information text, most likely lost during file format conversion. As suggested above in 7 & 8 please fix this issue.

11) In general, some of the supplementary figures are not in order of appearance in the text and makes it difficult to follow.

12) As the supplementary information is crucial for this manuscript, the authors should correct the typos, and double-check the titles, legends and order of the figures and tables in alignment with the text.

Reviewer #2 (Remarks to the Author):

By scanning the genotype data of over 1.5 million individuals of North European descent, this study identified 25 genes with rare genetic variants that show significant deficit of homozygosity. These genes are significantly enriched for genes known to cause recessive Mendelian diseases, as well as essential genes in human cell lines or mice. In addition, the authors found that couples who are both heterozygous for the identified variants with deficit of homozygosity are more likely to experience miscarriages, for the top gene DHCR7 individually or for the collection of 25 genes in aggregate. Lastly, the authors confirmed that many of the identified variants are indeed associated with reduced gene expression (in terms of RNA or protein). These observations were taken together as evidence suggesting that the homozygotes of the identified variants are under strong negative selection, as complete loss of the corresponding genes may cause pregnancy loss or severe Mendelian disease.

Conceptually, the paper is very similar to previous studies looking for naturally existing gene knockouts in human populations, but in outbred populations and with a greater sample size. I am largely convinced that homozygosity of the variants they identified may cause recessive lethality with high penetrance, and some of these genes may be underlying pregnancy loss. However, the evidence presented does not

show that the variants described are complete loss-of-function or fully recessive. In addition, the statistical analysis performed is not sufficiently or clearly described in some places. Below are my specific concerns and suggestions.

(1) The fact that some of the identified variants are in high linkage disequilibrium with eQTLs or pQTLs with negative effect sizes does not necessarily suggest that they are complete loss-of-function variants, as they can also be hypomorphic mutations. On the other hand, the observation that carrier mothers, when coupled with non-carrier fathers, are not more likely to experience miscarriages does not support full recessiveness, because the effects in carrier mothers may be too small to detect given current sample size, or that the carriers have slightly lower fitness compared to non-carriers due to non-fertility reasons. The authors should either weaken these claims or present additional evidence (e.g., experimentally demonstrating complete loss-of-function in cell lines using gene editing technique).

(2) The usage of "true positive rate (TPR)" is incorrect, which creates a lot of confusion in paragraphs on page 8. Typically, TPR is also known as sensitivity or recall, which measures the ratio of true positives to all real positives. In this paper, the ratio of (fraction of pLOF or other types of coding variants with deficit of homozygosity) to (fraction of intergenic variants with deficit of homozygosity) is more like positive predictive value (PPV), precision, or (1-false discovery rate), which measures the ratio of true positives to total number of called positives. Please revise in text as well as in legend of Figure 2.

(3) It is unclear how the test for miscarriage rate among carrier couples was done. Was the Fisher's exact test done on the number of couples (experiencing at least one miscarriage vs never) or the number of pregnancy outcomes (miscarriages vs registered births)? If the authors used either, can they also perform test with the other method to see if the result is robust to the choice?

(4) I agree with the authors' assessment that given the current sample size, power is limited. It would improve the paper to perform some power analysis to quantitatively evaluate the power of detecting variants with homozygosity deficit with the current criteria used.

(5) The authors briefly discussed the total number of genes with homozygous knockouts found in human population so far and provided a summary in Supplementary Data S3. These results, while useful, may be mis-interpreted as evidence for being non-pathogenic or compatible with survival, although some of them could still cause severe condition or even lethality due to incomplete penetrance. It would be helpful if the authors can explicitly discuss the utility and limitations of these results in Discussion to guide application in clinical settings.

Minor comments

(1) The current observations can be ordered differently to make the logic connection easier to follow. In my opinion, association of the identified variants to reduced expression can be presented right after identification of the variants with homozygosity deficit, as a sanity check of the predicted impacts. Then the comparison with Mendelian disease genes and essential genes can be presented and lead to the hypothesis that some of these variants may cause pre-natal lethality instead of post-natal disorder, which is further tested by the analysis of miscarriage rate in carrier couples.

(2) In lines 228-229, the method for calculating p-value (i.e., assuming Poisson distribution) should be included in the parentheses in addition to the Bonferroni p-value threshold.

(3) Perhaps I am not following but I find it confusing that the numbers in the text sometimes do not match those in the tables. For example, in lines 245-247, it reads “11 are located in genes that have not been reported to cause a Mendelian condition… The remaining 14 genes are reported to have variants causing a Mendelian condition…” but in Table 3, 10 and 9 genes with expected homozygotes>=5 are in OMIM AR genes versus not, respectively. Later, in line 259-260, it says “Sequence variants in 12 of the 25 genes causes Mendelian disease under a recessive mode…” I’m very confused by the small differences in numbers.

(4) Similarly, in lines 312-314, it says “based on being essential for growth of human cell lines, 13 genes…” but in Table 3, 11 genes with expected homozygotes>=5 are essential in human cell line.

Reviewer #3 (Remarks to the Author):

Here the authors present a unique opportunity to detect rare recessive (and potentially lethal) variation. The authors do this by combining large genotyping and sequencing datasets. They proceed to identify multiple genes that could potentially be linked to embryonic lethality and approximately 1000 genes with previously undetected predicted loss of function variants.

The paper is concise and reads well for the intended audience of the journal. I think with additional interrogation of the statistic and variants it will provide strong evidence for previously unknown lethal variants.

Below I list major comments and a number of minor comments detailed below.

Method comments:

1) Given the amount of data that is being imputed and merged here I am wondering what the final set of variants used for each of the analyses was? It seems like there was a population specific set of variants and maybe a combined set? Additionally, was there any QC done on the final dataset once the whole-genome sequence was imputed into the chip-typed individuals?

2) Have the authors thought about how imputation and sequencing error could be factored into the variant level analyses? I think this is especially important given the very low frequency variants that this work is aimed at identifying.

a) Another thought is what is the mappability like in each of these regions? I don't think that the results here are caused by poor mappability, given the other layers of support, but it would be good to check.

3) How are intergenic regions found? I think this is especially important given that the TPR metric is based on these intergenic regions being as close to neutral as possible.

a) I might suggest authors try making a couple sets of these intergenic variants to account for the effect of linked selection. For example, the putatively neutral sites have different genetic distances to the nearest gene (e.g. 0cM, 0.2cM,0.4cM…1cM) to calibrate the TPR statistic that is being used to identify strong directional selection in the putative lethals.

b) Also, could the authors add a section about the intergenic variation to the methods where it is calibrated based on distance from genes or other highly conserved regions?

4) When thinking about Figure 2, I am wondering if the authors could explore what is going on with the moderate impact variants in the [3,5) and [5,8) bins? There should be a decent number of moderate impact variants (at least more than pLOF and geneLOF), so I am wondering why the TPR is depleted for these two bins then jumps up dramatically for [8,13)?

5) I had a couple questions about the λ statistic for identifying the deficit of homozygotes. I will frame questions specifically in the context of Table 1.

a) My first question is whether the p-value corrected for multiple testing? I don't believe that it is. Can authors comment on this and maybe add a sentence to the caption and/or methods?

b) Second question, is there a correction for structure for pooled sample? One thing that I worry about when combining all this data together, then computing the p-value, is how structure could be affecting the statistic. My interpretation of the methods section led me to think that there is not a correction. We know from previous papers that there is structure within and across European populations. So, perhaps the p-value should be computed within each population if there is no correction? Or maybe the authors could add a frequency-based correction?

6) If we think of a site where I expect 5 homozygotes and observe 0 in a population, it seems like that could happen about 1% of the time (~e-5/0!). I am wondering if the authors could spot check a few of the genes (e.g., CCDC59) by looking at reads from a handful of sequenced individuals in IGV? Do the reads support the site being fixed for the other homozygote or heterozygous?

7) Could the authors elaborate on exactly which individuals are being used for the miscarriage among carrier couples analyses? In the supplementary methods, the authors say that the data comes from Icelandic women who participated in a cohort study for screening breast and cervix cancer at the Icelandic Cancer Center. Did any women used in the analyses have or develop cancer or were the carrier couples only from the control group? My thought would be that one would not be using carrier couples where the mother developed cancer because that could potentially explain the miscarriage rate more so than the carrier status.

8) Lastly, I was thinking about the sequence variants in the final set of 25 genes with the deficit of homozygosity. Are any of those variants at a detectable frequency in other populations from say TOPMED or 1000 Genomes?

Minor Comments:

Main Text:

Abstract: uses pLOF but the term is not defined

Line 204: missing period after publications

Line 236: Tables should be Table

Line 271: The authors say “from most population databases” which databases are being referred to here. Can the authors include citations?

Line 276: extra space before Frameshift

Line 421: extra space before We

Supplement:

General comment for the tables, it is really hard to read the text underneath them, can font size be increased?

For supplementary tables 9-11 can the authors include how p-values are computed and a one sentence declarative for caption?

Supplementary figure 1 has a typo, the last sentence is a fragment and I think is a key assumption for the paper.

Supplementary figure 2, I think observed should be lower case

Page 28 second section: first sentence in first paragraph homozygote is misspelled and first sentence in second paragraph is missing a parenthesis for VEP

Page 29: typo-was less than 10% the expected I think it should be 10% of the

Page 31: extra space before We in first paragraph

Page 32: what does MP abbreviation stand for?

Pages 35-36: The first few sentences of the Incomplete homozygous deficit section have many typos

Page 36: first new paragraph in the second to last sentence, differences is misspelled

Point-by-point answer to reviewers

We thank the three reviewers for their comments. We have made substantial changes to the manuscript according to their comments.

All changes can be reviewed in track changes. Line numbers below refer to edits in the manuscript with track changes.

Reviewer 1 (Remarks to the Author)

In this manuscript, Asmundur Oddsson and colleagues have cast light on the genetic bases of recessive lethality by scrutinizing the genomes of 1.52 million individuals from six North-Western European populations inhabiting Denmark, Finland, Iceland, Norway, Sweden, and the UK. By conducting rigorous analyses, the authors have reported a set of 25 genes carrying homozygous deficit protein-altering variants, of which 11 genes are not known to cause any Mendelian disease previously. Through gene set over-representation analysis and gene expression profiling, they characterized more than thousand genes with predicted LOFs and confirmed their important role in cell growth. They also presented a catalogue of homozygous deficit variants in genes linked and unlinked to Mendelian disease for further clinical evaluations.

Overall, this study is comprehensive and the results are well presented, although some of the aspects could be clear by following the supplementary data in great detail. In line with the authors' previous Icelandic population-based explorations, this study is interesting with important findings and significant clinical implications with regard to recessive lethality.

Reviewer 1: Minor comments

I have the following minor comments that should be addressed in a revision:

Comment #1

In the results section, the authors mention "selection" and "negative selection" of homozygous variants. But there is no information on the type of selection test(s) performed in support of their statements.

Response to comment #1

In the Results section, we now explain in which context we are referring to selection. The sentence at the beginning of paragraph 2 on page 7 now reads, lines 191-199 (changes underlined):

"It is well established that deviations from random mating within a population (such as inbreeding or stratification) tend to increase the number of homozygotes[16]. For sequence variants that increase the risk of deleterious phenotypes among homozygotes, these factors will therefore tend to increase the number of individuals that are exposed to negative selection. In our data, there is an excess of observed homozygotes compared to the expected number of homozygotes under HWE, with rare variants showing the greatest relative excess (Supplementary Fig. 1 and Supplementary Table 2)."

In that chapter, we include a reference to citation 16 (Wright et al., PMID: 17246615) that is consistent with our observation that an increased number of homozygotes are potentially under selection when deviations from random mating occur.

When investigating the deficit of homozygotes and discussing the true positive rate (TPR, which we have now changed to FDR following the suggestion of reviewer #2 in comment #2), we are now specifically claiming that it represents the fraction of variants with a homozygote deficit due to negative selection of a variant among homozygotes.

The sentence at the end of paragraph 2 on page 8, lines 223-226 now reads (changes underlined):

"The FDR estimates the fraction of homozygous deficit variants in each bin due to negative selection rather than chance, under the assumption that homozygosity for intergenic variants is effectively neutral."

Comment #2

In the discussion section, page 13 paragraph 2, the following statement, "This variant is present in all of the European populations considered with an allelic frequency ranging from 0.3% to 1%, indicating that it is ancient in origin", needs supporting reference or suitable age estimates, maybe from the Atlas of Variant Age (https://human.genome.dating/).

Response to comment #2

To provide support for an age estimate, we have added the following sentence to page 14, paragraph 2, lines 406-408 in the revised manuscript (changes underlined):

"This variant is present in all of the European populations considered with an allelic frequency ranging from 0.3% to 1%, indicating that it is ancient in origin. Assuming a generation time of 25 years, the estimated age of the G allele of rs72756207 resulting in the Ile233Arg missense variation of MRPS30 is estimated to be 16,000 years (637 generations) (95% CI: 380-923 generations, 9,500-23,000 years)[39]."

The Supplementary Methods now include a description of the variant age estimation. See subsection "Variant age estimation" on Page 22:

"To estimate the age of selected variants, human genome dating database was used (https://human.genome.dating/snp/rs72756207)[39]. Using the reference allele as the ancestral state, age was estimated for the alternate allele and the generation time was assumed to be 25 years."

Comment #3

In the discussion section, page 13, in the following sentence, "Further studies are required to understand the biological impact of p.Ile233Arg in MRPS30 (Supplementary Fig. 4)." the referenced supplementary fig. 4 is not relevant.

Response to comment #3

The reference to Supplementary Figure 4 has been removed

Comment #4

In the supplementary fig.1 legend, the following line is incomplete, "The blue line indicates true positive rate cutoff of…"

Response to comment #4

The caption of Supplementary Figure 2 in the revised manuscript (previously Supplementary Figure 1) now reads (changes underlined):

"S. Figure 2. Observed/expected homozygote count ratio versus frequency for the combined set of 1.52 million individuals from six countries stratified by impact class. The red line indicates a 90% homozygous deficit. The blue line indicates 5 and 8 expected homozygote count for pLOF and moderate impact variants, respectively, which correspond to a false discovery rate cutoff <10%."

Comment #5

In the supplementary information page 28, correct the typo "homozygout"

Response to comment #5

The typo on page 28 of the supplementary information has been corrected and now reads "homozygote".

Comment #6

In the supplementary information page 35, under the sub-title, Incomplete homozygous deficit, rephrase the following sentences: "In addition to genes where we observed 10% or less of predicted homozygotes, we also examinedscrutinize variants in genes with a less pronouncedprofound deficit (Supplementary Data S1 and S2), with the aim of. This approach can detecting sequence variants in known or novel disease genes that have a pathological impact on individuals primarily after birththe effect of genotypes, in known or novel disease genes, occurring after birth."

Response to comment #6

Under the sub-title, Incomplete homozygous deficit, the sentence has been corrected in the supplementary information on page 26 of the revised manuscript:

"In addition to genes where we observed 10% or less of predicted homozygotes, we also examined variants in genes with a less pronounced deficit (Supplementary Data S1 and S2), with the aim to detect sequence variants in known or novel disease genes that have a pathological impact on individuals primarily after birth."

Comment #7

In the supplementary information page 36 paragraph 2, correct the typos and rephrase the following sentence, "….due to the differentces in the recruitment strategies used to obtain the population samples, leading to very different age distributionage structures and recruitment strategies between the populations."

Response to comment #7

The sentence has been corrected in the supplementary information of the revised manuscript, page 27, paragraph 1 and now reads:

"because of differences in the recruitment strategies used to obtain the population samples, leading to very different age distribution."

Comment #8

In the supplementary information page 36 paragraph 3, correct the typo, "heterozygoutes" and present the missing information: ? = 0.18 SD, P = 1.1×10-60.

Response to comment #8

The typo in the supplementary information on page 28, paragraph 3 in the revised manuscript has been corrected and now reads "heterozygotes".

The missing information has been added and now reads (changes underlined): "Effect = 0.18 SD, P = 1.1×10^{-60}".

Comment #9

In the supplementary information page 39, present the missing information: $?_{adj}$ = -0.77 SD, P_{adj} =5.0×10^{-22}.

Response to comment #9

The missing information in the supplementary information on page 30 of the revised manuscript has been provided and now reads (changes underlined): "Effect_{adj} = -0.77 SD, P_{adj} =5.0×10^{-22} ".

Comment #10

I noticed that the special character "β" is missing throughout the supplementary information text, most likely lost during file format conversion. As suggested above in 7 & 8 please fix this issue.

Response to comment #10

Thanks for noticing this. The special character "β" has been replaced by "Effect" throughout the supplementary information text to avoid errors during file conversion.

Comment #11

In general, some of the supplementary figures are not in order of appearance in the text and makes it difficult to follow.

Response to comment #11

The supplementary figures have now been arranged according to their order in the main text in the revised manuscript.

Comment #12

As the supplementary information is crucial for this manuscript, the authors should correct the typos and double-check the titles, legends, and order of the figures and tables in alignment with the text.

Response to comment #12

Thank you for your attention to this issue. Typos have been corrected, titles and legends have been checked for errors, and the figures and tables have been aligned with the text references in the supplementary information. For the convenience of the reader, the supplement tables have been moved to a separate Excel file.

Reviewer 2 (Remarks to the Author)

By scanning the genotype data of over 1.5 million individuals of North European descent, this study identified 25 genes with rare genetic variants that show significant deficit of homozygosity. These genes are significantly enriched for genes known to cause recessive Mendelian diseases, as well as essential genes in human cell lines or mice. In addition, the authors found that couples who are both heterozygous for the identified variants with deficit of homozygosity are more likely to experience miscarriages, for the top gene DHCR7 individually or for the collection of 25 genes in aggregate. Lastly, the authors confirmed that many of the identified variants are indeed associated with reduced gene expression (in terms of RNA or protein). These observations were taken together as evidence suggesting that the homozygotes of the identified variants are under strong negative selection, as complete loss of the corresponding genes may cause pregnancy loss or severe Mendelian disease.

Conceptually, the paper is very similar to previous studies looking for naturally existing gene knockouts in human populations, but in outbred populations and with a greater sample size. I am largely convinced that homozygosity of the variants they identified may cause recessive lethality with high penetrance, and some of these genes may be underlying pregnancy loss. However, the evidence presented does not show that the variants described are complete loss-of-function or fully recessive. In addition, the statistical analysis performed is not sufficiently or clearly described in some places. Below are my specific concerns and suggestions.

Reviewer 2: Major comments

Comment #1

The fact that some of the identified variants are in high linkage disequilibrium with eQTLs or pQTLs with negative effect sizes does not necessarily suggest that they are complete loss-of-function variants, as they can also be hypomorphic mutations. On the other hand, the observation that carrier mothers, when coupled with non-carrier fathers, are not more likely to experience miscarriages does not support full recessiveness, because the effects in carrier mothers may be too small to detect given current sample size, or that the carriers have slightly lower fitness compared to non-carriers due to non-fertility reasons. The authors should either weaken these claims or present additional evidence (e.g., experimentally demonstrating complete loss-of-function in cell lines using gene editing technique).

Response to comment #1

In response to the reviewer's concerns, we have modified our claims regarding the complete loss-of-function effect of the homozygous deficit variant variants, and the full recessiveness of the effect on excess miscarriage.

The sentence on page 15, line 420, now reads (changes underlined):

“This suggests that the variants that we describe are <u>at least partial</u> loss-of-function variants and that some minimum level of activity is required for successful embryonic development.”

Taking into account the reviewer's concerns, we have revised our calculations and are now comparing the effects of couples in which both partners are carriers versus non-carrier couples, and couples in which one partner is a carrier versus non-carrier couples.

At the very least, the data are consistent with the recessive model. The mode of inheritance does not appear to be additive as the effect in carrier couples where one partner is a carrier is much smaller than predicted under the additive model. Overall, the OR for ever experiencing a miscarriage for couples where both partners are carriers is 1.93 [95% CI: 1.35, 2.74] compared to non-carrier couples. The OR for couples where one partner is a carrier

should be the square root of this under the additive model, which is 1.39 [95% CI: 1.16, 1.66]. We observe 1.00 [95% CI: 0.96, 1.05], which is a significant deviation (Supplementary Table 12).

Similarly, for DCHR7, we observe 5.34 [2.02-15.7] for couples where both partners are carriers. The square root of this is 2.31 [1.42-3.96] for couples where one partner is a carrier. We observe 1.04 [0.94, 1.15]. Again, this is a significant deviation from the additive model.

The sentence in paragraph 2 on page 13, lines 368-372, of the revised manuscript now reads (changes underlined):

"This indicates that the effect of the c.946-1G>C variant in *DHCR7* on miscarriage is consistent with a recessive model."

Comment #2

The usage of "true positive rate (TPR)" is incorrect, which creates a lot of confusion in paragraphs on page 8. Typically, TPR is also known as sensitivity or recall, which measures the ratio of true positives to all real positives. In this paper, the ratio of (fraction of pLOF or other types of coding variants with deficit of homozygosity) to (fraction of intergenic variants with deficit of homozygosity) is more like positive predictive value (PPV), precision, or (1-false discovery rate), which measures the ratio of true positives to total number of called positives. Please revise in text as well as in legend of Figure 2.

Response to comment #2

Following the reviewer's suggestions, we have changed the presentation to be based on the false discovery rate (FDR), which is the number of false positives divided by the number of positives. As the reviewer points out, this equals 1 – the statistic we referred to as "TPR". We have revised the main text, supplementary information, and the legend of Figure 2 accordingly.

Comment #3

It is unclear how the test for miscarriage rate among carrier couples was done. Was the Fisher's exact test done on the number of couples (experiencing at least one miscarriage vs never) or the number of pregnancy outcomes (miscarriages vs registered births)? If the authors used either, can they also perform test with the other method to see if the result is robust to the choice?

Response to comment #3

We used Fisher's exact test to assess excess miscarriage by comparing the number of mother's who experienced at least one miscarriage versus never between couples where both partners are carriers and control couples where neither partner is a carrier, matched according to birth year and the number of pregnancies.

As per reviewer suggestions, we have now compared the number of pregnancies resulting in miscarriage between mothers from carrier couples and control couples matched on year of birth and number of pregnancies.

We demonstrate that our conclusions are the same based on results from both methods for estimating excess miscarriage.

A supplementary table with the results of Fisher's exact tests on the number of miscarriages between mothers of carrier couples and control couples matched on the year of birth and number of pregnancies has been added to the Supplement (see Supplementary Table 12).

In the main text of the revised manuscript, page 12, first paragraph, lines 333-345 (changes underlined):

"Carrier mothers were at increased risk of ever experiencing a miscarriage if the father was a carrier (N = 57) compared to mothers from non-carrier couples matched on year of birth and number of pregnancies (OR = 1.93 [95% CI: 1.35-2.74], P = 2.4×10^{-4}) (Table 2 and Supplementary Table 12). Consistent with a recessive inheritance pattern, couples where one partner was a carrier were not more likely to experience a miscarriage (OR = 1.0 [95% CI: 0.96-1.05], P = 0.92, N couples = 12,915, N miscarriage = 3,398) (Supplementary Table 12).

The most significant effect on miscarriage was observed for couples carrying pLOF variants in DHCR7 and was significant after correcting for 15 genes being tested (OR = 5.3 [95% CI: 2.0-16], P = $1.9 \times 10^{-4} < 0.05/15$), although we could not show an excess of miscarriage for any other gene individually (Table 2). Couples carrying pLOF variants in the remaining 14 genes also had an excess of miscarriages (OR=1.6[95% CI: 1.3-2.7], P = 0.012, N couples = 119, N miscarriage = 43) (Table 2). We came to the same conclusion by comparing the number of pregnancies that result in miscarriage between mothers from carrier couples and controls (Supplementary Table 12)."

We have also included a description of how Fisher's exact test is used to assess excess miscarriage in the Methods section of the Supplementary Information.

The first paragraph of page 21 of the revised manuscript now reads (changes underlined):

"Differences in miscarriage risk between carrier couples (carrier mother + carrier father, and where one partner is a carrier) versus control couples (non-carrier mother + carrier father) were evaluated using Fisher's exact test. In this study, we assess excess miscarriage both in terms of the number of mothers experiencing at least one miscarriage, and the number of pregnancies resulting in miscarriage between mothers from carrier couples and control couples. Non-carrier control couples were randomly drawn from the group of 61,848 genotyped couples from Iceland where the female partner responded to a routine pregnancy history questionnaire and matched on age and number of pregnancies (1:100 nearest neighbor matching with replacement)."

Comment #4

I agree with the authors' assessment that given the current sample size, power is limited. It would improve the paper to perform some power analysis to quantitatively evaluate the power of detecting variants with homozygosity deficit with the current criteria used.

Response to comment #4

We have added a power analysis to the paper that assesses the impact of allele frequency and sample size changes on the power to detect a deficit of homozygosity.

The results of the power analysis are shown in Supplementary Figure 6. which is referred to at the end of the last paragraph in the "Gene set over-representation analysis" subsection, Page 11, last paragraph, lines 320-325, now read (changes underlined):

"This shows that we only have statistical power to detect the subset of such variants in the combined set of 1.52 million individuals with a MAF of at least 0.2% (pLOF: MAF ≥ 0.18% corresponding to an expected homozygous count of 5, moderate impact variants: MAF ≥ 0.23% corresponding to an expected homozygous count of 8) (Supplementary Fig. 6). It has been suggested that the majority of recessive lethal variants are very rare and likely rarer than those identified in the current study[46]."

The Supplementary Methods now include a description of the Power analysis performed. See subsection "Power analysis" on Page 22:

"For the power analysis, we used a two-sample proportional test. We assumed that the true homozygote frequency in the population was 10% of its expected frequency. We estimated the sample size required to detect a deficit of homozygosity with 80% power (significance level = 0.05), as well as the power to detect the effect of a deficit of homozygosity on minor allele frequencies between 0 and 1.6%. We used the R function stats::power.prop.test to perform the power analysis (sig.level = 0.05, power = 0.80, p1 = expected frequency of homozygous genotype, p2 = 0.1 * p1)."

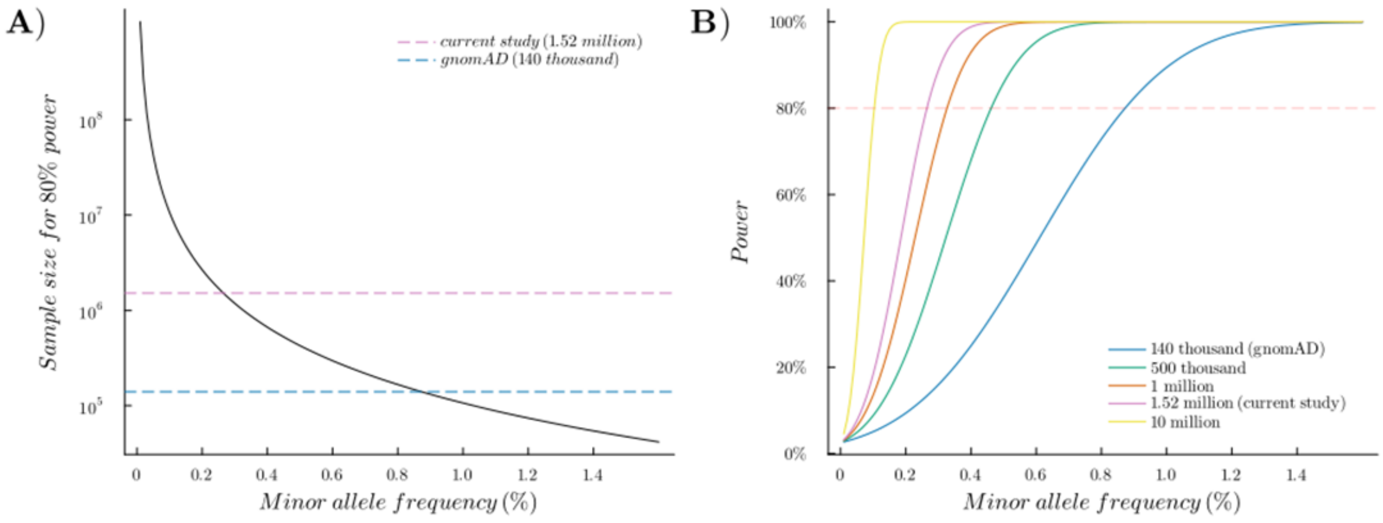

S. Figure 6. Power analysis. A) sample size to detect deficit of homozygosity at 80% power at a significance level of 0.05, and B) the power to detect an effect at minor allele frequencies between 0 and 1.6% for various sample sizes (dashed red line indicates a power of 80%).

Comment #5

The authors briefly discussed the total number of genes with homozygous knockouts found in human population so far and provided a summary in Supplementary Data S3. These results, while useful, may be mis-interpreted as evidence for being non-pathogenic or compatible with survival, although some of them could still cause severe condition or even lethality due to incomplete penetrance. It would be helpful if the authors can explicitly discuss the utility and limitations of these results in Discussion to guide application in clinical settings.

Response to comment #5

We have now added a paragraph regarding genes with homozygous knockouts found in human populations to the Discussion section. We believe that the rarity of this extreme genotype will be valuable to the community as it has previously been useful to access genotype counts from large public databases and publications.

Page 15, second last paragraph, lines 435-443, now reads (changes underlined):

“In addition to detecting genes with a deficit of homozygotes, we identified 2,671 genes with observed homozygotes for pLOFs, most of which involve two or more individuals (1,722/2,671 = 66.3%) in the set of 1.52 M individuals. Some of the annotated pLOF variants may not be true loss-of-function variants meaning that true loss-of-function homozygotes could still not be viable. Also, our analysis will only identify deficit of genes that cause loss-of-function homozygotes to be absent from the general population, and the detection of homozygotes for pLOFs suggests that biallelic loss-of-function mutations of these genotypes are not lethal before adult age. However, we cannot exclude the possibility that some of these genotypes would have severe phenotypic effects (Supplementary Discussion).”

Reviewer 2: Minor comments

Comment #1

The current observations can be ordered differently to make the logic connection easier to follow. In my opinion, association of the identified variants to reduced expression can be presented right after identification of the variants with homozygosity deficit, as a sanity check of the predicted impacts. Then the comparison with Mendelian disease genes and essential genes can be presented and lead to the hypothesis that some of these variants may cause pre-natal lethality instead of post-natal disorder, which is further tested by the analysis of miscarriage rate in carrier couples.

Response to comment #1

Following the reviewer's suggestion, we have moved the section "Effect of variants with a deficit of homozygosity on gene expression" immediately following the section "Deficit of homozygosity", and the section “Gene set over-representation analysis” is now presented before the assessment of excess miscarriage.

As a result, the following text has been removed from the section "Gene set over-representation analysis" on page 10 of the revised manuscript.

“In this study, it was only possible to test for excess miscarriage in the Icelandic population, and only for variants carried by couples with a substantial number of documented pregnancies. Thus, in most cases, the number of heterozygous couples is too small to allow detection of excess miscarriage. In these cases.”.

In the "Gene set over-representation analysis" section on page 11 of the revised manuscript, the following text has been added to the second paragraph, lines 313-314.

“Thus, pLOF variants in genes with deficit of homozygosity may cause pre-natal lethality rather than a post-natal disorder. “

Comment #2

In lines 228-229, the method for calculating p-value (i.e., assuming Poisson distribution) should be included in the parentheses in addition to the Bonferroni p-value threshold.

Response to comment #2

The statement “assuming Poisson distribution” has been included in the parentheses in addition to the Bonferroni P-value threshold on page 8, paragraph 2, lines 234-235, and page 9, paragraph 1, line 244.

Comment #3

Perhaps I am not following but I find it confusing that the numbers in the text sometimes do not match those in the tables. For example, in lines 245-247, it reads “11 are located in genes that have not been reported to cause a Mendelian condition… The remaining 14 genes are reported to have variants causing a Mendelian condition…” but in Table 3, 10 and 9 genes with expected homozygotes>=5 are in OMIM AR genes versus not, respectively. Later, in line 359-360, it says “Sequence variants in 12 of the 25 genes causes Mendelian disease under a recessive mode…” I’m very confused by the small differences in numbers.

Response to comment #3

The results of the gene set overrepresentation analysis presented in Table 2 of the revised manuscript include genes identified through the gene-based burden test (i.e., 19 geneLOFs). As a result, the numbers in the text discussing individually the 25 genes identified through any test we performed (single variant, and geneLOF) may not correspond to those in the table when limiting to the 19 genes identified through geneLOFs. We only used the 19 genes detected through geneLOFs in the gene overrepresentation analysis since the unit of our test is the gene but not the variant when comparing to genes with recessive Mendelian diseases, or genes lethal when knocked out in mice and cell experiments.

Specifically, we identified 25 genes with a deficit of homozygosity either through burden or single variant test; 19 involving pLOFs (10 linked to mendelian disease; 9 recessive, 1 dominant), and six involving moderate impact variants (4 linked to Mendelian disease; 3 recessive, 1 dominant).

When characterizing all identified genes with a deficit of homozygotes:

Lines 245-247 refer to the proportion of 25 genes with homozygous deficit variants (either pLOF or moderate impact variants) associated with Mendelian disease, regardless of inheritance mode (14 of the 25 genes are linked to Mendelian disease).

In a different chapter, lines 359-360 we refer to the fraction of the 25 genes harboring homozygous deficit variants in genes linked to recessive Mendelian disease (14 homozygous deficit genes are linked to Mendelian disease, 12 recessive, and 2 dominant).

On page 9, paragraph 2, line 256, we have now included more context about genes harboring homozygous deficit variants linked with Mendelian disease (changes underlined):

"The remaining 14 genes are reported to have variants causing a Mendelian condition (12 under a recessive mode of inheritance, two under a dominant mode)".

We now specify that the test unit of the gene over-representation analysis is the gene.

Page 21, second paragraph, of the Supplementary Methods now reads (changes underlined):

"As the unit of the test is the gene, we used the 1,258 geneLOFs with five or more expected homozygotes in the meta-analysis of all 1.52 million individuals."

The header of Table 2 in the revised manuscript now reads:

"Table 2: Gene set over-representation analysis of genes with deficit detected through a gene-based test of pLOFs (geneLOFs) ".

Comment #4

Similarly, in lines 312-314, it says "based on being essential for growth of human cell lines, 13 genes…" but in Table 3, 11 genes with expected homozygotes>=5 are essential in human cell line.

Response to comment #4

In the gene set over-representation analysis presented in Table 2 of the revised manuscript only 19 geneLOFs are tested for the enrichment of genes essential for the growth of human cell lines. Again, when performing the test, we only used the 19 genes detected through geneLOFs in the gene overrepresentation analysis since the unit of our test is the gene but not the variant when comparing to genes with recessive Mendelian diseases, or genes lethal when knocked out in mice and cell experiments.

Please see the response to comment #3 above for more information.

For clarification, the header of Table 2 in the revised manuscript now reads “Table 2: Gene set over-representation analysis of geneLOFs”.

Reviewer 3 (Remarks to the Author)

Here the authors present a unique opportunity to detect rare recessive (and potentially lethal) variation. The authors do this by combining large genotyping and sequencing datasets. They proceed to identify multiple genes that could potentially be linked to embryonic lethality and approximately 1000 genes with previously undetected predicted loss of function variants. The paper is concise and reads well for the intended audience of the journal. I think with additional interrogation of the statistic and variants it will provide strong evidence for previously unknown lethal variants.

Below I list major comments and a number of minor comments detailed below.

Reviewer 3: Major comments

Comment #1

Given the amount of data that is being imputed and merged here I am wondering what the final set of variants used for each of the analyses was? It seems like there was a population specific set of variants and maybe a combined set? Additionally, was there any QC done on the final dataset once the whole-genome sequence was imputed into the chip-typed individuals?

Response to comment #1

We based our study on variant calling and imputation for Denmark, Norway, and Sweden populations jointly but separately for Iceland, Finland, and the UK populations. The expected homozygote count for each population (i.e., each country) was calculated independently. The total number of expected homozygotes equals the sum of the expected homozygotes in each population. In the combined set of 1.52 million individuals, the deficit of observed homozygotes was then tested for variants with an expected homozygote count of at least 0.5. We have added a supplementary Table (Supplementary Table 3) that includes the number of sequence variants tested in each population stratified by annotation.

As part of our quality control (QC), we restricted our analysis to autosomal variants within Tier 1 regions on the basis of Genome in a Bottle (GiaB). We also analyzed observed to

expected ratios of homozygote counts to identify regions of the genome overrepresented among variants that did not follow Hardy-Weinberg expectations. As a result, we also excluded variants that are located in centromeres, telomeres, and segmental duplication regions. These regions are difficult to map with short-read sequencing technologies and are more likely to deviate from Hardy-Weinberg expectations (see "Identifying a deficit in the number of observed homozygotes" under "Supplementary methods").

Our greatest worry when it comes to QC is artificial deficit of homozygotes because of genotyping or imputation artifacts. Our imputations are haplotype-based rather than genotype based, which makes these types of artifacts less likely. Using the frequency of variants being at deficit of homozygosity among intergenic variants as a reference does address this issue because imputation artifacts should not preferentially affect loss-of-function variants over intergenic variants.

For the reader's convenience, in the supplementary information we have revised the subsections „Imputation" and "Identification of a deficit in the number of observed homozygotes":

Page 17, first paragraph, now read (changes underlined):

"<u>Because our imputations are based on haplotype rather than genotype, we are less likely to encounter artificial deficits in homozygotes as a result of genotyping or imputation errors.</u> Importantly, given the two phased haplotypes of each individual, the imputation of the individual's two haplotypes was performed independently which leads to less dependence between the imputed alleles than when genotypes are imputed from genotypic data."

We note that according to the suggestion of reviewer #2 (see the response to comment #2), we have changed the presentation of the estimate of homozygous deficit to be based on the false discovery rate (FDR). Page 19, first paragraph, now reads (changes underlined):

"We then estimate the false discovery rate (FDR) among the protein-altering sequence variants with the excess of variants with deficit of homozygosity among the protein-altering sequence variants compared to the intergenic sequence variants relative to the total number of protein-altering sequence variants with deficit of homozygosity: $FDR = 1 - \frac{H-L}{H}$. <u>Using</u>

<u>the frequency of variants at deficits of homozygosity among intergenic variants as a reference does address the issue of artificial deficit of homozygotes caused by genotyping or imputation artifacts since imputation artifacts should not preferentially affect protein-altering variants over intergenic variants.</u>"

Comment #2

Have the authors thought about how imputation and sequencing error could be factored into the variant level analyses? I think this is especially important given the very low frequency variants that this work is aimed at identifying.

Response to comment #2

The current study used genetic information from WGS a large set of individuals in each population (N Iceland = 46,173; N UK = 131,958; N Denmark + Norway + Sweden = 8,360 + 3,301 + 3,579 = total of 15,240; N Finland = 3,775). The total number of individuals WGS from these six populations is 197,146.

The rarest variants tested in this study are common enough to be shared by enough sequenced carriers to allow accurate imputation. The low-end MAF of the single variants considered in the current study is ~ 0.06 %, corresponding to 0.5 expected homozygotes in the combined population of 1.52 million. In the Icelandic population, a variant of this frequency is expected to be observed in approximately 50 whole-genome sequenced heterozygous carriers, allowing accurate imputation. Previously, we demonstrated that five sequenced carriers are sufficient to impute sequence variants accurately (Gudbjartsson et al., 2015; PMID: 25807282), and we have validated whole-genome sequencing and imputation methods using Sanger sequencing on individuals predicted to be homozygous for rare pLOF variants (Sulem et al., 2015; PMID: 25807282).

In response to this comment, we have added a column to Supplementary Table 2 showing the expected number of carriers in the set of WGS individuals for each MAF bin.

The Supplementary Information has been revised to include a discussion regarding imputation of rare variants in the subsection "Distribution and medical impact of homozygous deficit variants".

The first paragraph of page 24 of the Supplementary information now reads as follows (changes underlined):

"The current study is based on the detection of variants through whole-genome sequencing and imputation into a larger set of chip-typed individuals. The rarest variants tested are common enough to be shared by a sufficient number of sequenced carriers to allow accurate imputation. As we have demonstrated in previous studies, the identification of five sequenced carriers is adequate for accurate imputation[1,45]."

Comment #2a

Another thought is what is the mappability like in each of these regions? I don't think that the results here are caused by poor mappability, given the other layers of support, but it would be good to check.

Response to comment #2a

In our analysis, we only took into account variants in high-confidence Tier 1 regions of the genome that are well-mappable by short-read sequencing (Genome in a Bottle consortium). Further, we excluded regions within Tier 1 that are tandem repeats, extreme centromeric, and extreme telomeric. These restrictions amount to only analyzing regions that are well mappable.

For more information, see the section "Identification of a deficit in the number of observed homozygotes" in the Supplementary information on page 17.

Comment #3

How are intergenic regions found? I think this is especially important given that the TPR metric is based on these intergenic regions being as close to neutral as possible.

Response to comment #3

Variants located more than 5 kb outside of RefSeq-annotated genic regions were defined as intergenic using the Variant Effect Predictor (VEP).

The presence of variants under negative selection in the recessive state among the variants we classify as intergenic will increase the number of sequence variants with deficit of homozygosity among them. This will increase our estimate of the fraction of variants with deficit of homozygosity under the null and will in turn reduce our power to detect loss-of-function variants at deficit of homozygosity. However, this would not lead to false positive signals.

This information can be found in the Supplementary Information under the Methods section titled "Identification of a deficit in the number of observed homozygotes" on page 17 (changes underlined).

“We used Variant Effect Predictor (VEP)[24] to assess the functional impact of sequence variants. We assessed homozygote count for intergenic variants (located in intergenic regions more than 5 kb from a RefSeq annotated genic region)”.

Comment #3a

I might suggest authors try making a couple sets of these intergenic variants to account for the effect of linked selection. For example, the putatively neutral sites have different genetic distances to the nearest gene (e.g. 0cM, 0.2cM,0.4cM…1cM) to calibrate the TPR statistic that is being used to identify strong directional selection in the putative lethals.

Response to comment #3a

Based on the reviewer's suggestion, we defined sets of intergenic variants located at different distances from the ~20K genes (as defined by RefSeq). In addition to the distance of 5 kb we

initially used, we also tried regions based on 50 kb, 100 kb, 250 kb, and 500 kb outside of annotated genic regions to account for hitchhiking effects due to linked selection (see Supplementary Table 15). For the sets of variants considered, the small differences in homozygous deficit rates between the sets do not result in substantial fluctuations in the FDR (see Supplementary Fig. 7). The set of intergenic variants defined by 500 kb exclusion is much smaller and contains much fewer variants than the set defined by the 5 kb exclusion (875 K variants compared to 6.1 million variants) and, therefore, results in much noisier results.

Supplementary Table 15 (See also the separate Excel file with all supplementary tables).

S. Table : Sets of intergenic variants located 5 kb, 50 kb, 100 kb, 250 kb, and 500 kb outside of annotated genic regions

	BINS EXPECTED HOMOZYGOTES		MEAN					VARIANTS (N)					HOMOZYGOUS DEFICIT (N)					HOMOZYGOUS DEFICIT (%)				
	BIN	~ MAF (%)	5 KB	50 KB	100 KB	250 KB	500 KB	5 KB	50 KB	100 KB	250 KB	500 KB	5 KB	50 KB	100 KB	250 KB	500 KB	5 KB	50 KB	100 KB	250 KB	500 KB
	[0.5, 1)	0.07%	0.72	0.72	0.72	0.72	0.72	507,029	331,378	258,618	144,271	60,590	167,029	109,710	85,761	47,855	20,280	32.9%	33.1%	33.2%	33.2%	33.5%
	[1, 2)	0.10%	1.4	1.4	1.4	1.4	1.4	456,467	302,263	237,652	133,604	55,545	63,294	42,135	33,171	18,649	7,663	13.9%	13.9%	14.0%	14.0%	13.8%
	[2, 3)	0.13%	2.5	2.5	2.5	2.5	2.5	243,062	162,671	128,508	72,269	30,010	9,370	6,131	4,815	2,699	1,040	3.9%	3.8%	3.7%	3.7%	3.5%
	[3, 5)	0.16%	3.9	3.9	3.9	3.9	3.9	280,215	190,552	151,237	86,472	36,716	2,484	1,641	1,265	754	364	0.89%	0.86%	0.84%	0.87%	0.99%
	[5, 8)	0.20%	6.4	6.4	6.4	6.4	6.4	237,980	163,378	129,915	74,101	31,413	199	141	114	64	32	0.084%	0.086%	0.088%	0.086%	0.102%
	[8, 13)	0.26%	10	10	10	10	10	227,346	157,171	125,346	71,974	30,228	10	8	7	4	2	0.0044%	0.0051%	0.0056%	0.0056%	0.0066%
	[13, 250)	0.70%	74	75	76	76	76	1,118,199	807,092	655,878	385,742	163,279	5	3	2	0	0	0.0004%	0.0004%	0.0003%	0.0000%	0.0000%
	[250, 1000)	1.9%	538	539	539	537	535	428,370	322,929	264,964	156,550	67,163	0	0	0	0	0	0.0000%	0.0000%	0.0000%	0.0000%	0.0000%
	[1000, 10 K)	5.2%	4,071	4,068	4,055	4,035	4,047	775,059	592,719	481,565	277,637	118,768	0	0	0	0	0	0.0000%	0.0000%	0.0000%	0.0000%	0.0000%
	[10 K, 100 K)	16.5%	41,598	41,903	42,067	42,186	42,300	1,126,231	873,292	704,840	395,123	165,216	0	0	0	0	0	0.0000%	0.0000%	0.0000%	0.0000%	0.0000%
	[100 K, 250 K)	32.9%	164,019	164,152	164,398	164,117	163,213	544,161	438,278	360,104	209,200	89,604	0	0	0	0	0	0.0000%	0.0000%	0.0000%	0.0000%	0.0000%
	[250 K, 500 K]	44.0%	294,687	295,055	295,189	295,697	294,937	163,353	132,526	108,508	62,982	26,996	0	0	0	0	0	0.0000%	0.0000%	0.0000%	0.0000%	0.0000%
Total	—	—	—	—	—	—	—	6,107,472	4,474,249	3,607,135	2,069,925	875,528	242,391	159,769	125,135	70,025	29,381	—	—	—	—	—

BIN: bins of expected homozygous count
~ MAF(%) : approximate mean allele frequency in a population of 1.52 million individuals
MEAN : mean expected homozygous count
Total: Sum of the number of variants

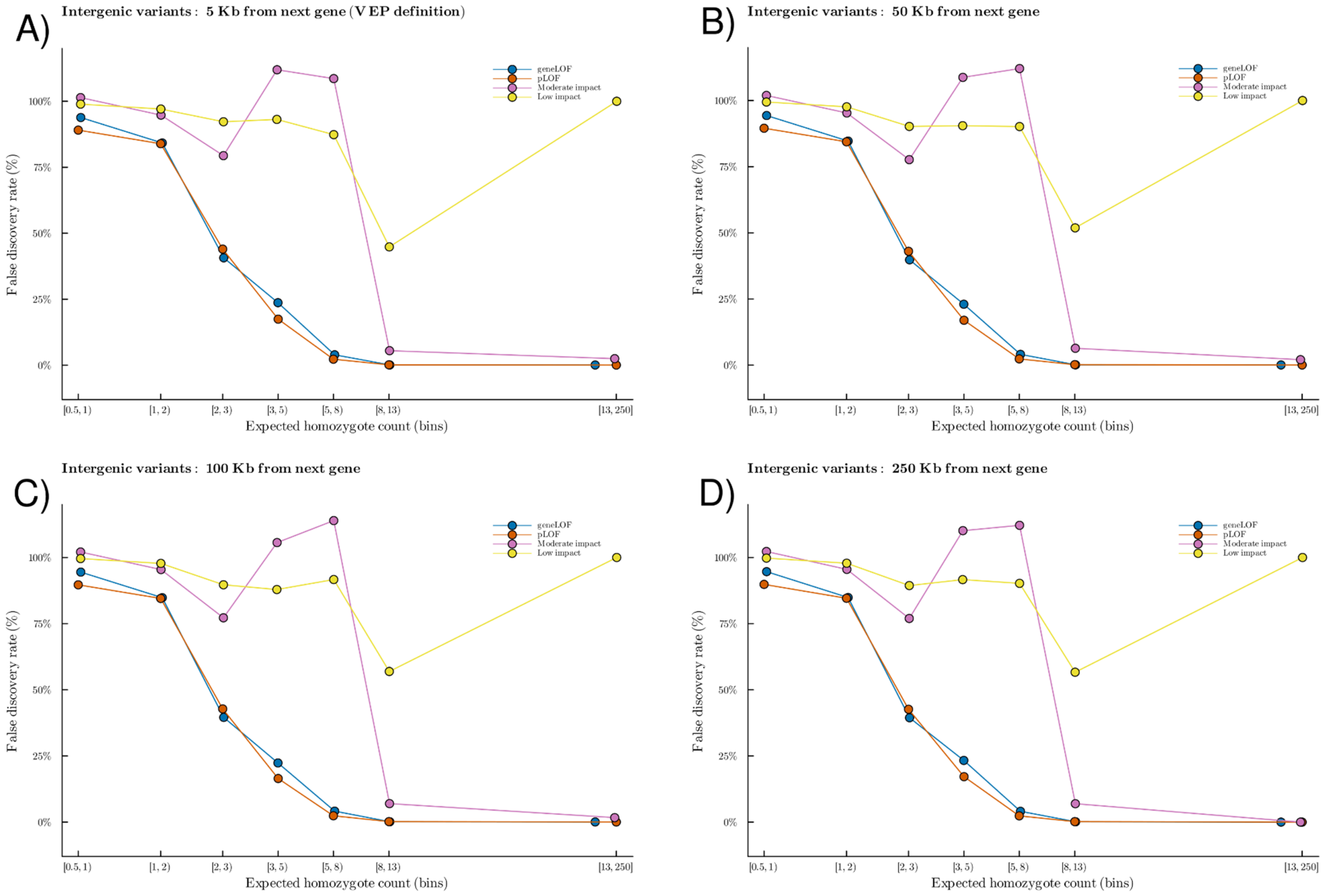

S. Figure 7: False discovery rate (FDR) for deficit of homozygosity relative to intergenic variants in the combined set of 1.52 million individuals of North-Western European descent (Denmark, Finland, Iceland, Norway, Sweden, and the UK) calculated from different sets of intergenic variants located A) 5 kb (as defined by VEP), B) 50 kb, C) 100 kb, and D) 250 kb outside of RefSeq annotated genic regions.

Comment # 3b

Also, could the authors add a section about the intergenic variation to the methods where it is calibrated based on distance from genes or other highly conserved regions?

Response to comment #3b

We have added a paragraph describing how we defined the intergenic regions to the section "Identification of a deficit in the number of observed homozygotes" in the Supplementary methods.

It is important to note that our analysis excluded highly correlated variants between impact classes in order to take linked selection into account. This important point, however, was not mentioned in the main text or supplementary methods. Specifically, we removed moderate-impact variants highly correlated ($R^2 > 0.8$) with high-impact variants from the moderate-impact class, low-impact variants highly correlated with moderate or high-impact variants from the low-impact class, as well as intergenic variants highly correlated with moderate, high, or low-impact variants from the intergenic class.

On page 19, a new paragraph has been added to the sub-section "Identification of a deficit in the number of observed homozygotes" Supplementary Methods:

"To account for hitchhiking effects due to linked selection, we excluded highly correlated variants between impact classes and additionally defined sets of intergenic variants with different exclusion regions outside of RefSeq annotated genes to calibrate the FDR. Specifically, we removed moderate-impact variants that are highly correlated (R2 > 0.8) with high-impact variants from the moderate-impact class, low-impact variants that are highly correlated with moderate or high-impact variants from the low-impact class, and intergenic variants that are highly correlated with moderate, high, or low-impact variants from the intergenic class. Additionally, we defined sets of intergenic variants located 5 kb, 50 kb, 100 kb, 250 kb, and 500 kb outside of annotated genic regions (Supplementary Table 15). There were no substantial fluctuations in the FDR as a result of the choice of intergenic variant sets (Supplementary Figure 7). For further analysis we used intergenic variants located 5 kb outside of annotated genic regions which is the definition used by VEP[24]."

Comment #4

When thinking about Figure 2, I am wondering if the authors could explore what is going on with the moderate impact variants in the [3,5) and [5,8) bins? There should be a decent number of moderate impact variants (at least more than pLOF and geneLOF), so I am wondering why the TPR is depleted for these two bins then jumps up dramatically for [8,13)?

Response to comment #4

A drop in the TPR statistic in the [3,5) and [5,8) bins is consistent with being the result of fluctuations in the fraction of variants at deficit (Figure 2.). There are 4,806 moderate impact variants in the [3,5) bin. Of these, 38 were at deficit or 0.79%. Assuming that the variants are independent, the 95% CI for this fraction is 0.56% – 1.07%. Ignoring the uncertainty in the estimated fraction of intergenic variants at deficit of homozygosity, the 95% CI for the TPR would then range from -57% up to 17%. Similarly, under these assumptions, the 95% CI for the [5,8) bin would range from -100% up to 59%. Obviously, the variants are not independent, and the variability in the fraction estimated from the matched intergenic variants should be taken into account, both of which would increase the size of the CIs slightly.

Comment #5

I had a couple questions about the λ statistic for identifying the deficit of homozygotes. I will frame questions specifically in the context of Table 1.

Comment #5a

My first question is whether the p-value corrected for multiple testing? I don't believe that it is. Can authors comment on this and maybe add a sentence to the caption and/or methods?

Response to comment #5a

The p-value contained in Table 1 is not corrected for multiple testing. We do not use this p-value to determine statistical significance but rather the TPR of 90% (corresponding to an FDR of 10% in the updated presentation). To simplify the presentation of the results in light of this, we have removed the p-values in the main Table 1, while we still discuss these p-values in the text to explore the effects of using the FDR method rather than Bonferroni correction for multiple testing:

For single variants, the text reads:

"In comparison, using Bonferroni correction for multiple testing, five variants had a significant deficit for homozygosity for pLOF ($P < 0.05/1{,}736 = 2.9\times10^{-5}$, assuming Poisson distribution) and two for moderate impact variants ($P < 0.05/47{,}429 = 1.1\times10^{-6}$, assuming Poisson distribution) (Supplementary Table 2)."

And for geneLOFs burden:

"Five or more homozygous individuals were expected for 1,258 geneLOFs and nineteen of these genes had a deficit of homozygosity (Tables 1). If we determined significance based on deviation from HWE and use Bonferroni correction for multiple testing ($P < 0.05/1{,}258 = 4 \times 10^{-5}$, assuming Poisson distribution), ten genes had a significant deficit of homozygosity (Supplementary Table 4)."

Comment #5b

Second question, is there a correction for structure for pooled sample? One thing that I worry about when combining all this data together, then computing the p-value, is how structure could be affecting the statistic. My interpretation of the methods section led me to think that there is not a correction. We know from previous papers that there is structure within and across European populations. So, perhaps the p-value should be computed within each population if there is no correction? Or maybe the authors could add a frequency-based correction?

Response to comment #5b

It is a peculiar consequence of our focus on the deficit of homozygosity that population structure will tend to increase the number of homozygotes and thus may potentially hide signals rather than create false positives. Also, our use of intergenic variants as controls should control for structure between and within populations. This is because, conditional on the frequency of variants, the effects on structure on them should not depend on their annotation under the null hypothesis of the variant not being under negative selection.

Also, see Reviewer 3 Comment 1; for information on testing in each population.

Comment #6

If we think of a site where I expect 5 homozygotes and observe 0 in a population, it seems like that could happen about 1% of the time (~e-5/0!). I am wondering if the authors could spot check a few of the genes (e.g., CCDC59) by looking at reads from a handful of sequenced individuals in IGV? Do the reads support the site being fixed for the other homozygote or heterozygous?

Response to comment #6

We manually assessed different regions, especially interested in the ones with indels: The *AGK* chr7:141649323 TAAC duplication, *MVD* chr16:88663006 C to T substitution, *CCDC59* chr12:82354490 TTACTTGT deletion, and the *RPAP2* chr1:92333464 GAGTA deletion.

We examined BAM files from over 20 individuals of each genotype from both heterozygous carriers and non-carriers. The data in the BAM files was consistent with the reported genotypes in all cases. In particular, we always observed multiple copies of the reference allele in heterozygotes. Below are screenshots of BAM files encompassing each genotype. Each figure illustrates one heterozygous carrier and non-carrier sequence reads for each of these four variants.

The second paragraph of page 21 of the Supplementary information now reads as follows (changes underlined):

"We manually assessed BAM files of different regions of variants with homozygous deficit, with particular interest in those with indels. These included the AGK chr7:141649323 TAAC duplication, the MVD chr16:88663006 C to T substitution, the CCDC59 chr12:82354490 TTACTTGT deletion, and the RPAP2 chr1:92333464 GAGTA deletion (Supplementary figures 8-11).

We examined the BAM files of more than 20 individuals of each genotype, including heterozygotes and non-carriers, to confirm that the data in the BAM files corresponded to the reported genotypes in all cases. The reference allele was observed to have multiple copies in heterozygotes in all cases."

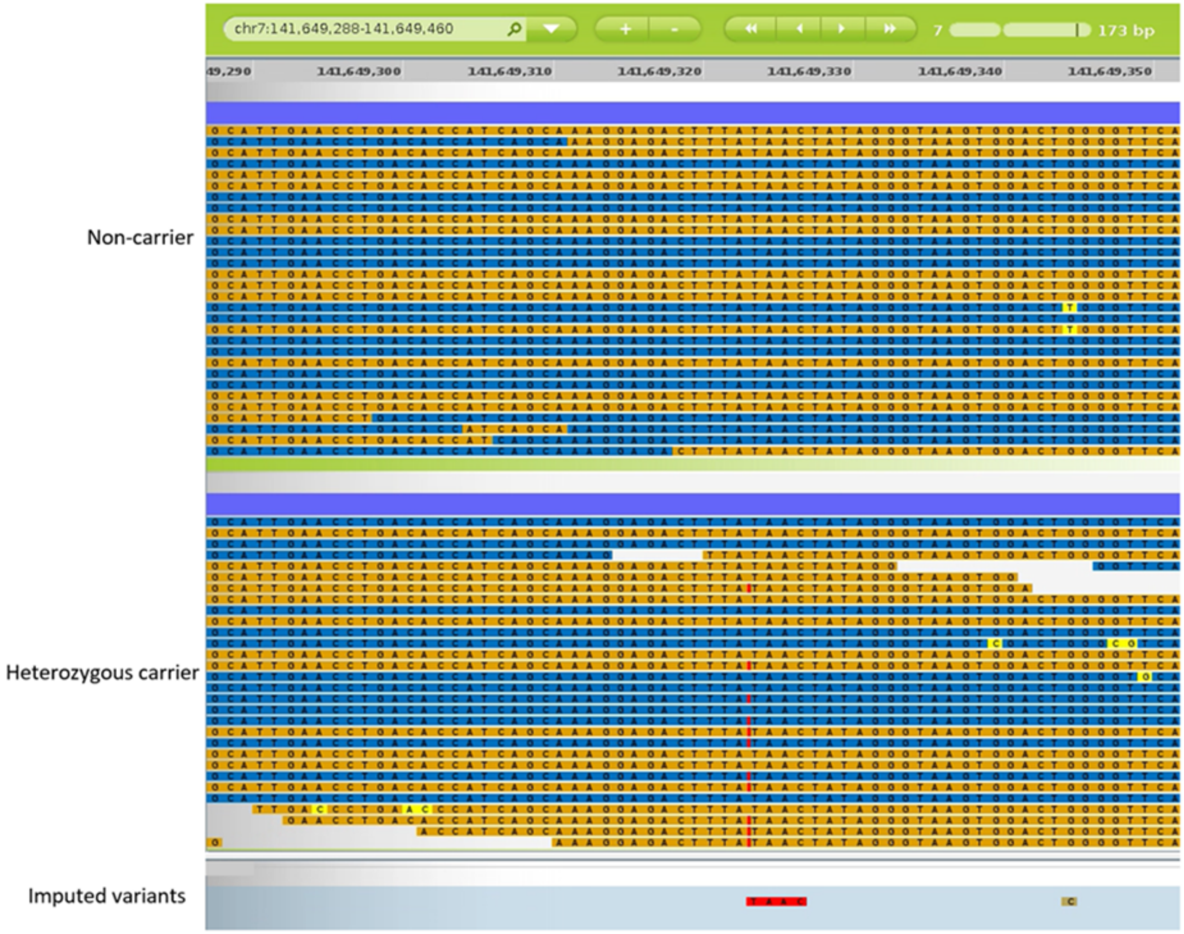

S. Figure 8. AGK chr7:141649323 TAAC duplication: The chromosomal position (chr7:141649323) of the TAAC duplication in AGK in a heterozygous carrier (middle track) and a non-carrier (top track). The TAAC duplication is noted in red and marked with an arrow in the figure. The bottom track shows variant calls for all variants that we impute in this region, including the TAAC duplication (Yellow and blue reads are in opposite directions).

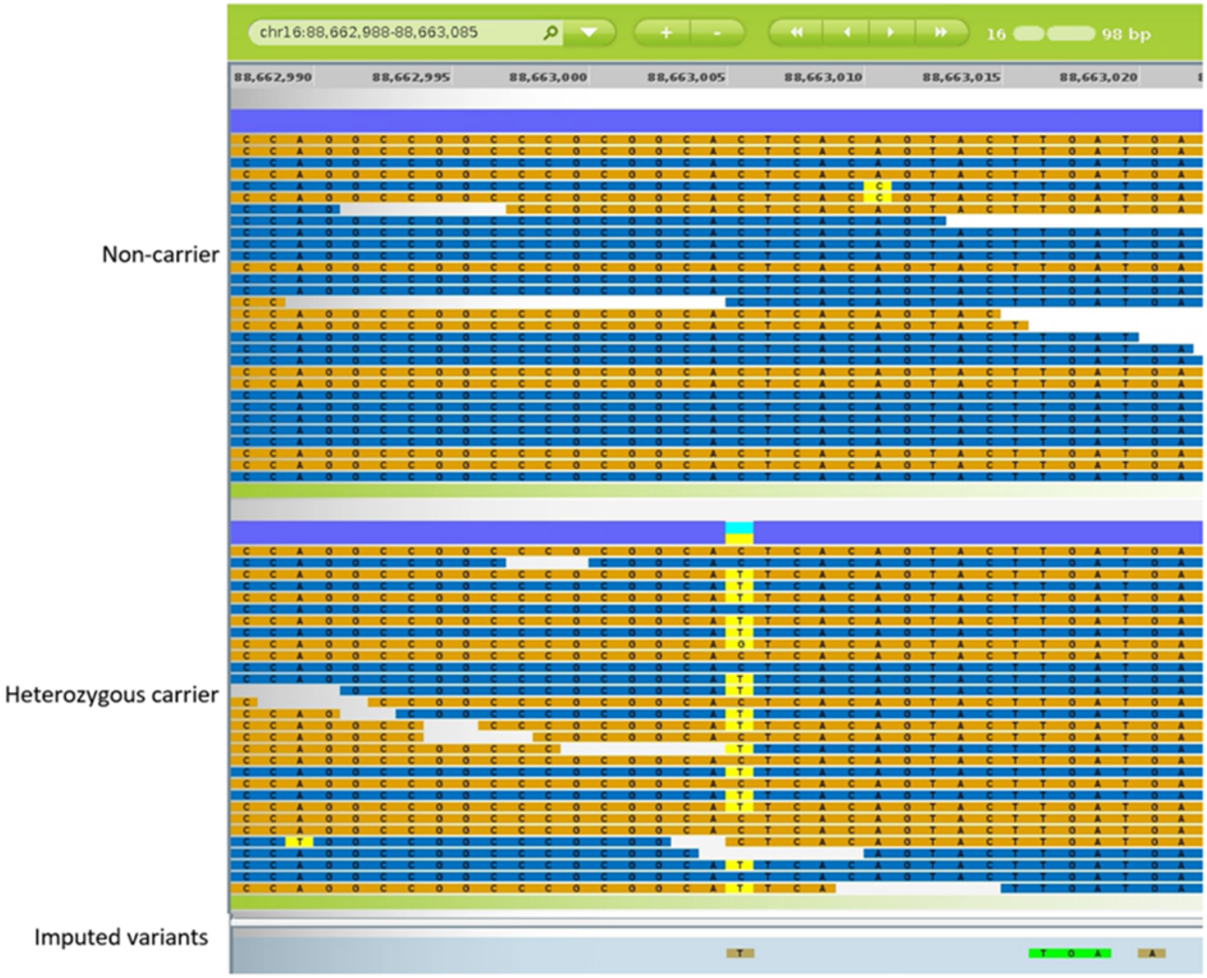

S. Figure 9. MVD chr16:88663006 C-T substitution: The chromosomal position (chr16:88663006) of the C-T substitution in MVD in a heterozygous carrier (middle track) and a non-carrier (top track). The C-T substitution is noted in yellow and marked with an arrow. The bottom track shows variant calls for all variants that we impute in this region, including the C-T substitution.

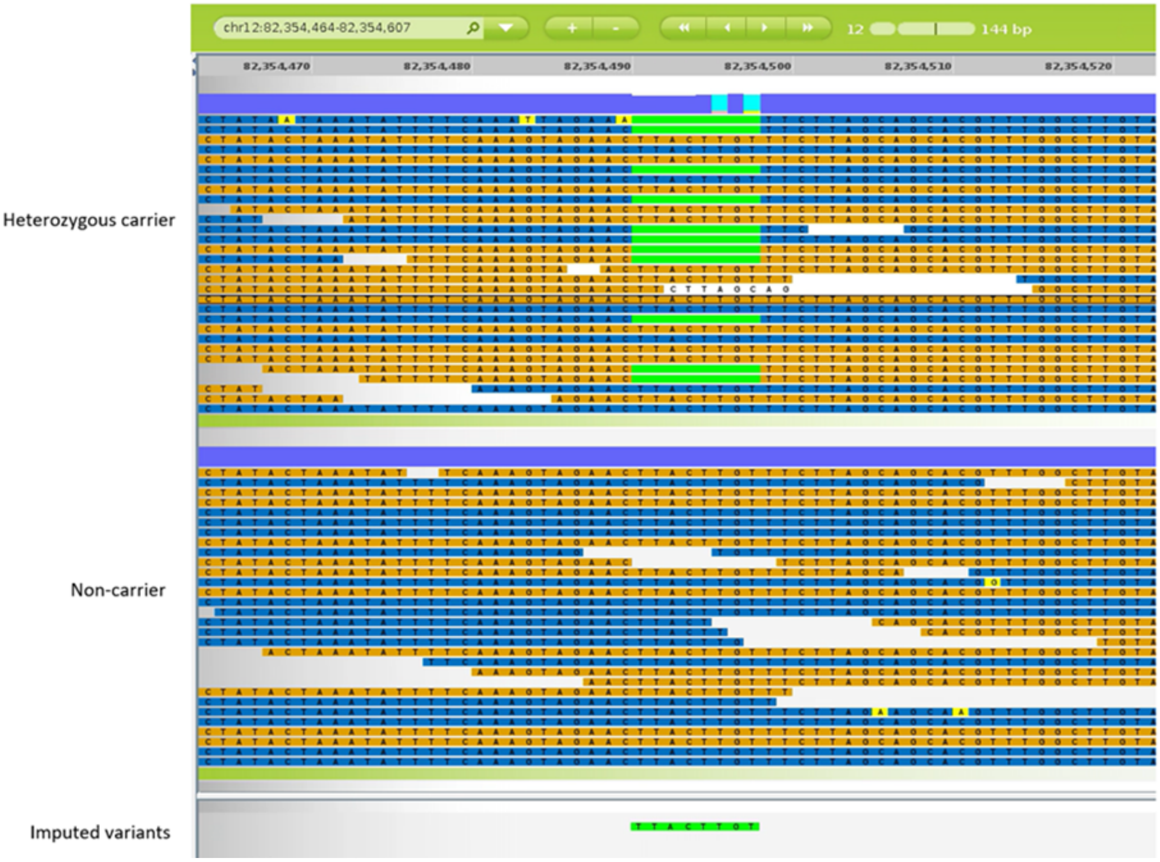

S. Figure 10. CCDC59 chr12:82354490 TTACTTGT deletion: The chromosomal position (chr12:82354490) of the TTACTTGT deletion in CCDC59 in a heterozygous carrier (top track) and a non-carrier (middle track). The TTACTTGT deletion is noted in green and marked with an arrow. The bottom track shows variant calls for all variants that we impute in this region, including the TTACTTGT deletion.

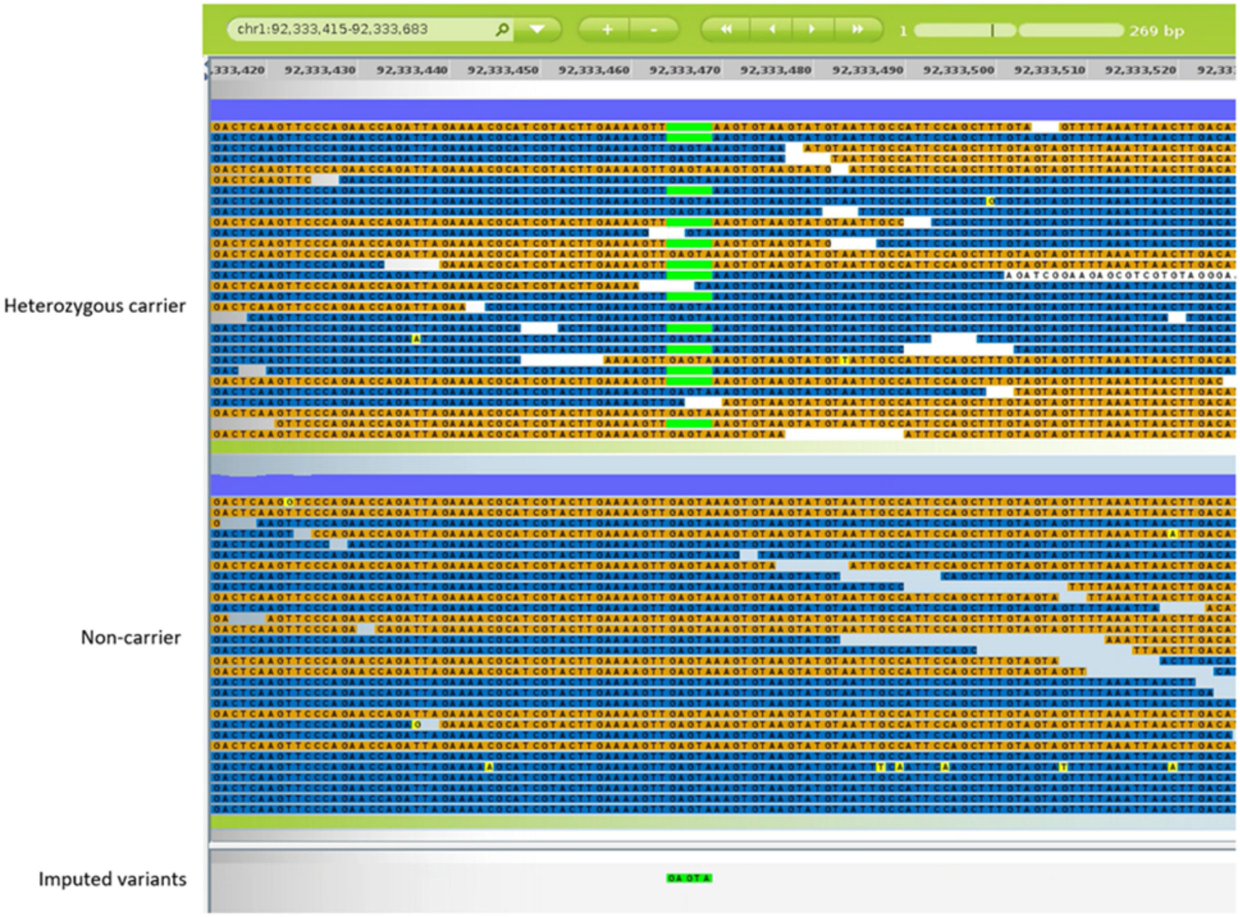

S. Figure 11. RPAP2 chr1:92333464 GAGTA deletion: The chromosomal position (chr1:92333464) of the GAGTA deletion in RPAP2 in a heterozygous carrier (top track) and a non-carrier (middle track). The GAGTA deletion is noted in green and marked with an arrow. The bottom track shows variant calls for all variants that we impute in this region, including the GAGTA deletion.

Comment #7

Could the authors elaborate on exactly which individuals are being used for the miscarriage among carrier couples analyses? In the supplementary methods, the authors say that the data comes from Icelandic women who participated in a cohort study for screening breast and cervix cancer at the Icelandic Cancer Center. Did any women used in the analyses have or develop cancer or were the carrier couples only from the control group? My thought would be that one would not be using carrier couples where the mother developed cancer because that could potentially explain the miscarriage rate more so than the carrier status.

Response to comment #7

For the miscarriage among carrier couples analysis, Icelandic women who completed a pregnancy history questionnaire in connection with routine cervix and breast cancer screenings and their male partners were analyzed. As a result, this set is representative of Icelandic women of childbearing age during this time period and is not biased in favor of cancer above the background rate (Supplementary Table 16).

As a further point of clarification, the couples where one partner was a carrier were not more likely to experience a miscarriage than non-carrier couples, which was designed to control for confounding factors (Supplementary Table 12).

Page 20, the first paragraph of the Supplementary Methods, now reads (changes underlined):

"We looked for records of miscarriage among 61,848 genotyped couples from Iceland where the female partner completed a pregnancy history questionnaire at the Cancer Detection Clinic of the Icelandic Cancer Society, carried out in connection with routine screening for cancers of the cervix and breast between 1964 and 1994 (Supplementary Table 16)"

Supplementary Table 16 (See also the separate Excel file with all supplementary tables).

S. Table : Cancer rate among 87,078 Icelandic women who answered a routine pregnancy history questionnaire in a healthcare setting between 1964 and 1994.

	INDIVIDUALS (N)	GENOTYPED (%)	BREAST CANCER (%)	CERVICAL CANCER (%)
**BIRTH COHORTS - MISCARRIAGE EVER**				
1879-1900	99	1 (1.01%)	8 (8.08%)	4 (4.04%)
1900-1925	4,676	1,638 (35.03%)	382 (8.17%)	50 (1.07%)
1925-1950	9,829	6,808 (69.26%)	1,033 (10.51%)	79 (0.80%)
1950-1977	7,945	5,592 (70.37%)	379 (4.77%)	40 (0.50%)
**BIRTH COHORTS - MISCARRIAGE NEVER**				
1879-1900	233	2 (0.86%)	10 (4.29%)	6 (2.58%)
1900-1925	9,866	3,115 (31.57%)	800 (8.11%)	109 (1.10%)
1925-1950	19,760	13,599 (68.82%)	2,045 (10.35%)	149 (0.75%)
1950-1975	34,670	23,980 (69.19%)	1,398 (4.04%)	159 (0.46%)
**TOTAL - MISCARRIAGE EVER**				
1879-1977	22,549	14,039 (62.26%)	1,802 (7.99%)	173 (0.77%)
**TOTAL - MISCARRIAGE NEVER**				
1879-1977	64,529	40,696 (63.07%)	4,253 (6.59%)	423 (0.66%)
**TOTAL - ANY**				
1879-1977	87,078	54,735 (62.86%)	6,055 (6.95%)	596 (0.68%)

Comment #8

Lastly, I was thinking about the sequence variants in the final set of 25 genes with the deficit of homozygosity. Are any of those variants at a detectable frequency in other populations from say TOPMED or 1000 Genomes?

Response to comment #8

As a response to the reviewer's question, we have added a Supplementary table showing the allele frequencies for sequence variants with evidence of homozygous deficit in TOPMED and Gnomad (see Supplementary Table 6).

All 22 sequence variants with evidence of homozygous deficit are detected in GnomAD (141,456 individuals) and TOPMED (genomes Freeze 8, n = 132,345 individuals) databases but at a lower average frequency than in the population we studied (1.52 million individuals). This is especially the case for variants with founder frequency in Iceland and Finland, which are populations that are either absent from these databases, as is the case of Iceland (0 versus 154,085 Icelanders), or with fewer individuals than we have access to in the population we studied (12,562 versus 224,737 Finns).

Page 9, paragraph 2, lines 249-252, now read (changes underlined):

"The allele frequency distribution of the underlying pLOF and missense variants ranges from < 0.001% to 1.4% across the six populations <u>and are detectable but rarer in publicly available exome and genome sequence databases</u> (Table 1, <u>Supplementary Table 6, and Supplementary Fig. 3</u>) (see Supplementary Discussion for details)."

Supplementary Table 6 (See also the separate Excel file with all supplementary tables).

S. Table 6. Allele frequency of homozygous deficit variants in the GnomAD and TOPMed databases.

				CURRENT STUDY		TOPMED	GNOMAD	GNOMAD POPMAX			
POSITION (HG38)	HGVS	MAX CONSEQUENCE	GENE	MAF (%)	DATASET	MAF (%)	MAF (%)	STRATA	MAF (%)	HOM OBS	HOM EXP
chr11:71435840_C_G	c.964-1G>C	spl acceptor	*DHCR7*	1.4,0.43,0.35,0.27,1.1,0.19	I,D,N,S,U,F	0.4	0.4	nfe	0.6	0	4
chr16:8811153_G_A	p.Arg141His	missense	*PMM2*	0.58,0.6,0.51,0.51,0.6,0.69	I,D,N,S,U,F	0.3	0.4	nfe	0.5	0	2
chr16:88663006_C_T	c.70+5G>A	spl region	*MVD*	0.24,0.22,0.16,0.24,0.41	I,D,N,S,U	0.3	0.2	nfe	0.3	0	0.6
chr5:44811105_T_G	p.Ile233Arg	missense	*MRPS30*	0.43,0.43,0.56,0.41,0.34,1	I,D,N,S,U,F	0.2	0.3	fin	1	0	3
chr12:82354490_CTTACTTGT_C	c.561_564+4delACAAGTAA	spl donor	*CCDC59*	0.45,0.41,0.52,0.34,0.31,0.23	I,D,N,S,U,F	0.1	0.2	nfe	0.2	0	0.2
chr1:15506000_T_G	p.His237Pro	missense	*CASP9*	0.28,0.31,0.26,0.34,0.23,0.31	I,D,N,S,U,F	0.1	0.1	nfe	0.2	0	0.4
chr12:123633862_G_A	p.Met1?	start lost	*GTF2H3*	0.25,0.33,0.48,0.21,0.13	I,D,S,U,F	0.04	0.1	nfe	0.3	0	0.7
chr19:49862369_A_G	c.1029+2T>C	spl donor	*PNKP*	0.3,0.21,0.23,0.13,0.17	I,D,N,S,U	0.08	0.1	nfe	0.2	0	0.2
chr3:81648854_A_G	c.691+2T>C	spl donor	*GBE1*	0.33,0.23,0.31,0.22,0.13	I,D,N,S,U	0.06	0.09	nfe	0.2	0	0.2
chr1:92333464_TGAGTA_T	p.Lys512ValfsTer20	frameshift	*RPAP2*	0.64	I	0.005	0.003	nfe	0.007	0	4.7×10^{-4}
chr12:57796461_C_T	p.Gln307Ter	stop gained	*TSFM*	0.12,1.1	S,F	0.01	0.2	fin	1	0	3
chr20:62198729_AG_A	p.Gly191AlafsTer14	frameshift	*MTG2*	0.14,0.14,0.7	I,U,F	0.06	0.2	fin	1	0	2
chr1:111459496_C_T	p.Arg185Ter	stop gained	*ATP5PB*	1	I	0.002	0.0007	nfe	0.002	0	7.4×10^{-6}
chr8:37848595_C_T	c.214+1G>A	spl donor	*BRF2*	0.83	I	0.0008	0.001	nfe	0.003	0	6.9×10^{-5}
chr1:119033158_TG_T	p.His279MetfsTer4	frameshift	*WARS2*	0.77	I		0.0004	nfe	0.0009	0	4.4×10^{-6}
chr19:11554278_AC_A	p.Gln23HisfsTer15	frameshift	*ELOF1*	0.76	I	0.001	0.0007	nfe	0.002	0	7.4×10^{-6}
chr9:2837222_ATT_A	p.Lys87IlefsTer12	frameshift	*PUM3*	0.71	I	0.005	0.005	amr	0.01	0	4.9×10^{-4}
chr7:141649323_A_ATAAC	p.Ile348AsnfsTer38	frameshift	*AGK*	0.61	I	0.001	0.0004	nfe	0.002	0	4.4×10^{-5}
chr1:91520185_T_G	p.Tyr412Ter	stop gained	*CDC7*	0.58	I	0.002	0.002	nfe	0.003	0	6.9×10^{-5}
chr6:52058349_G_A	p.Arg496Ter	stop gained	*PKHD1*	0.48	F	0.004	0.07	fin	0.7	0	0.6
chr11:125900000_A_G	p.Asp211Gly	missense	*HYLS1*	0.81	F	0.01	0.1	fin	1	0	1
chr9:128536414_G_A	p.Arg569His	missense	*GLE1*	0.85	I	0.01	0.03	nfe	0.04	0	0.02

Reviewer 3: Minor comments, Main Text

Comment #1

Abstract: uses pLOF but the term is not defined

Response to comment #1

We now use the term “predicted loss-of-function” instead of “pLOF” in the abstract.

Comment #2

Line 204: missing period after publications

Response to comment #2

The missing punctuation mark in line 204 is corrected.

Comment #3

Line 236: Tables should be Table

Response to comment #3

Line 236 has been corrected to replace "Tables" with "Table".

Comment #4

Line 271: The authors say “from most population databases” which databases are being referred to here. Can the authors include citations?

Response to comment #4

In response to the reviewer's concerns, we have added a reference to the gnomAD database to the sentence ending on Line 271 and refer to Supplementary Table 6, and Supplementary Fig. 3.

Comment #5

Line 276: extra space before Frameshift

Response to comment #5

Line 276 has been edited to remove the extra space.

Comment #6

Line 421: extra space before We

Response to comment #6

Line 421 has been revised to remove the extra space.

Reviewer 3: Minor comments, Supplement

Comment #1

General comment for the tables, it is really hard to read the text underneath them, can font size be increased?

Response to comment #1

It is appreciated that the reviewer brought this to our attention. To improve the readability of the footnotes of the main tables, the font size has been increased. Additionally, all supplementary tables have been combined into a single Excel document for the convenience of the reader.

Comment #2

For supplementary tables 9-11 can the authors include how p-values are computed and a one sentence declarative for caption?

Response to comment #2

We have now added one-sentence declarative and information on how P-values are calculated to supplementary tables 7, 8, and 9 (previously supplementary tables 9, 10, and 11).

Comment #3

Supplementary figure 1 has a typo, the last sentence is a fragment and I think is a key assumption for the paper.

Response to comment #3

In the caption of supplementary figure 1, the typo "homozygous counts" has been corrected to "homozygote counts".

The sentence fragment “Deviations from random mating within a population will only increase the number of observed homozygotes through the Wahlund effect and thus expose more homozygotes to selection.” has been removed.

As this is a key assumption of the paper, as the reviewer has pointed out, we would like to emphasize that assumption is made on page 7, paragraph 2, lines 191-199, where Supplementary Figure 1 is also referred to: "It is well established that deviations from random mating within a population (such as inbreeding or stratification), tend to increase the number of observed homozygotes[16]. For sequence variants with phenotypic impact, this can increase the number of individuals with homozygote genotypes that are exposed to selection. In our data, there is an excess of observed homozygotes compared to the expected number of homozygotes under HWE, with rare variants showing the greatest relative excess (Supplementary Fig. 1 and Supplementary Table 2)."

Comment #4

Supplementary figure 2, I think observed should be lower case

Response to comment #4

Supplementary figure 2 now uses lowercase letters for the word observed.

Comment #5

Page 28 second section: first sentence in first paragraph homozygote is misspelled and first sentence in second paragraph is missing a parenthesis for VEP

Response to comment #5

On In the first paragraph of page 28 of the supplement, "homozygout" has been replaced with "homozygot," and the parenthesis for "VEP" has been added.

Comment #6

Page 29: typo-was less than 10% the expected I think it should be 10% of the

Response to comment #6

On page 29 of the supplement, "was less than 10% the expected number of homozygotes" has been changed to "was 10% or less of the expected number of homozygotes".

Comment #7

Page 31: extra space before We in first paragraph

Response to comment #7

The extra space before "We" after the first paragraph has been removed from page 31 of the supplement.

Comment #8

Page 32: what does MP abbreviation stand for?

Response to comment #8

On page 32 of the supplement, the MP abbreviation has now been defined as follows (changes underlined): "Embryonic lethal genes were identified by querying the Mammalian Phenotype Ontology (MP) terms ".

Comment #9

Pages 35-36: The first few sentences of the Incomplete homozygous deficit section have many typos

Response to comment #9

Please refer to Reviewer 1, minor comments 6, 7, and 8.

Comment #10

Page 36: first new paragraph in the second to last sentence, differences is misspelled

Response to comment #10

Please refer to Reviewer 1, minor comment 7.

REVIEWERS' COMMENTS

Reviewer #1 (Remarks to the Author):

All the review comments have been addressed by the authors in the revision and the manuscript can be accepted for publication.

Reviewer #2 (Remarks to the Author):

The authors have adequately addressed my major concerns. I particularly appreciate the power analysis and comparison between couples with both or only one parent being a carrier, the results of which clearly strengthen the conclusions. That said, I still have some minor comments on the revised version, mostly about the FDR calculation and presentation.

1. In Supplementary Information lines 360-369, the authors explained how FDR is calculated from the observed numbers of variants with deficit of homozygosity (DOH) in intergenic and putatively functional variants, but the description is inaccurate or over-complicated in some places. First, the sentence in lines 360-362 is stated incorrectly: as it currently reads, the numerator is the number of intergenic variants with deficit of homozygosity for both L and H, but actually the numerator is the number of intergenic with DOH and functional variants with DOH, respectively. In addition, the formula for calculating FDR can be re-written as FDR=L/H, which is simpler and easier to understand; alternatively, the authors could write PPV=1-FDR=1-L/H. The authors should include this formula in the main text somewhere (either Results or Fig 2 legend), and I would advise usage of more informative symbols for the variables (for example, f_intergenic and f_impact instead of L and H; f indicates the fraction nature of this quantity).

2. Figure 2: Reviewer 3 asked about the unexpected fluctuation in some of the curves. The authors correctly pointed out that this reflects the statistical uncertainty in the FDR estimates. To show this under-appreciated uncertainty, could the authors add the 95% or 90% confidence interval to each of the estimates? Although the independence assumption is oversimplifying, it provides a reasonable approximation.

3. Lines 222-225: contrary to what is stated in this sentence, the FDR measures the fraction of positive cases (i.e., variants with deficit of homozygosity) due to chance. Instead, it is one minus FDR (or PPV, positive predictive power) that measures the fraction of positive cases due to selection. Similarly, please correct the caption of Supplementary Table 2, which currently reads “FDR: true positive rate of homozygous deficit relative to intergenic variants calculated as (fraction homo def - fraction homo def intergenic)/fraction homo def intergenic”.

4. In line 210, the authors defined “deficit of homozygosity” as Obs/Exp<0.1 and used this term henceforth throughout the paper. I am slightly concerned that some readers might miss this definition and thus mistake “deficit of homozygosity” as any significant deficit (which may or may not be remarkable in magnitude). Could the authors rephrase it as “strong deficit of homozygosity” or “severe deficit of homozygosity” to highlight the stringent criteria?

5. The numbers in Table 3 do not match the odds ratio numbers and associated confidence intervals in the main text (lines 329-335). Also, the enrichment and p-values for the 14 genes excluding DHCR7 are not included in Table 3. Please check and make sure that the text and table are up to date. As a side note, it is unclear that N=57 in line 328 is the number of miscarriages.

6. Lines 152-154: for a rare variant present in homozygous form in one per million individuals, shouldn’t it be found in roughly two per thousand individuals (two times the allele frequency)?

7. Ref 13, Karczewski et al (2020), has now been published. Please update the reference information.

Reviewer #3 (Remarks to the Author):

The authors have adequately addressed my comments. The new manuscript has improved.

My only minor suggestions:

1) Line 125 in main text has an extra space after the "."

2) Line 464 in main text has an extra space after "comparison,"

3) Table 1 goes off of the page

4) There are still sections of the supplement where the fonts change (see page 22-23). I think the supplement needs another careful read through

5) The pdf for the supplementary tables only has one table and it is cut-off, so it might be better to keep tables as excel files?

Point-by-point answer to reviewers

We thank the three reviewers for their comments. We have made changes to the manuscript according to their comments.

All changes can be reviewed in track changes. Line numbers below refer to edits in the manuscript with no track changes.

Reviewer #1 (Remarks to the Author):

All the review comments have been addressed by the authors in the revision and the manuscript can be accepted for publication.

Reviewer #2 (Remarks to the Author):

The authors have adequately addressed my major concerns. I particularly appreciate the power analysis and comparison between couples with both or only one parent being a carrier, the results of which clearly strengthen the conclusions. That said, I still have some minor comments on the revised version, mostly about the FDR calculation and presentation.

Reviewer 2: Minor comments

Comment #1

In Supplementary Information lines 360-369, the authors explained how FDR is calculated from the observed numbers of variants with deficit of homozygosity (DOH) in intergenic and putatively functional variants, but the description is inaccurate or over-complicated in some places. First, the sentence in lines 360-362 is stated incorrectly: as it currently reads, the numerator is the number of intergenic variants with deficit of homozygosity for both L and H, but actually the numerator is the number of intergenic with DOH and functional variants with DOH, respectively. In addition, the formula for calculating FDR can be re-written as FDR=L/H, which is simpler and easier to understand; alternatively, the authors could write PPV=1-FDR=1-L/H. The authors should include this formula in the main text somewhere (either Results or Fig 2 legend), and I would advise usage of more informative symbols for the variables (for example, f_intergenic and f_impact instead of L and H; f indicates the fraction nature of this quantity).

Response to comment #1

As suggested by the reviewer, we have revised and simplified our description and formula for the FDR, as well as adopting more informative symbols for the variables.

Lines 637-643 in the Methods section of the manuscript now reads (changes underlined):

"Within one of these ranges of expected number of homozygotes under HWE, let f_intergenic and f_pav denote the fraction of variants with a deficit of homozygosity among intergenic sequence variants, and protein-altering sequence variants, respectively. A false discovery rate (FDR) was estimated by dividing the fraction of intergenic sequence variants with a strong deficit of homozygosity by the fraction of protein-altering sequence variants with a strong deficit of homozygosity:

$$FDR = \frac{f\_intergenic}{f\_pav}$$ "

Furthermore, we have included the formula for describing the relationship between the FDR and PPV in the main text and to the legend of Fig. 2.

Lines 219-225 in the manuscript now reads (changes underlined):

"After binning variants based on the expected number of homozygotes under HWE and functional impact, we compared the fraction of protein-altering variants (f_pav) with a strong deficit of homozygosity in each bin to the intergenic one (f_intergenic) to derive a false discovery rate (FDR = f_intergenic/f_pav) (Fig. 2 and Supplementary Table 2). "

Figure 2 legend line 1041 now reads:

“Figure 2. False discovery rate (FDR) for a strong deficit of homozygosity relative to intergenic variants in the combined set of 1.52 million individuals of North-Western European descent (Denmark, Finland, Iceland, Norway, Sweden, and the UK). After binning variants based on the expected number of homozygotes and functional impact, the fraction of protein-altering variants with a strong deficit of homozygosity (f_pav) in each bin was compared to that of intergenic variants (f_intergenic) to estimate an FDR (FDR = f_intergenic/f_pav).”

Comment #2

Figure 2: Reviewer 3 asked about the unexpected fluctuation in some of the curves. The authors correctly pointed out that this reflects the statistical uncertainty in the FDR estimates. To show this under-appreciated uncertainty, could the authors add the 95% or 90% confidence interval to each of the estimates? Although the independence assumption is oversimplifying, it provides a reasonable approximation.

Response to comment #2

For each estimate in Figure 2, we have added a 95% confidence interval. To simplify the figure, we have removed the low-impact variants from the plot (see below). For variants of all impact classes, we provide a 95% confidence interval for the FDR and the fraction of homozygous deficit in Supplementary Tables 2 and 4. Our FDR confidence intervals were calculated using the AECI method, which estimates a confidence interval for a ratio of two independent Poisson rates (PMID: 31707912).

Lines 647-649 in the Methods section of the manuscript now reads (changes underlined):

"FDR confidence intervals were calculated using the ad-hoc approximate-estimate CI (AECI) method, which estimates a confidence interval for the ratio of two independent Poisson rates[66]"

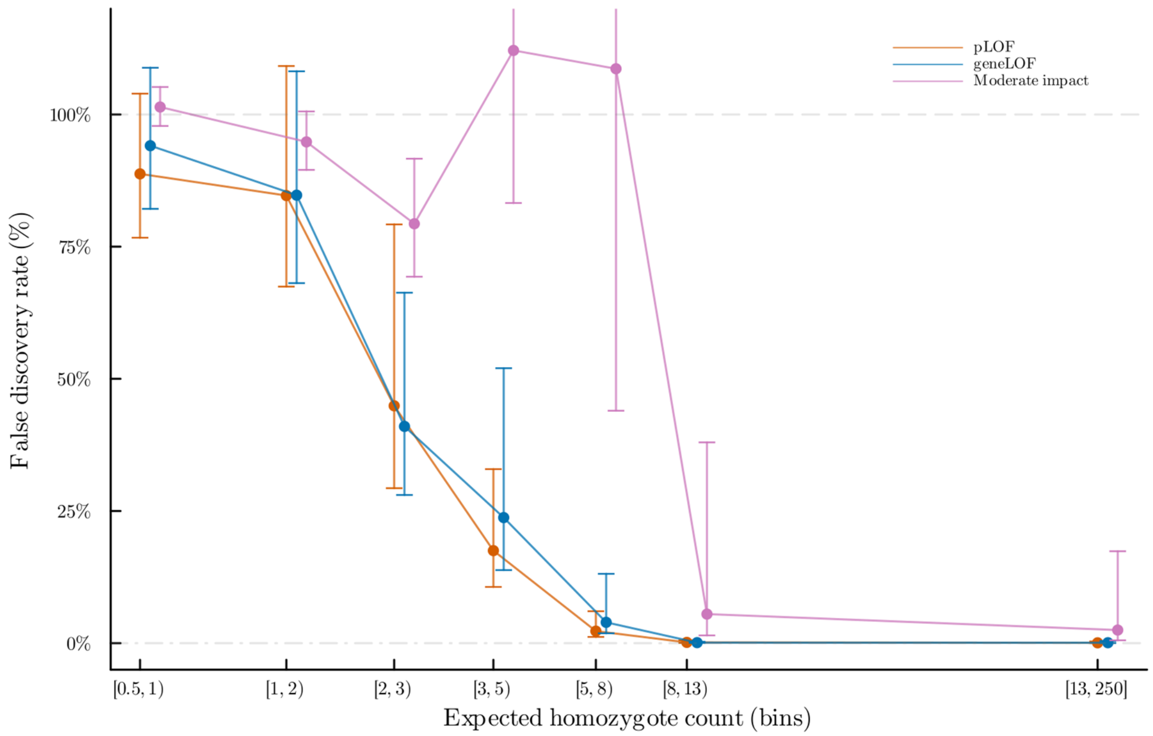

Figure 2. False discovery rate (FDR) for a strong deficit of homozygosity relative to intergenic variants in the combined set of 1.52 million individuals of North-Western European descent (Denmark, Finland, Iceland, Norway, Sweden, and the UK). After binning variants based on the expected number of homozygotes and functional impact, the fraction of protein-altering variants with a strong deficit of homozygosity (f_pav) in each bin was compared to that of intergenic variants (f_intergenic) to estimate an FDR (FDR = f_intergenic/f_pav). One minus the FDR estimates the fraction of homozygous deficit variants within each bin due to negative selection (1 - FDR = positive predictive power (PPV) = 1 - f_intergenic/f_pav). FDR confidence intervals were calculated using the AECI method.

Comment #3

Lines 222-225: contrary to what is stated in this sentence, the FDR measures the fraction of positive cases (i.e., variants with deficit of homozygosity) due to chance. Instead, it is one minus FDR (or PPV, positive predictive power) that measures the fraction of positive cases due to selection. Similarly, please correct the caption of Supplementary Table 2, which currently reads "FDR: true positive rate of homozygous deficit relative to intergenic variants calculated as (fraction homo def - fraction homo def intergenic)/fraction homo def intergenic".

Response to comment #3

We have corrected the statement in the main text and in the caption of Supplementary Table 2.

Lines 223-226 in the manuscript now reads:

"One minus the FDR estimates the fraction of homozygous deficit variants within each bin due to negative selection rather than by chance, under the assumption that homozygosity for intergenic variants is effectively neutral (1 - FDR = positive predictive power (PPV) = 1 - f_intergenic/f_pav). **"**

Comment #4

In line 210, the authors defined “deficit of homozygosity” as Obs/Exp<0.1 and used this term henceforth throughout the paper. I am slightly concerned that some readers might miss this definition and thus mistake “deficit of homozygosity” as any significant deficit (which may or may not be remarkable in magnitude). Could the authors rephrase it as “strong deficit of homozygosity” or “severe deficit of homozygosity” to highlight the stringent criteria?

Response to comment #4

In response to the reviewer's suggestion, the definition has been rephrased as "strong deficit of homozygosity" and is now used throughout the manuscript and supplementary information.

Lines 210-212 now reads:

“We considered a variant to have a strong deficit of homozygosity if we observed 10% or less of predicted homozygotes[17,18] based on observed heterozygote frequency and the assumption of HWE within populations”.

Comment #5

The numbers in Table 3 do not match the odds ratio numbers and associated confidence intervals in the main text (lines 329-335). Also, the enrichment and p-values for the 14 genes excluding DHCR7 are not included in Table 3. Please check and make sure that the text and table are up to date. As a side note, it is unclear that N=57 in line 328 is the number of miscarriages.

Response to comment #5

The reviewer's attention to this issue is appreciated. After recalculating miscarriage risks for couples where either both or only one parent are carriers compared to non-carrier couples, Table 3 values were not updated by mistake. As of now, Table 3 values are updated to correspond to the updated values in both the main text and Supplementary Table 12. Additionally, odds ratios and p-values are now included for the 14 genes other than DHCR7.

In addition, lines 329-332 have been changed to reflect that N=57 represents the number of miscarriages among carrier couples.

“Carrier mothers were at increased risk of ever experiencing a miscarriage if the father was a carrier compared to mothers from non-carrier couples matched on year of birth and number of pregnancies (OR = 1.93 [95% CI: 1.35-2.74], $P = 2.4 \times 10^{4}$, N couples = 140, N miscarriage = 57) (Table 3 and Supplementary Table 12)**”**

Comment #6

Lines 152-154: for a rare variant present in homozygous form in one per million individuals, shouldn’t it be found in roughly two per thousand individuals (two times the allele frequency)?

Response to comment #6

The reviewer is correct. Due to changes made during the review process, this sentence ended up displaying incorrect information.

Lines 153-155 now reads:

“In a randomly-mating population, a rare variant present in one per five hundred individuals is expected to be present in one per million in a homozygous state”

Comment #7

Ref 13, Karczewski et al (2020), has now been published. Please update the reference information.

Response to comment #7

The reference information has been updated in the manuscript:

Manuscript References lines 812-813 now reads:

“13. Karczewski, K. J. et al. The mutational constraint spectrum quantified from variation in 141,456 humans. Nature 581, 434–443 (2020).”

Reviewer #3 (Remarks to the Author):

The authors have adequately addressed my comments. The new manuscript has improved.

My only minor suggestions:

Reviewer 3: Minor comments

Comment #1

Line 125 in main text has an extra space after the "."

Response to comment #1

Extra space on line 125 has been removed.

Comment #2

Line 464 in main text has an extra space after "comparison,"

Response to comment #2

Extra space on line 464 has been removed.

Comment #3

Table 1 goes off of the page

Response to comment #3

The width of Table 1 has been adjusted to fit the page

Comment #4

There are still sections of the supplement where the fonts change (see page 22-23). I think the supplement needs another careful read through

Response to comment #4

In the supplementary information, font changes have been corrected, and the document has been carefully read thorugh.

Comment #5

The pdf for the supplementary tables only has one table and it is cut-off, so it might be better to keep tables as excel files?

Response to comment #5

All supplementary tables are provided as a separate excel file (see Supplementary Data files).
